# Ready or Not; A Narrative Synthesis of Sports Medicine Practitioners' Practices During Return to Play in the Management of Musculoskeletal Injuries

**DOI:** 10.1002/ejsc.70071

**Published:** 2025-10-25

**Authors:** Megan Chetty, Gregory Roe, Ben Jones, Sharief Hendricks

**Affiliations:** ^1^ Division of Physiological Sciences and Health through Physical Activity, Lifestyle and Sport Department of Human Biology Faculty of Health Sciences University of Cape Town Cape Town South Africa; ^2^ Carnegie Applied Rugby Research Centre Leeds Beckett University Leeds UK; ^3^ England Performance Rugby Football League Manchester UK; ^4^ Research and Rugby Development PREM Rugby Ltd London UK; ^5^ School of Behavioural and Health Sciences Australian Catholic University Brisbane Australia

**Keywords:** criteria, decision‐making, musculoskeletal injury, practices, return to play, sports medicine practitioner

## Abstract

The purpose of this narrative synthesis was to identify and synthesise the literature focused on sports medicine practitioners' (SMPs) decision‐making practices during return to play (RTP) after musculoskeletal (MSK) injury. Using the Preferred Items for Reporting Systematic Reviews and Meta‐Analyses guidelines, four electronic databases were searched from the start of the database to July 2024 using terms related to SMPs and RTP in MSK injury. The Appraisal Tool of Cross‐Sectional Studies (AXIS) and the Johanna Briggs Institute (JBI) critical appraisal tools were used to assess the overall quality of the identified studies. A narrative synthesis format was considered the most appropriate methodological approach to review and synthesise the pool of literature. Data synthesis included the participating SMPs profession, study sample size, injury location, activity level, RTP outcome measures and results. Data were further characterised by the RTP practices for specific MSK injuries, including spine, shoulder, wrist, hand, hip, knee, ankle and foot. Eighty‐seven (*n* = 87) publications were identified based on the inclusion and exclusion criteria. Forty‐seven percent (*n* = 41) of the studies focused on surgeon practices and 29.9% (*n* = 26) reported practices of multidisciplinary teams (MDTs). Almost half of all studies (40.2%; *n* = 35) addressed knee injuries, 85.7% (*n* = 30/35) specific to the anterior cruciate ligament (ACL). Eighty‐three percent (*n* = 34/41) of medical doctors consider injury and postoperative timelines compared to other SMP groups (47.8%; *n* = 22/46). Multidisciplinary team studies report the use of psychological readiness (50%; *n* = 13/26) and sport‐specific testing (38.5%; *n* = 10/26) criteria in RTP studies. Functional assessment and strength are reported in at least 50% of physiotherapist (*n* = 18) and rehabilitation specialist (*n* = 2) studies. Reference to RTP frameworks, guidelines and protocols in RTP decision‐making was found in less than 20% of the publications. Studies addressing input from other SMPs to assist decision‐making was also found in less than 20% of the studies despite research. From these studies, shared decision‐making with an athlete‐centred approach is preferred. The type of sport and the ambition of the athlete were the biggest influencing factors on decision surrounding RTP both reported in 26.4% (*n* = 23) of all SMP studies. This suggests an athlete‐centred approach to SMPs RTP decision‐making. Similar RTP criteria was used between practitioner groups, although criteria were weighted differently, due to the different scopes of practice and complexity surrounding RTP decisions. This review provides context for future research to assist and guide RTP decision‐making practices after MSK injuries. The need for clear, uncomplicated and practical definitions, guidelines, protocols and criteria will improve the RTP process and reduce the risk of reinjury after MSK injury. This review included all study designs and there was heterogeneity in the analysed studies, which can be viewed as a limitation.

**Trial Registration:**

The review was registered with the International Prospective Register of Systematic Reviews (PROSPERO) (registration ID: CRD42021270638) and OSF registries (registration doi: https://doi.org/10.17605/OSF.IO/DKQ7V).

## Highlights

- The majority of the studies to date focus on the knee, especially ACL injury.
- Although SMPs may consider similar criteria during RTP, each criteria is weighted differently. Return to play healing time was the criteria considered the most frequently.
- The present review highlights that without collaboration and practical tools with clear strategies, SMPs may struggle to be consistent in RTP practice.

## 1 Introduction

Return to play (RTP) is the term used in sports medicine to describe the process of safely returning an athlete back to training and competition after an injury (Ardern, Glasgow, et al. 2016). In addition to safely returning an athlete back to training and competition, RTP aims to prevent the injury from reoccurring and optimise performance (Ardern, Glasgow, et al. 2016). The RTP process can start at the time of injury and end when the athlete returns to performance during competition (Draovitch et al. 2022). The RTP process is complex, influenced by physical, psychological and contextual factors and often involves a range of stakeholders with different objectives (Ardern, Glasgow, et al. 2016; Draovitch et al. 2022; Matheson et al. 2011; Shrier et al. 2014; Drust et al. 2014; Beam 2002; Yung et al. 2022). For example, for a competition final, a coach may feel pressurised to return a high‐profile injured athlete to training sooner, whereas the sports medicine practitioner may require more time to reduce the risk of reinjury (Ardern, Glasgow, et al. 2016; Draovitch et al. 2022; Shrier 2015). As such, decisions during the RTP process are challenging and may have performance, mental health and financial implications (Ardern, Glasgow, et al. 2016; Matheson et al. 2011; Shrier et al. 2014; Drust et al. 2014; Beam 2002).

Sport medicine practitioners (e.g., surgeons, sports medicine doctors and physiotherapists) (SMPs) are key decision‐makers during the RTP process (Ardern, Glasgow, et al. 2016; Shrier et al. 2014; Ardern et al. 2016; Hagglund et al. 2016; Ellapen et al. 2017). Sports medicine practitioners are required to use their clinical judgement to decide when an athlete can RTP without medical restriction (Yung et al. 2022, 2024; Hecksteden et al. 2023). In view of the complex and multi‐stakeholder nature of the RTP process and its implications, a number of RTP frameworks, guidelines and clearance criteria have been proposed to help SMPs navigate the decision‐making process (Ardern, Glasgow, et al. 2016; Yung et al. 2024; Hecksteden et al. 2023; Shrier et al. 2015; Buckthorpe et al. 2019). For example, the Strategic Assessment of Risk and Risk Tolerance (StARRT) framework describes three steps to help estimate the risks of different short‐term and long‐term outcomes associated with RTP, and factors that may affect what should be considered an acceptable risk within a particular context (Shrier 2015; Shrier et al. 2015; Shrier and et al. 2017; Shrier et al. 2017). In addition to RTP frameworks, SMPs may have specific practices related to their sport and context (Ardern, Glasgow, et al. 2016; Shrier et al. 2014; Drust et al. 2014; Ardern et al. 2016; Blanch and Gabbett 2016; McCall et al. 2017; Burgess 2011). For instance, SMPs can work in different geographical locations, sporting environments and have different specialisations which can create sociocultural and practice differences (Hess and Meyer 2022; Betsch et al. 2022; Shultz et al. 2013).

A musculoskeletal injury (MSK‐I) is a general term that includes damage to muscles, bones, tendons, joints, ligaments and other soft tissues (Shrier et al. 2015; Creighton et al. 2010; Gimigliano et al. 2021). Musculoskeletal injury is one of the most common injury risks associated with sport participation and carries a high economic burden (Pereira et al. 2019; Gebert et al. 2020; Kisser and Bauer 2012). Reinjuries can occur if the RTP process is not managed appropriately (Ardern, Glasgow, et al. 2016; Hagglund et al. 2016; Creighton et al. 2010; Fournier 2015; Hägglund et al. 2018; Beardmore et al. 2005; Fuller et al. 2007). Around 20% of injuries in elite sport are reinjuries (Hägglund et al. 2018; Williams et al. 2017). The risk of reinjury is higher in amateur levels and in the early stages of RTP (Hägglund et al. 2018; Williams et al. 2017). Williams and colleagues found 42% of reinjuries occur within the first 2 months of returning to rugby (Hägglund et al. 2018; Williams et al. 2017). In elite football, this number could escalate to 77% (Hägglund et al. 2018; Williams et al. 2017).

To date, many papers have explored different RTP criteria and frameworks in MSK‐I (Ardern, Glasgow, et al. 2016; Draovitch et al. 2022; Shrier et al. 2014; Drust et al. 2014; Ardern et al. 2016; Buckthorpe et al. 2019; Blanch and Gabbett 2016; McCall et al. 2017; Burgess 2011). A number of studies have also been conducted to determine what RTP criteria and frameworks are being used by SMPs in MSK‐I practice (Hess and Meyer 2022; van der Horst et al. 2017; von Aesch et al. 2016; Grassi et al. 2016; Vascellari et al. 2017; Aguilaniu et al. 2021). Although these studies acknowledge the multi‐stakeholder nature of the RTP process, they are typically conducted within a specific medical profession (e.g., surgeons or physiotherapists) (Matheson et al. 2011; Ardern et al. 2016; Beardmore et al. 2005; W. A. Fausett et al. 2022; Testoni et al. 2013). Musculoskeletal injury RTP criteria and practices may also vary within a specific profession, with no consensus on when an athlete may RTP (Ardern, Glasgow, et al. 2016; Shrier et al. 2014; Ardern et al. 2016; Dunlop et al. 2020; Health Professions Council of South Africa (HPCSA) 2016). To move MSK‐I RTP research forward, it is important to understand current practices of SMPs both within and across professions (Matheson et al. 2011; van der Horst et al. 2017). Understanding the focus of current research on SMPs, criteria used and factors influencing MSK‐I RTP decisions can help standardisation and collaboration within sports medicine. Therefore, the purpose of this narrative synthesis is to identify and synthesise the research on SMPs' RTP practices when working with athletes with MSK‐I.

## 2 Methods

A narrative synthesis format was considered the most appropriate methodological approach to review and synthesise the pool of literature. A narrative synthesis refers to an approach to a systematic review where findings from multiple studies are synthesised using primarily words and text (Popay et al. 2006; Burger et al. 2020). Narrative syntheses can be used to review and assess quantitative and qualitative data and involve a systematic and predefined search strategy with a focus on producing a more textual synthesis versus other types of systematic reviews such as quantitative meta‐analyses (Popay et al. 2006; Burger et al. 2020).

### 2.1 Protocol and Registration

This review was conducted in accordance with the Preferred Items for Reporting Systematic Reviews and Meta‐Analyses guidelines (Page et al. 2021). The PRISMA guideline is a valued way of reporting literature within systematic reviews and using the PRISMA guideline assisted with the planning and documentation of this systematic review (Page et al. 2021; Shamseer et al. 2015) (Supporting Information S1: Table 1 and 2).

The review was registered with the International Prospective Register of Systematic Reviews (PROSPERO) (registration ID: CRD42021270638) (Booth et al. 2012; Page et al. 2018) and OSF registries (registration doi: 10.17605/OSF.IO/DKQ7V) (Kocher and Riegelman 2018). Registration prevented unnecessary duplication, allowing for transparency and minimising bias (Shamseer et al. 2015; Grant and Booth 2009).

### 2.2 Search Strategy

The databases searched included PubMed, EBSCOhost, Scopus and the Web of Science using the search terms and keywords for the period from the start of the database to July 2024. The search key included: (‘RTP’ OR ‘return to sport’ OR ‘return to performance’ OR ‘return to training’ OR ‘return to competition’ OR ‘return to participation’ OR ‘return to function’) AND (‘knowledge’ OR ‘attitude*’ OR ‘behaviour*’ OR ‘behaviour*’ OR ‘practice*’ OR ‘perception’ OR ‘opinion’) (e.g., searches, see Supporting Information S1: Table 3).

**FIGURE 1:**
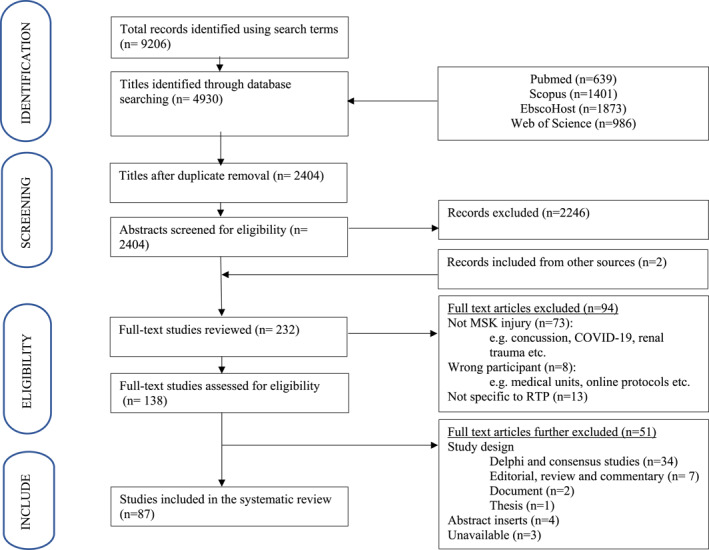
Selection process of included studies.

The initial search identified 8202 articles (Figure 1). The author list, title and abstract of each article was captured into a Microsoft Excel spreadsheet. Once duplicates were removed, the study titles were screened for potential inclusion, which reduced the number to 2352. The remaining articles' abstracts were then screened using eligibility inclusion and exclusion criteria (below).

### 2.3 Inclusion Criteria

Studies related to RTP with the population specific to SMPs were included.

The following inclusion criteria were required.

▪ *Full‐text journal articles published in English*
▪ All quantitative and qualitative controlled trials and cohort, including qualitative only, case–control, case series and cross‐sectional observational studies, published up to *July 2024.*
▪ The article's sample population included a SMP—including doctors, surgeons, nurses, physiotherapists, physical therapists, biokineticists and athletic trainers.
▪ Only studies of SMPs involved in the RTP process, management of an athlete's RTP after MSK‐I (i.e., specific to an area or injury) and studies generalised to MSK‐I RTP practices (i.e., not specific to any area or injury) were included.
▪ All articles referring to RTP decision‐making practices were included. The broad range of outcomes included but were not limited to:
  ◦ RTP frameworks, protocols, guidelines and practices
  ◦ Clearance criteria for unrestricted sport participation, such as clinical measures, including strength, psychological measures (such as PROs), tissue healing measures (such as timelines, MRI scans and ultrasounds), and specific RTP tests (such as hop tests or fitness test batteries)
  ◦ Other influencing factors such as type of sport, athlete pressure and specialisation

### 2.4 Exclusion Criteria

▪ Articles in a language other than English.
▪ Scoping reviews, literature reviews, systematic reviews and nonresearch papers, such as consensus documents, text and opinion papers, were excluded from the analyses.
▪ Articles related to RTP decision‐making and general RTP practices for head injury, such as concussion and COVID‐19, were excluded.
▪ Studies where the study participants were not healthcare or SMPs, such as athletes, coaches, parents and governing bodies, were excluded.

All avenues, such as available author contact, to obtain full access to the texts of the preliminarily identified articles were followed before they were excluded.

Based on the inclusion and exclusion criteria, 138 full‐text articles were identified for review.

The reference lists of the 138 articles were also checked for possible inclusion; however, no additional articles were identified. The 138 full‐text articles were reviewed for final eligibility by MC and SH. Any uncertainties regarding the inclusion of a particular article were resolved by discussion between the two reviewers until consensus was reached. After full‐text reviewing, a further 51 articles were excluded.

### 2.5 Data Extraction

The following data were extracted from all included studies: author/s, year of publication, journal of publication, title, abstract and source, study's sample size, participant characteristics or profession were identified and recorded.

Information on SMPs' perception of their current practices, including the models, frameworks, guidelines, protocols and clearance criteria that guided their practices in RTP decision‐making after MSK‐I, were also identified for each study. In addition, the MSK‐Is were classified according to the type and location of the injury, including the spine, shoulder, wrist, hand, hip, knee, ankle and foot. Next, any factors or difficulties that influenced the SMPs' RTP decision‐making practice were identified.

### 2.6 Quality Appraisal

The Appraisal Tool of Cross‐Sectional Studies (AXIS) and the Johanna Briggs Institute (JBI) critical appraisal tools were used to assess the overall quality of the identified studies. The AXIS tool was used to critically appraise the cross‐sectional studies (Supporting Information S1: Table 6). The applicable JBI critical appraisal checklist, for either qualitative research (Supporting Information S1: Table 8) or cohort studies (Supporting Information S1: Table 10), was used as an additional tool to further assess seven of the articles (Ma et al. 2020) (Supporting Information S1: Table 8. The AXIS tool, developed in 2016 and containing 20 items, does not include a numerical scale that creates an output quality score (Downes et al. 2016). The tool is designed for the user to assess individual characteristics with some degree of subjectivity (Downes et al. 2016). For a strong rating of 20 to reflect for an article, a ‘no’ response is required for questions 13 and 19. For a comprehensive overview of the literature and to provide the range of methodological quality in the field, studies were not excluded based on their quality appraisal.

## 3 Data Synthesis and Reporting

This narrative synthesis used a tabular synthesis with a narrative accompaniment (Grant and Booth 2009). Articles were categorised according to the different roles and scopes of practice (Health Professions Council of South Africa (HPCSA) 2016), namely, surgeons, physicians, physiotherapists, rehabilitation specialists or multidisciplinary teams. Then, they were divided according to the following: Nonspecific MSK‐I RTP practice (including injury vignettes, frameworks and ethical considerations) and specific MSK‐I practice. Specific MSK‐I practice include the area and type of injury, such as knee, shoulder or spine. In addition, the analysis included the sport, athlete population, tools used, decision‐makers and influencing factors such as athlete pressure, athlete characteristics and practitioner experience and qualifications. A detailed description of each study included within this review is found in Supporting Information S1: Table 5, which is also divided into the different SMP, practice and area of injury.

Due to the inclusion of qualitative and quantitative studies, varying definitions and terminologies used within studies, thematic content analysis was used to group themes (Griffin et al. 2021; Braun and Clarke 2006). The researcher analysed each study within the review. Practices, assessments and descriptors were analysed and reported individually or grouped if terminology overlapped (Griffin et al. 2021; Braun and Clarke 2006). The studies were organised into different SMP groups. After analysing the study of each practitioner group, data from the results section of each study was summarised in bullet point form. After all results from each study was pulled, a map of each practitioner group was made. The results of each study was then listed into headings around the map. The data were then organised further into a table to allow for more transparent synthesis of the data.

Variability of terminology within the studies meant that data needed to be collated into headings, for example: swelling and effusion were grouped under the main heading—swelling. If multiple descriptors (such as function and functional tests) were used within one study, they were grouped together for the purpose of this review. Pain and strength assessments were assessed individually as most studies considered them in isolation. The following assessments and descriptors were grouped.

- Clinical examination: Included studies that reported a combination of ROM, special tests, swelling, pain, objective physical tests, clinical assessment and general appearance
- ROM: Includes mobility and flexibility tests
- Swelling: Includes effusion
- Healing time: Includes pathophysiological criteria and state of healing
- Imaging: Includes investigations and laboratory tests
- Limb asymmetry: Includes thigh girth circumference, bilateral differences
- Functional assessment: Includes terms used in studies, such as function, functional state, functional capacity, movement patterns and assessments, such as hop test, single‐leg squat, lower limb biomechanics and upper limb biomechanical assessment.
- Sport‐specific tests: Includes test batteries, fitness tests, agility tests and sport‐specific tests or skill such as the Vail sport test and seated shotput test
- Checklist of criteria: Includes studies that reported a combination of a clearance criteria including time‐based approach, routine clinical examination, functional assessment and sport skill
- Psychological readiness: These assessments included psychological or psychiatric evaluation, mental tests, subjective feelings and wellness questionnaires
- Special tests: Includes mechanical instability, absence of instability and specific joint tests such as Lachman's, anterior drawer, pivot shift and anterior apprehension
- Proprioception: Includes balance
- Patient‐reported outcome measures (PROMs): Includes clinical scoring systems and subjective surveys
- Training load: Includes training load, global positioning systems (GPS), aerobic‐anaerobic capacity, endurance, ability to train or warm up and modified training
- Strength: Includes gym maximal tests, isolated strength tests using Oxford scale, hand held dynamometry and isokinetic testing
- Athlete‐related factors: Includes age, type of sport or training load, injury complexity and athlete aspirations such as level of sport
- Medical doctors: Includes orthopaedic and trauma surgeons, physicians and general practitioners
- Rehabilitation specialists: Includes athletic trainers, rehabilitation specialists and biokineticists
- Exercise specialists: Includes sport scientists, physiologists, strength and conditioning specialists
- Practitioner‐related factors: Included speciality, experience, qualifications, previous athletic experience and previous case complications
- Multidisciplinary team: Includes combinations of different sports medicine and performance team practitioners
- High demand sport: A term used for any sport that uses high intensity, change of direction, plyometrics and contact load (Mitchell et al. 1994). Many studies grouped different sporting codes together. For example, soccer and volleyball (Silva et al. 2011) or gymnastics, speed skating and rowing (Barrette and Harman 2020)
- Shared‐decision making: Includes input from other practitioners

### 3.1 Ethical Considerations

This review was approved by the University of Cape Town, Faculty of Health Sciences Human Research Ethics Committee (HREC/REF 207/2022)

## 4 Results

The 87 articles that met the inclusion criteria were cross‐sectional in design and represented a total of 12,688 SMPs. Sixty (*n* = 71) of the studies were quantitative, 14 were qualitative, one was a mixed method study design and one had a cohort study design component (Table 1). Most of the articles (79.3%; *n* = 69) were published between 2014 and 2024. Forty‐one of the 87 selected studies assessed medical doctors RTP practices (*n* = 38, 43.7%) (Betsch et al. 2022; Grassi et al. 2016; Vascellari et al. 2017; Arliani et al. 2019; Coşkunsu et al. 2010; Ebert et al. 2020; B. J. Erickson et al. 2014, 2015; Farber et al. 2014; Feller et al. 2002; Mahnik et al. 2013; Marshall et al. 2019; McRae et al. 2011; Petersen and Zantop 2013; Sherman et al. 2021; Bakowski et al. 2020; Thaler et al. 2021; Ukogu et al. 2020; France et al. 2016; Morganti et al. 2001; Abla et al. 2011; Ho et al. 2021; Schrock et al. 2018; Dy et al. 2013; Sharareh et al. 2021; Moore et al. 2021; Golant et al. 2012; Domb et al. 2014; Vu‐Han et al. 2021; Dams et al. 2019; Vertullo and Nunley 2002; Beck et al. 2022; Glattke et al. 2022; Hobusch et al. 2020; Pandey et al. 2022; Yokoe et al. 2021; Backer et al. 2024; Sambare et al. 2021), 26 studies (29.9%) incorporated a multidisciplinary team (Shrier et al. 2014; Shrier et al. 2017; Hess and Meyer 2022; Shultz et al. 2013; Beardmore et al. 2005; Dunlop et al. 2020; Barrette and Harman 2020; Ebert et al. 2019; Lambert et al. 2021; Mazer et al. 2010; Boudier‐Reveret et al. 2011; Wörner et al. 2018; Balcı and Ülkar 2020; P. J. Read et al. 2018; Yeomans et al. 2018; Lyng et al. 2020; Riendeau et al. 2015; Johnson‐Lynn and Townshend 2017; Chen et al. 2022; Green et al. 2022; Geldenhuys et al. 2023; Horan et al. 2023; Müller et al. 2024; D. Read and Rosenbloom 2024; Maher et al. 2024; Aguilaniu et al. 2023), 18 (20.7%) included physiotherapists (von Aesch et al. 2016; W. A. Fausett et al. 2022; Silva et al. 2011; Aquino et al. 2021; Greenberg et al. 2018; van Melick et al. 2020; Mendonca et al. 2020; Witjes et al. 2018; Brindisino et al. 2021; Nasser et al. 2021; Kaye et al. 2021; Korakakis et al. 2021; Brindisino et al. 2023; Gauthier et al. 2023; Valente et al. 2023; Alshehri et al. 2024; Pulver et al. 2024; Tondelli et al. 2024), three (3.4%) included sport or team physicians (Aguilaniu et al. 2021; McAdams et al. 2016; L. C. Anderson and Gerrard 2005) and two studies reported on rehabilitation specialists (Di Trani Lobacz et al. 2016; McVeigh and Pack 2015). Of the 41 studies addressing medical doctors, 38 studies (92.7%) assessed surgeons (primarily orthopaedic and trauma surgeons) (Betsch et al. 2022; Grassi et al. 2016; Vascellari et al. 2017; Arliani et al. 2019; Coşkunsu et al. 2010; Ebert et al. 2020; B. J. Erickson et al. 2014, 2015; Farber et al. 2014; Feller et al. 2002; Mahnik et al. 2013; Marshall et al. 2019; McRae et al. 2011; Petersen and Zantop 2013; Sherman et al. 2021; Bakowski et al. 2020; Thaler et al. 2021; Ukogu et al. 2020; France et al. 2016; Morganti et al. 2001; Abla et al. 2011; Ho et al. 2021; Schrock et al. 2018; Dy et al. 2013; Sharareh et al. 2021; Moore et al. 2021; Golant et al. 2012; Domb et al. 2014; Vu‐Han et al. 2021; Dams et al. 2019; Vertullo and Nunley 2002; Beck et al. 2022; Glattke et al. 2022; Hobusch et al. 2020; Pandey et al. 2022; Yokoe et al. 2021; Backer et al. 2024; Sambare et al. 2021). Twenty studies (23.0%%) were noninjury specific (Table 1), whereas the majority of injury specific studies focused on the knee, especially anterior cruciate ligament (ACL) injury (Betsch et al. 2022; von Aesch et al. 2016; Grassi et al. 2016; Vascellari et al. 2017; W. A. Fausett et al. 2022; Arliani et al. 2019; Coşkunsu et al. 2010; Ebert et al. 2020; B. J. Erickson et al. 2014, 2015; Farber et al. 2014; Feller et al. 2002; Mahnik et al. 2013; Marshall et al. 2019; McRae et al. 2011; Petersen and Zantop 2013; Sherman et al. 2021; Bakowski et al. 2020; Thaler et al. 2021; Glattke et al. 2022; Pandey et al. 2022; Ebert et al. 2019; Lambert et al. 2021; Lyng et al. 2020; Aquino et al. 2021; Greenberg et al. 2018; van Melick et al. 2020; Mendonca et al. 2020; Witjes et al. 2018; Kaye et al. 2021; Korakakis et al. 2021; Alshehri et al. 2024; Pulver et al. 2024; Tondelli et al. 2024; McVeigh and Pack 2015) (Table 2).

**TABLE 1:** Noninjury specific RTP practices including focus of the study.

RTP practices	Focus of the study
(23.0%; *n* = 20) (Shrier et al. 2014; Shrier et al. 2017; Hess and Meyer 2022; Shultz et al. 2013; Beardmore et al. 2005; Silva et al. 2011; Barrette and Harman 2020; Schrock et al. 2018; Hobusch et al. 2020; Mazer et al. 2010; Boudier‐Reveret et al. 2011; P. J. Read et al. 2018; Yeomans et al. 2018; Riendeau et al. 2015; Chen et al. 2022; Geldenhuys et al. 2023; Horan et al. 2023; Müller et al. 2024; D. Read and Rosenbloom 2024; L. C. Anderson and Gerrard 2005)	Injury vignettes (*n* = 4) (Schrock et al. 2018; Hobusch et al. 2020; Mazer et al. 2010; Boudier‐Reveret et al. 2011)
	General approach to RTP practice (*n* = 12) (Shrier et al. 2014; Shrier et al. 2017; Hess and Meyer 2022; Shultz et al. 2013; Beardmore et al. 2005; Silva et al. 2011; Barrette and Harman 2020; P. J. Read et al. 2018; Yeomans et al. 2018; Chen et al. 2022; Geldenhuys et al. 2023; Horan et al. 2023; Müller et al. 2024; D. Read and Rosenbloom 2024)
	Ethics (*n* = 2) (Riendeau et al. 2015; L. C. Anderson and Gerrard 2005)

**TABLE 2:** Area and type of injury addressed in the narrative synthesis.

General Injury area	Specific Injury location	Injury type
Lower limb injury (58.6%; *n* = 51) Betsch et al. 2022; von Aesch et al. 2016; Fausett et al. 2022; Testoni et al. 2013; Barrette et al. 2020; Arliani et al. 2019; Coşkunsu et al. 2010; Ebert et al. 2020; B. J. Erickson et al. 2014, 2015; Farber et al. 2014; Feller et al. 2002; Mahnik et al. 2013; Marshall et al. 2019; McRae et al. 2011; Petersen and Zantop 2013; Sherman et al. 2021; Bakowski et al. 2020; Thaler et al. 2021; Domb et al. 2014; Vu‐Han et al. 2021; Dams et al. 2019; Vertullo and Nunley 2002; Beck et al. 2022; Glattke et al. 2022; Pandey et al. 2022; Yokoe et al. 2021; Ebert et al. 2019; Lambert et al. 2021; Balcı et al. 2020; Lyng et al. 2020; Johnson‐Lynn and Townshend 2017; Green et al. 2022; Aguilaniu et al. 2023; Aquino et al. 2021; Greenberg et al. 2018; van Melick et al. 2020; Mendonca et al. 2020; Witjes et al. 2018; Nasser et al. 2021; Kaye et al. 2021; Korakakis et al. 2021; Valente et al. 2023; Alshehri et al. 2024; Pulver et al. 2024; Tondelli et al. 2024; Di Trani Lobacz et al. 2016; McVeigh and Pack 2015	Pelvis/hip (*n* = 3)	Hip arthroscopy (*n* = 2) (Domb et al. 2014; Wörner et al. 2018)
		Hip arthroplasty (*n* = 1) (Vu‐Han et al. 2021)
	Knee (40.2%; *n* = 35)	ACL (*n* = 30) (Betsch et al. 2022; von Aesch et al. 2016; Grassi et al. 2016; Vascellari et al. 2017; Fausett et al. 2022; Arliani et al. 2019; Coşkunsu et al. 2010; Ebert et al. 2020; Erickson et al. 2014; Erickson et al. 2015; Farber et al. 2014; Feller et al. 2002; Mahnik et al. 2013; Marshall et al. 2019; McRae et al. 2011; Petersen et al. 2013; Sherman et al. 2021; Glattke et al. 2022; Pandey et al. 2022; Ebert et al. 2019; Lambert et al. 2021; Aquino et al. 2021; Greenberg et al. 2018; van Melick et al. 2020; Kaye et al. 2021; Korakakis et al. 2021; Alshehri et al. 2024; Pulver et al. 2024; Tondelli et al. 2024; McVeigh et al. 2015)
		Knee arthroplasty (*n* = 2) (Thaler et al. 2021; Witjes et al. 2018)
		Meniscus (arthroscopic procedures) (*n* = 1) (Bakowski et al. 2020)
		Patella tendinopathy (*n* = 1) (Mendonca et al. 2020)
		Osgood–Schlatter's disease (*n* = 1) (Lyng et al. 2020)
	Posterior thigh (*n* = 5)	Proximal hamstring tendinopathy (*n* = 1) (Nasser et al. 2021)
		Hamstring strain injury (*n* = 4) (Dunlop et al. 2020; Balcı et al. 2020; Valente et al. 2023; Di Trani Lobacz et al. 2016)
	Lower leg injuries (*n* = 1)	Calf strain injury (*n* = 1) (Green et al. 2022)
	Ankle and foot (*n* = 7)	Ankle sprains (*n* = 4) (Aguilaniu et al. 2021; Beck et al. 2022; Yokoe et al. 2021; Aguilaniu et al. 2023)
		Foot and ankle trauma (*n* = 1) (Johnson‐Lynn et al. 2017)
		Achilles tendon injury (*n* = 1) (Dams et al. 2019)
		Ankle and foot arthrodesis (*n* = 1) (Vertullo et al. 2002)
Upper limb injury (*n* = 9) (Dy et al. 2013; Sharareh et al. 2021; Moore et al. 2021; Golant et al. 2012; Maher et al. 2024; Brindisino et al. 2021; Brindisino et al. 2023; Gauthier et al. 2023)	Upper extremity injuries (*n* = 1)	(*n* = 1) (Gauthier et al. 2023)
	Shoulder (*n* = 7)	Arthroplasty (*n* = 2) (Golant et al. 2012; Brindisino et al. 2023)
		Glenohumeral instability (*n* = 3) (Sharareh et al. 2021; Moore et al. 2021; Maher et al. 2024)
		Rotator cuff repair (*n* = 1) (Brindisino et al. 2021)
	Elbow (*n* = 1)	Ligament (*n* = 1) (Sambare et al. 2021)
	Hand and wrist (*n* = 1)	Multiple injury scenarios (*n* = 1) (Dy et al. 2013)
Trunk (*n* = 7) (Ukogu et al. 2020; France et al. 2016; Morganti et al. 2001; Abla et al. 2011; Ho et al. 2021; Backer et al. 2024; McAdams et al. 2016)	Spine (*n* = 6)	Cervical injury/surgery (*n* = 4)^a^ (Ukogu et al. 2020; France et al. 2016; Morganti et al. 2001; Abla et al. 2011)
		Lumbar surgery^a^ (*n* = 2) (Abla et al. 2011; Backer et al. 2024)
		Scoliosis (*n* = 1) (Ho et al. 2021)
	Thorax (*n* = 1)	Chondral fractures (*n* = 1) (McAdams et al. 2016)

^a^ one study addressed both cervical and lumbar injury (Witjes et al. 2018).

The quality of the articles varied with a mean of 16 and a range of 6–20 based on the AXIS tool (Supporting Information S1: Tables 4 and 7). Of the 87 articles, 75.9% (*n* = 66) had a moderate to strong score, meeting most (16–20 out of the possible 20) of the AXIS tool criteria. The AXIS tool does not use an overall score to evaluate articles but rather allows the assessor to evaluate individual aspects of the article. However, a score of 16–20 has been deemed moderate to strong in previous studies (Downes et al. 2016; Wong et al. 2018). Twenty (23.0%) articles ranged between 11 and 15 out of the possible 20. Of the 87 articles, only 18.4% (*n* = 16) met top scores of 19 or 20 out of the 20 criteria. One article scored below 10 out of 20 (McAdams et al. 2016).

Out of the 87 studies appraised using the AXIS checklist criteria, 47.1% (*n* = 41) failed to address and categorise nonresponders (Q7) and documented response rates varied between 1% and 100%. Uncertainty with response rate bias (Q13) was found in 32.2% (*n* = 28), and 60.9% (*n* = 53) of the studies had a lack of information about nonresponders (Q14). Thirty‐three (37.9%) studies used measurements or instruments that had not been previously trialled, piloted or published in the literature (Q9). Ethics and consent or board approval were mentioned in 83.9% (*n* = 73) of the studies; however, the ethical considerations of 16.1% (*n* = 14) of the studies were lacking (Coşkunsu et al. 2010; Feller et al. 2002; Mahnik et al. 2013; Sherman et al. 2021; France et al. 2016; Morganti et al. 2001; Moore et al. 2021; Domb et al. 2014; Beck et al. 2022; Hobusch et al. 2020; Pandey et al. 2022; Backer et al. 2024; Johnson‐Lynn and Townshend 2017; Witjes et al. 2018).

Fifteen of the 87 articles were also appraised using additional tools (Hess and Meyer 2022; Betsch et al. 2022; von Aesch et al. 2016; Barrette and Harman 2020; Lyng et al. 2020; Riendeau et al. 2015; Chen et al. 2022; Green et al. 2022; van Melick et al. 2020; Nasser et al. 2021; Kaye et al. 2021; McVeigh and Pack 2015), namely, the JBI qualitative and cohort tools. This was due to the extra appraisal tools being more specific to the study design of the 15 articles. Both appraisal tools generated strong appraisal scores with a mean of 9.7 (*n* = 14) and 11 (*n* = 1), respectively (Supporting Information S1: Tables 9 and 11).

Table 3 shows the area, practice and criteria used for RTP. Collectively, for all SMPs, healing time was the most widely reported criteria used to determine RTP followed by strength and functional assessment (Table 3). Below SMPs included in the review that determine RTP were grouped into subheadings: medical doctors, physiotherapists, rehabilitation specialists and MDTs. The detail characteristics of all included studies are shown in Supporting Information S1: Table 5.

**TABLE 3:** The number of studies by sports medicine practitioners, study description and frequency of RTP criteria reported within those studies.

Medical doctors (*n* = 4591 participants)	Number of studies (*n* = 41)	Physiotherapists (*n* = 5281 participants)	Number of studies (*n* = 18)	Rehabilitation specialists (*n* = 1364 participants)	Number of studies (*n* = 2)	Multidisciplinary team (*n* = 3280 participants)	Number of studies (*n* = 26)
Generalised RTP practice	3	Generalised RTP practice	1			Generalised RTP practice	16
Area		Area		Area		Area	
Spine	6	Shoulder	3	Hamstring	1	Shoulder	1
Thorax	1	Hamstring	2	Knee	1	Hip	1
Shoulder	3	Knee	12			Hamstring	2
Elbow	1					Knee	3
Hand and wrist	1					Lower leg	1
Hip	2					Ankle and foot	2
Knee	19						
Ankle and foot	5						

Criteria	Reported frequency within the studies (*n* = 41) (%)	Criteria	Reported frequency within the studies (*n* = 18) (%)	Criteria	Reported frequency within the studies (*n* = 1) (%)	Criteria	Reported frequency within the studies (*n* = 26) (%)
Healing time	34 (82.9%)	Functional assessment	13 (72.2%)	Functional assessment	1 (50%)	Psychological readiness	13 (50%)
Strength	12 (30.6%)	Strength	12 (66.7%)	Strength	1 (50%)	Sport specific tests	10 (38.5%)
Functional assessment	11 (25%)	Healing time	12 (66.7%)	Palpation	1 (50%)	Functional assessment	10 (38.5%)
Clinical examination	10 (26%)	PROM	10 (55.6%)	Imaging	1 (50%)	Healing time	10 (38.5%)
Pain	10 (19.4%)	ROM	9 (50%)	ROM	1 (50%)	Training load	8 (30.8%)
Special tests	8 (19.4%)	Sport specific tests	8 (44.4%)	Special tests	1 (50%)	Strength	8 (30.8%)
Imaging	7 (19.4%)	Psychological readiness	8 (44.4%)	Psychological readiness	1 (50%	Pain	8 (30.8%)
PROM	7 (16.7%)	Checklist of criteria	4 (22.2%)	RTP protocol	1 (50%)	ROM	7 (26.9%)
ROM	7 (16.7%)	Special tests	4 (22.2%)			Clinical examination	5 (19.2%)
Checklist of criteria	5 (13.9%)	Pain	4 (22.2%)			RTP protocols	4 (15.4%)
Sport specific tests	5 (13.9%)	Proprioception	4 (22.2%)			Checklist of criteria	4 (15.4%)
Swelling	5 (11.1%)	Clinical examination	2 (11.1%)			PROM	3 (11.5%)
Psychological readiness	3 (8.3%)	Swelling	2 (11.1%)			Imaging	2 (7.7%)
Limb asymmetry	3 (8.3%)	Limb asymmetry	3 (16.7%)			Proprioception	2 (7.7%)
RTP guideline	3 (5.6%)	Prevention programme	2 (11.1%)			Swelling	2 (7.7%)
RTP protocol	2 (2.7%)	RTP guidelines	2 (11.1%)			RTP guidelines	2 (7.7%)
Proprioception	1 (%)	Training load	2 (11.1%)			Limb asymmetry	1 (3.8%)
		Imaging	1 (5.5%)				

### 4.1 Medical Doctors

Out of the 41 medical doctor studies, a description of the athletic profile was included in 23 studies (Betsch et al. 2022; Grassi et al. 2016; Aguilaniu et al. 2021; Arliani et al. 2019; Erickson et al. 2014; Erickson et al. 2015; Farber et al. 2014; Petersen et al. 2013; Sherman et al. 2021; Bakowski et al. 2020; Ukogu et al. 2020; France et al. 2016; Abla et al. 2011; Ho et al. 2021; Schrock et al. 2018; Dy et al. 2013; Moore et al. 2021; Vertullo et al. 2002; Hobusch et al. 2020; Backer et al. 2024; Sambare et al. 2021; McAdams et al. 2016; Anderson et al. 2005). Athletes in contact sports, including football, hockey and soccer, made up 56.5% (n = 13) of these 23 studies. Adolescent and young adult athletes (age range 15–21) were the focus of six articles (Bakowski et al. 2020; France et al. 2016; Ho et al. 2021; Moore et al. 2021; Beck et al. 2022; Hobusch et al. 2020).

Seventeen of the 41 studies focused on ACL injury and reconstruction (Betsch et al. 2022; Grassi et al. 2016; Vascellari et al. 2017; Arliani et al. 2019; Coşkunsu et al. 2010; Ebert et al. 2020; Erickson et al. 2014; Erickson et al. 2015; Farber et al. 2014; Feller et al. 2002; Mahnik et al. 2013; Marshall et al. 2019; McRae et al. 2011; Petersen et al. 2013; Sherman et al. 2021; Glattke et al. 2022; Pandey et al. 2022). Within ACL studies, 55.5% (Erickson et al. 2014) to 91.8% (Arliani et al. 2019) doctors permit RTP after a minimum of 6 months postinjury or surgery (Betsch et al. 2022; Grassi et al. 2016; Vascellari et al. 2017; Arliani et al. 2019; Coşkunsu et al. 2010; Ebert et al. 2020; Erickson et al. 2014; Erickson et al. 2015; Farber et al. 2014; Feller et al. 2002; Mahnik et al. 2013; Marshall et al. 2019; McRae et al. 2011; Petersen et al. 2013; Sherman et al. 2021; Glattke et al. 2022; Pandey et al. 2022). Healing time was the most widely considered criteria in RTP decisions within medical doctor studies (82.9%) (Betsch et al. 2022; Grassi et al. 2016; Vascellari et al. 2017; Arliani et al. 2019; Coşkunsu et al. 2010; Ebert et al. 2020; Erickson et al. 2014; Erickson et al. 2015; Farber et al. 2014; Feller et al. 2002; Mahnik et al. 2013; Marshall et al. 2019; McRae et al. 2011; Petersen et al. 2013; Sherman et al. 2021; Bakowski et al. 2020; Thaler et al. 2021; France et al. 2016; Abla et al. 2011; Ho et al. 2021; Schrock et al. 2018; Dy et al. 2013; Sharareh et al. 2021; Moore et al. 2021; Golant et al. 2012; Domb et al. 2014; Vu‐Han et al. 2021; Dams et al. 2019; Beck et al. 2022; Glattke et al. 2022; Pandey et al. 2022; Yokoe et al. 2021; Backer et al. 2024; Sambare et al. 2021). Pain was considered in a number of studies (Aguilaniu et al. 2021; McAdams et al. 2016; Anderson et al. 2005), with the use of analgesics to assist accelerating the RTP process (McAdams et al. 2016; Anderson et al. 2005).

Four studies considered the role of the decision‐maker (Betsch et al. 2022; Arliani et al. 2019; Marshall et al. 2019; Bakowski et al. 2020). Medical doctors often work in elite sport (Aguilaniu et al. 2021; McAdams et al. 2016; Anderson et al. 2005). Pressure to return athletes to play can create ethical dilemmas for sports medicine doctors especially as medical doctors feel primarily responsible in making RTP decisions (Anderson et al. 2005). Bakowski and colleagues found that 37% of surgeons believe that the surgeon was wholly responsible for RTP decisions, with 42% opting for shared decision‐making (SDM) between the surgeon and physiotherapist (Bakowski et al. 2020). A range of 8.2% (Arliani et al. 2019) to 24% (Marshall et al. 2019) of surgeons used the physiotherapist assessment for RTP clearance (Arliani et al. 2019; Marshall et al., 2019). Two studies mentioned the athlete’s involvement in decision‐making, with surgeons supporting RTP when the athlete was aware of the consequences (Dy et al., 2013; Vertullo et al., 2002). In 2022 Betsch and colleagues, surgeons emphasised the importance of using a more comprehensive multidisciplinary team (including physiotherapists, athletic trainers, strength and conditioning coaches, sport specific psychologists and sport scientists) to assist RTP decisions (Betsch et al., 2022).

### 4.2 Physiotherapists

Ten studies described the athletic profile (Silva et al., 2011; Aquino et al., 2021; Greenberg et al., 2018; van Melick et al., 2020; Nasser et al., 2021; Gauthier et al., 2023; Valente et al., 2023; Alshehri et al., 2024; Pulver et al., 2024; Tondelli et al., 2024) with three studies related to high demand sports such as soccer and volleyball (Silva et al., 2011; van Melick et al., 2020; Valente et al., 2023). Three studies reported that RTP criteria may be overlooked when returning athletes to play after injury (Aquino et al., 2021; van Melick et al., 2020; Mendonca et al., 2020). One study reported that 56% of the pivoting athletes were cleared to RTP without using RTP criteria (van Melick et al., 2020). Another study mentioned the use of a RTP guideline but did not describe the guideline in detail (von Aesch et al., 2016).

The role of RTP decision makers were considered in six studies (von Aesch et al. 2016; Silva et al. 2011; Aquino et al. 2021; Greenberg et al. 2018; Alshehri et al. 2024; Tondelli et al. 2024). Physiotherapists were thought to be involved in the RTP process of an injured athlete 100% of the time (Silva et al. 2011), however, between 10.9% (Silva et al. 2011) to 25.1% (Aquino et al. 2021) of physiotherapists would clear an athlete using their assessment in isolation. Thirteen percent (Aquino et al. 2021) and 14.5% (Silva et al. 2011) of physiotherapists felt that the medical doctor was responsible for clearance, respectively. Majority of physiotherapists ranging from 74.5% (Silva et al. 2011) to 80.1% (Greenberg et al. 2018) felt that the biggest contributor in RTP decisions comes from SDM practices between doctor and physiotherapist. Clearance from a MDT was mentioned in two studies with 62.1% (Aquino et al. 2021) and 79.2% (Tondelli et al. 2024) of physiotherapists recommending this collaborative clearance. An athlete‐centred approach with athletes being actively involved in decision‐making were mentioned within two studies (von Aesch et al. 2016; Kaye et al. 2021).

### 4.3 Rehabilitation Specialists

A limited number of studies made it difficult to establish a hierarchy of reported criteria (Table 3). In hamstring strain injuries, functional assessment and strength were the most significant criteria for rehabilitation specialists involved in RTP (Di Trani Lobacz et al. 2016). In ACL injury, the importance of education, psychological readiness and a RTP protocol are reported but detail on methods and criteria for RTP clearance are lacking (McVeigh et al. 2015). The athletic profile was not described for studies on rehabilitation specialists (Di Trani Lobacz et al. 2016; McVeigh et al. 2015).

### 4.4 MDTs

The composition of SMPs that made up the MDT varied between studies. Physiotherapists were included in 25 of the MDT studies (Shrier et al. 2014; Shrier et al. 2017b; Hess et al. 2022; Shultz et al. 2013; Beardmore et al. 2005; Dunlop et al. 2020; Barrette et al. 2020; Ebert et al. 2019; Lambert et al. 2021; Mazer et al. 2010; Boudier‐Reveret et al. 2011; Wörner et al. 2018; Balcı et al. 2020; Read et al. 2018; Yeomans et al. 2018; Lyng et al. 2020; Riendeau et al. 2015; Johnson‐Lynn et al. 2017; Chen et al. 2022; Green et al. 2022; Geldenhuys et al. 2023; Müller et al. 2024; Read et al. 2024; Maher et al. 2024; Aguilaniu et al. 2023). Doctors were included in 18 (69.2%) studies (Shrier et al. 2014; Shultz et al. 2013; Beardmore et al. 2005; Dunlop et al. 2020; Lambert et al. 2021; Mazer et al. 2010; Boudier‐Reveret et al. 2011; Wörner et al. 2018; Balcı et al. 2020; Yeomans et al. 2018; Lyng et al. 2020; Johnson‐Lynn et al. 2017; Chen et al. 2022; Geldenhuys et al. 2023; Müller et al. 2024; Read et al. 2024; Maher et al. 2024; Aguilaniu et al. 2023). Members of the sports science team grouped under exercise specialists were included in nine (53%) studies (Shrier et al. 2017b; Beardmore et al. 2005; Dunlop et al. 2020; Barrette et al. 2020; Ebert et al. 2019; Mazer et al. 2010; Boudier‐Reveret et al. 2011; Read et al. 2018; Riendeau et al. 2015). Chiropractors were included in three studies (Shrier et al. 2014; Shultz et al. 2013; Barrette et al. 2020), whereas massage therapists were included in two studies (Shrier et al. 2014; Dunlop et al. 2020). One study addressed medical personnel involved within football but did not specify practitioner groups (Horan et al. 2023).

The same combination of medical doctors, physiotherapists and rehabilitation specialists (athletic trainers) were included in three of the MDT studies (Beardmore et al. 2005; Mazer et al. 2010; Boudier‐Reveret et al. 2011). The combination of physiotherapists and doctor were included in seven studies (Shrier et al. 2017b; Lambert et al. 2021; Wörner et al. 2018; Balcı et al. 2020; Johnson‐Lynn et al. 2017; Maher et al. 2024; Aguilaniu et al. 2023). Twenty‐three percent (*n* = 6) of the studies in this review assessed an MDT of four or more healthcare professions namely medical doctors, physiotherapists, strength and conditioning specialists, chiropractors, sports massage therapists and rheumatologists (Shrier et al. 2014; Dunlop et al. 2020; Read et al. 2018; Lyng et al. 2020; Geldenhuys et al. 2023; Müller et al. 2024).

The profile of the athlete was described in *n* = 21 of the studies (Shrier et al. 2014; Shrier et al. 2017b; Hess et al. 2022; Shultz et al. 2013; Beardmore et al. 2005; Dunlop et al. 2020; Barrette et al. 2020; Ebert et al. 2019; Lambert et al. 2021; Mazer et al. 2010; Boudier‐Reveret et al. 2011; Read et al. 2018; Yeomans et al. 2018; Lyng et al. 2020; Riendeau et al. 2015; Chen et al. 2022; Green et al. 2022; Geldenhuys et al. 2023; Horan et al. 2023; Müller et al. 2024; Read et al. 2024) with all athletes involved in high demand sports (such as rugby, football, soccer, hockey, gymnastics or judo) making up 90.5% (*n* = 19) of those studies (Shrier et al. 2017b; Hess et al. 2022; Shultz et al. 2013; Beardmore et al. 2005; Dunlop et al. 2020; Barrette et al. 2020; Ebert et al. 2019; Lambert et al. 2021; Mazer et al. 2010; Boudier‐Reveret et al. 2011; Read et al. 2018; Yeomans et al. 2018; Riendeau et al. 2015; Chen et al. 2022; Green et al. 2022; Geldenhuys et al. 2023; Horan et al. 2023; Müller et al. 2024; Read et al. 2024). Adolescent and young adult athletes (age range 9–25) were found in 7 studies (Barrette et al. 2020; Lambert et al. 2021; Mazer et al. 2010; Boudier‐Reveret et al. 2011; Read et al. 2018; Lyng et al. 2020; Riendeau et al. 2015).

The role of the decision‐maker was considered in fourteen studies (Shrier et al. 2014; Hess et al. 2022; Beardmore et al. 2005; Dunlop et al. 2020; Barrette et al. 2020; Wörner et al. 2018; Riendeau et al. 2015; Chen et al. 2022; Green et al. 2022; Müller et al. 2024; Read et al. 2024; Maher et al. 2024). Shared decision‐making was specifically addressed in ten studies (Shrier et al. 2014; Hess et al. 2022; Dunlop et al. 2020; Wörner et al. 2018; Riendeau et al. 2015; Chen et al. 2022; Green et al. 2022; Müller et al. 2024; Read et al. 2024; Maher et al. 2024). The medical team, namely, doctors, physiotherapists and athletic trainers are most involved in RTP and ranked highest in assessing RTP readiness (Shrier et al. 2014; Beardmore et al. 2005; Dunlop et al. 2020; Wörner et al. 2018). The SDM approach was favoured by 80% of the medical team in a study (Dunlop et al. 2020). One study ranked physiotherapists best at administering the fitness testing procedures (Beardmore et al. 2005). Another study highlighted physicians as making the final RTP decision often with input from therapists (Table 4) (Riendeau et al. 2015). Two studies found that there was not always agreement between clinicians within the decision‐making process (Shrier et al. 2017b; Beardmore et al. 2005). Disagreements between the medical team, athlete, coach and management were a significant concern for SMPs (Shrier et al. 2014; Shrier et al. 2017b; Barrette et al. 2020; Riendeau et al. 2015; Chen et al. 2022).

**TABLE 4:** The reported frequency of influencing factors that sports medicine practitioners may consider when making RTP decisions.

Medical Doctors		Physiotherapists		Rehabilitation specialists		Multidisciplinary team	
Influencing factors	Reported frequency within studies (*n* = 41) (%)	Influencing factors	Reported frequency within studies (*n* = 18)	Influencing factors	Reported frequency within studies (*n* = 2)	Influencing factors	Reported frequency within studies (*n* = 26)
Athlete‐related factors		Athlete‐related factors		Athlete‐related factors		Athlete‐related factors	
Type of sport and position (hierarchy of risk)	14 (34.1%)	Age	3 (16.7%)	Type of sport and position (hierarchy of risk)	1 (50%)	Athlete aspirations incl. Level of play, expectation, pressure, career implications, experience	14 (53.8%)
Type of injury incl. Complexity of injury, surgery, risk of re‐injury, genetics	9 (22.0%)	Compliance	2 (11.1%)	Athlete aspirations incl. Level of play, expectation, pressure, career implications, experience	1 (50%)	Type of injury incl. Complexity of injury, surgery, risk of reinjury, genetics	8 (30.7%)
Athlete aspirations incl. Level of play, expectation, pressure, career implications, experience	7 (17.1%)	Financial (e.g., costs and insurance limitations)	2 (11.1%)	Social/psychological impact	1 (50%)	Type of sport and position (hierarchy of risk)	7 (26.9%)
Age	2 (4.9%)	Athlete aspirations incl. Level of play, expectation, pressure, career implications, experience	1 (5.5%)			Masking the injury (incl. Medications and supplements)	4 (15.4%)
Parental influence	1 (2.4%)	Type of sport and position (hierarchy of risk)	1 (5.5%)			Age	3 (11.5%)
Body mass index	1 (2.4%)					Protective equipment	3 (11.5%)
						Social/psychological impact	3 (11.5%)
						Parental influence	2 (7.7%)
						Limb dominance	2 (7.7%)
						Past injury history	2 (7.7%)
						Nutritional status	2 (7.7%)
Practitioner related factors		Practitioner‐related factors		Practitioner‐related factors		Practitioner‐related factors	
Qualification, specialisation, training	3 (7.3%)	Input from other sports medicine practitioners	6 (33.3%)	Lack of confidence	1 (50%)	Input from other sports medicine practitioners	10 (38.5%)
Previous surgery hx (complications)	2 (4.9%)	Practical limitations of assessment and treatment (e.g. time, costs and tariff system)	3 (16.7%)			Communication (e.g., knowing your athlete, safety and trust)	5 (19.2%)
Years of experience	2 (4.9%)	Qualification (e.g., specialisation, training, research)	2 (11.1%)			Qualification, specialisation and training	3 (11.5%)
Personal athletic experience	1 (2.4%)	Lack of support	2 (11.1%)			Isolated decision‐making or lack of support	2 (7.7%)
Different geographical area or culture of practice	1 (2.4%)	Experience with injury	1 (5.5%)			Different geographical area or culture of practice	1 (3.8%)
Surgeon demographics	1 (2.4%)	Years of experience	1 (5.5%)			Practical limitations of assessment and treatment (e.g., time, costs and tariff system)	1 (3.8)
Input from other sports medicine practitioners	1 (2.4%)						
Practical limitations of assessment and treatment (e.g. time, costs, tariff system)	1 (2.4%)						
Other		Other		Other		Other	
External pressure (e.g., coach and management)	2 (4.9%)	External pressure (e.g., coach and management)	2 (8.3%)	External pressure (e.g., coach and management)	1 (50%)	Competition schedule, seasonal or match pressure	13 (50%)
Team sport (e.g., player importance and squad depth)	1 (3.4%)	Use of resources (e.g., equipment, space, resources, literature and research barriers)	2			External pressure (e.g., coach and management)	10 (38.5%)
Competition schedule, seasonal or match pressure	1 (2.4%)					Team sport (e.g., player importance and squad depth)	6 (23.1%)
Conflict of interest	1 (2.4%)					Fear of litigation	3 (11.5%)
						Use of resources (e.g., equipment, space, resources, literature and research barriers)	3 (11.5%)
						Practical limitations of assessment and treatment (e.g., time restraints)	2 (7.7%)
						Conflict of interest	2 (7.7%)
						Ability to modify training	1 (3.8%)
						Financial loss	1 (3.8%)
						Language barriers	1 (3.8%)
						Lack of medical staff (e.g., using students, sharing of staff)	1 (3.8%)

## 5 Discussion

To our knowledge, this is the first review to synthesise the current practices of SMPs making RTP decisions after MSK‐I in athletes. Medical doctors are the most included SMP studied in RTP followed by MDT, physiotherapists and rehabilitation specialists. Previous research suggests that the medical doctor is responsible for RTP decisions (Matheson et al. 2011), so it is understandable that research has focused towards medical doctors, especially surgeons (Matheson et al. 2011; van der Horst et al. 2017). Sports medicine practitioners are involved in making RTP decisions in various ways. For example, a SMP may be solely responsible for the RTP decision or may prefer to share the responsibility with other members of the medical team, performance team, coaching team and athlete (Matheson et al. 2011; Shrier et al. 2014; Testoni et al. 2013; Ekstrand et al. 2019). Shared‐decision making occurs when the SMP and athlete work together to make a medical decision after carefully weighing both the risks and the benefits (Paul et al. 2022; Burns et al. 2023). However, in sports, an athlete's recovery often involves multiple SMPs, each contributing to the decision‐making process (Paul et al. 2022; Burns et al. 2023). Eighty percent (Dunlop et al. 2020) and 80.1% (Greenberg et al. 2018) of SMPs prefer making use of SDM (Matheson et al. 2011; Shrier et al. 2014; Testoni et al. 2013; Ekstrand et al. 2019). Shared decision‐making helps address multiple elements in the complex RTP environment as doctors, physiotherapists, rehabilitation specialists and coaches play different roles in RTP decisions (Hess et al. 2022; Beardmore et al. 2005). With that said, the integration of the different roles between professions within the RTP decision‐making process was not apparent. The referral pattern of the studies in this review often referred to medical doctors and physiotherapists. This may be due to doctors and physiotherapists ability to provide on‐field emergency care and association with injuries sustained whist playing sport (Beardmore et al. 2005; Silva et al. 2011; Yeomans et al. 2018). Referral to other practitioners within the sports medicine field, such as sports psychologists and nutritionists, were not found in this review.

The majority of the studies to date focus on the knee, especially ACL injury (Betsch et al. 2022; von Aesch et al. 2016; Grassi et al. 2016; Vascellari et al. 2017; Fausett et al. 2022; Arliani et al. 2019; Coşkunsu et al. 2010; Ebert et al. 2020; Erickson et al. 2014; Erickson et al. 2015; Farber et al. 2014; Feller et al. 2002; Mahnik et al. 2013; Marshall et al. 2019; McRae et al. 2011; Petersen et al. 2013; Sherman et al. 2021; Glattke et al. 2022; Pandey et al. 2022; Ebert et al. 2019; Lambert et al. 2021; Aquino et al. 2021; Greenberg et al. 2018; van Melick et al. 2020; Kaye et al. 2021; Korakakis et al. 2021; Alshehri et al. 2024; Pulver et al. 2024; Tondelli et al. 2024; McVeigh et al. 2015). This is in line with injury epidemiology research which shows knee injuries, in particular ACL injuries, accounts for majority of severe injuries in athletes participating in dynamic, change of direction and contact sports (Ardern et al. 2016a; Gimigliano et al. 2021; Glattke et al. 2022; Gottschalk et al. 2011; Darrow et al. 2009). Anterior cruciate ligament injuries also have a high risk of reinjury when returning to sport and pose an increased risk of secondary complications and economic burden (Sherman et al. 2021; Glattke et al. 2022). With that said, it is also worth noting that there is a lack of research in other injuries. For example, only seven studies focused on shoulder injuries (Sharareh et al. 2021; Moore et al. 2021; Golant et al. 2012; Maher et al. 2024; Brindisino et al. 2021; Brindisino et al. 2023; Gauthier et al. 2023) despite shoulder injuries being reported in between 18% to 90% of athletes in various sporting codes (Cools et al. 2021).

The findings of this review suggest that although SMPs may consider similar criteria during RTP, each criteria is weighted differently. Return to play healing time was the criteria considered the most frequently. This is not surprising as rehabilitation cannot start unless the injured area is able to heal (Matheson et al. 2011; Hägglund et al. 2018; Toohey et al. 2019). Beyond healing time, functional assessment and strength are the criteria reported most frequently within these studies. However, within each practitioner group, there is variety of each criteria indicating a lack of consensus within RTP practices.

Psychological readiness, although considered important within RTP research, was a difficult criteria to generalise within this review due to the overlap in terminology and lack of clarity within studies (McVeigh et al. 2015; Kuenze et al. 2020; Podlog et al. 2015; Podlog et al. 2011; Lentz et al. 2018). Psychological readiness was the most reported criteria considered within MDT studies (Shrier et al. 2014; Shultz et al. 2013; Beardmore et al. 2005; Dunlop et al. 2020; Ebert et al. 2019; Lambert et al. 2021; Wörner et al. 2018; Balcı et al. 2020; Read et al. 2018; Lyng et al. 2020; Green et al. 2022; Geldenhuys et al. 2023; Maher et al. 2024); however, when assessing the individual SMP groups, the use of psychological readiness was reported considerably less. On the other hand, PROMs are objective tools (such as questionnaires) used by practitioners to determine the athletes subjective injury status during rehabilitation and RTP (Glattke et al. 2022; Kuenze et al. 2020; Lentz et al. 2018). PROMs can be used to assess different subjective aspects associated with injury such as fear of movement, fear of reinjury, hardiness, current functional ability, feelings of instability or psychological return to sport readiness (McVeigh et al. 2015; Kuenze et al. 2020; Lentz et al. 2018; Gomez‐Piqueras et al. 2020). This suggests that there is an association between the use of PROM and psychological readiness (Korakakis et al. 2021; Kuenze et al. 2020; Lentz et al. 2018). In some studies, practitioners understand the psychological responses to injury; however, they may lack skills to assess and intervene in rehabilitation and RTP (Kaye et al. 2021; McVeigh et al. 2015).

Sports medicine practitioners most often considered the type of sport, type of injury and athlete aspirations before giving RTP advice. This finding is understandable when considering tissue healing and how much stress the injured tissue can withstand before succumbing to reinjury (Shrier et al. 2015). The demands of different sports also lead to different injury trends (McCall et al. 2017; Barrette et al. 2020; Grindem et al. 2018; Marsall et al. 2012; Erickson et al. 2017). External pressures from coaches, management and competition schedule create ethical dilemmas and challenges for SMPs making RTP decisions (Hess et al. 2022; Silva et al. 2011; Chen et al. 2022; Anderson et al. 2005; McVeigh et al. 2015). Education, open communication and working in an integrated MDT are strategies that SMPs can use to ease these anxieties (Hess et al. 2022; Silva et al. 2011; Chen et al. 2022; Anderson et al. 2005; McVeigh et al. 2015). Practitioner‐related factors, especially specialisation, may result in better use of RTP criteria and improved confidence in RTP decision‐making (Ukogu et al. 2020; Morganti et al. 2001; Ho et al. 2021; Moore et al. 2021; Vu‐Han et al. 2021; Dams et al. 2019; Johnson‐Lynn et al. 2017). Therefore, it is important for SMPs to use evidence‐based medicine and invest in staying up to date with research.

### 5.1 Medical Doctors

The natural course of healing relative to postoperative times makes healing time a logical factor to consider in RTP decisions for medical doctors (Grassi et al. 2016). Bone healing, especially in surgical cases, involving bony lesions needs to be considered for a successful surgical outcome (Abla et al. 2011; Moore et al. 2021). Time allows for the bone to remodel, tissue adaption to take place during rehabilitation, better function and more stress tolerance to withstand the sport load the injured athlete wants to return to (Shrier, 2015; Blanch et al. 2016; Della Villa et al. 2021; Bengtsson et al. 2020; Bisciotti et al. 2019). However, determining the optimal timeline for RTP is difficult. Many studies in this review highlight a lack of consensus on RTP times (Ukogu et al. 2020; Dy et al. 2013; Sharareh et al. 2021; Moore et al. 2021; Dams et al. 2019). Within the ACL studies, 55.47% (Erickson et al. 2014) to 91.8% (Arliani et al. 2019) surgeons tend to permit RTP after a minimum of 6 months postinjury or surgery (Betsch et al. 2022; Grassi et al. 2016; Vascellari et al. 2017; Arliani et al. 2019; Coşkunsu et al. 2010; Ebert et al. 2020; Erickson et al. 2014; Erickson et al. 2015; Farber et al. 2014; Feller et al. 2002; Mahnik et al. 2013; Marshall et al. 2019; McRae et al. 2011; Petersen et al. 2013; Sherman et al. 2021; Glattke et al. 2022; Pandey et al. 2022). This correlates with the current evidence showing an increased risk of a second ACL injury when athletes return to train before 6 months, and a significant reduction in risk every month after reaching the 6 month mark (Della Villa et al. 2021).

Although time is an important factor to consider, other criteria needs to be accounted for when making RTP decisions (Grassi et al. 2016; Petersen et al. 2013; Sherman et al. 2021). The report of strength, functional assessment, clinical examination and fitness testing were considered in less than a third of the medical doctor studies with a variety of usage within each criteria. This suggests that doctors are more likely to clear an athlete to RTP after a certain time frame rather than assessing criteria such as strength, function and fitness (Grassi et al. 2016; Arliani et al. 2019; Coşkunsu et al. 2010; Ebert et al. 2020; Feller et al. 2002; Marshall et al. 2019; Petersen et al. 2013; Bakowski et al. 2020; Abla et al. 2011; Joreitz et al. 2016). Having said that, in Australia, the consideration of strength within RTP criteria among medical doctors appears to have increased over the last two decades from 25% in 2001 (Feller et al. 2002) to 78.1% (Ebert et al. 2020) in 2020. Additionally, some studies noted a variety of medical doctors referred or assumed that the physiotherapist or rehabilitation specialist was conducting the RTP assessment (Betsch et al. 2022; Arliani et al. 2019; Ebert et al. 2020). The use of criteria‐based RTP clearance checklist may need to be continually emphasised to ensure modifiable risk factors, such as strength, function and training load, are considered and addressed fully when making RTP decisions (Grassi et al. 2016; Marshall et al. 2019; Petersen et al. 2013; Sharareh et al. 2021; Yokoe et al. 2021; Ebert et al. 2019). However, considering the majority of studies address the medical doctor and literature suggesting the doctor is most responsible (Matheson et al. 2011), future research could focus on understanding medical doctors’ barriers to RTP assessment.

Pressure to return athletes to play can create ethical dilemmas for medical doctors especially as medical doctors feel primarily responsible in making RTP decisions (Anderson et al. 2005). However, SDM is favoured in evidence‐based sports medicine and it is promising that doctors are collaborating with the hope of better RTP outcomes (Ardern et al. 2016a; Betsch et al. 2022; Dijkstra et al. 2017; Elwyn et al. 2012). Medical doctors also appear to be less influenced by external pressures within RTP and are more often influenced by athlete‐related factors and practitioner‐related factors (Betsch et al. 2022). Practitioner‐related factors, such as experience and speciality, highlight the importance of continuous education in the developing world of sports medicine (Ukogu et al. 2020; Abla et al. 2011; Ho et al. 2021; Dams et al. 2019; Vertullo et al. 2002).

Medical doctors may use pain as a marker and may consider analgesics for their athletes when there is pressure to return that athlete to sport (McAdams et al. 2016; Anderson et al. 2005). On‐field assessment pressure or the pressure experienced within team environments may cause doctors to limit the use of objective assessment criteria in assessments despite the knowledge of criteria needed for successful RTP (Aguilaniu et al. 2021; Testoni et al. 2013; Anderson et al. 2005; O'Neill LA, 2016). However, the practices that surround ethical issues experienced and RTP decisions of doctors were not clear in this review. Anderson (2009) highlighted the important educational role doctors assume when an athlete wants to take a course of action but whether doctors will support or limit the athlete is not understood (Anderson, 2009). The practices that surround those ethical issues is an area of research that could be explored and added to the ethical debate in literature (Burgess, 2011; Testoni et al. 2013; Anderson, 2009; Tekin et al. 2020; King et al. 2013).

### 5.2 Physiotherapists

The athlete arguably spends the most time with the physiotherapist during the rehabilitation process to build their functional and physical attributes to return safely to performance (Silva et al. 2011; Kaye et al. 2021; Taberner et al. 2020; Taberner et al. 2019). The frequency of clinical sessions with the physiotherapist may allow for more opportunity to collect strength and functional objective criteria (Fausett et al. 2022; Wörner et al. 2018). Due to the nature of the relationship between the athlete and the physiotherapist, physiotherapists are also inclined to assess the subjective or psychological state of the athlete (von Aesch et al. 2016; Kaye et al. 2021). However, physiotherapists have expressed concerns over assessing and implementing psychological interventions within their training and scope of practice (Kaye et al. 2021; Annear et al. 2019).

Physiotherapists have a preference for a milestone approach, monitoring strategies and the use of secondary prevention programmes (von Aesch et al. 2016; Greenberg et al. 2018; Nasser et al. 2021; Korakakis et al. 2021). However, there is a wide variety of RTP criteria that physiotherapists may consider; nevertheless, detail of the measuring tools used is lacking (von Aesch et al. 2016; Fausett et al. 2022; Mahnik et al. 2013; McRae et al. 2011; Nasser et al. 2021; Kaye et al. 2021; Fausett et al. 2019). More specialised physiotherapists are also better at using evidence‐based criteria (Aquino et al. 2021; van Melick et al. 2020). The time constraints and pressure experienced by physiotherapists during the recovery of athletes could account for a rushed and incomplete rehabilitation resulting in poor RTP outcomes (von Aesch et al. 2016; Greenberg et al. 2018; Nasser et al. 2021; Gauthier et al. 2023; Pulver et al. 2024; Fausett et al. 2019). Recent research has highlighted a gap between the frequency of rehabilitation and the time required for RTP (Fausett et al. 2022; Mahnik et al. 2013; McRae et al. 2011; Grindem et al. 2018; Fausett et al. 2019). Good use of referral, communication and SDM between the doctor and physiotherapist is important in RTP (von Aesch et al. 2016; Silva et al. 2011; Greenberg et al. 2018; Grindem et al. 2018). Sharing the responsibility between SMPs could assist at lessening the burden of assessing every component within a RTP criteria. Therefore, educating athletes and placing emphasis on efficient referral systems will enhance RTP outcomes (von Aesch et al. 2016; Greenberg et al. 2018; Nasser et al. 2021; Kaye et al. 2021; Grindem et al. 2018).

### 5.3 Rehabilitation Specialists

Rehabilitation specialists are involved within the final phase and in some cases known as ‘final phase rehabilitative specialists’ (Ellapen et al. 2017; South Africa). Recognised by the American Medical Association (AMA), athletic trainers prevent sport and exercise related injury (Ellapen et al. 2017; Prentice, 2013). Within a South African context, athletic trainers are comparable to the profession of biokinetics (Ellapen et al. 2017). From this review, rehabilitation specialists most frequently considered functional assessment and strength within RTP criteria (Di Trani Lobacz et al. 2016). Often working within a training facility with more space for rehabilitation and optimising performance, rehabilitation specialists have an opportunity to emphasise the importance of strength and function (Ellapen et al. 2017; Fournier, 2015; Di Trani Lobacz et al. 2016; Prentice, 2013). Spending more time with an athlete also allows rehabilitation specialists to flag psychological concerns (McVeigh et al. 2015). Therefore, rehabilitation specialists acknowledged the need for psychological training to assist assessment, intervention and ability to refer (McVeigh et al. 2015). The limited research of practitioners qualified in late stage rehabilitation and performance may be the missing link within the RTP decision‐making process (Dunlop et al. 2020; Yeomans et al. 2018).

### 5.4 MDTs

A finding from this review is that psychological readiness and sport‐specific testing is the most frequently considered MDT clearance criteria during RTP. This suggests that RTP in a MDT is more holistic in physically and mentally preparing athletes for RTP after an injury (Ardern et al. 2016; Ardern et al. 2016). Regarding roles or involvement within RTP, studies referring to the MDT consisted of combinations of various SMPs with doctors and physiotherapists making up the bulk of practitioners involved within these studies. The MDT studies were inclined to report outcomes in a monodisciplinary way (Rollo et al. 2021; Glazier, 2017; Piggott et al. 2019). That is, although SMPs work independently from each other and have different roles, research reports the collective outcome from the group (Rollo et al. 2021; Glazier, 2017; Piggott et al. 2019). Having a better understanding of roles and team function can improve collaboration and optimise attributes within the MDT (Shrier et al. 2014; Buckthorpe et al. 2019; Hess et al. 2022; Grindem et al. 2018). Collaboration will improve the athletes injury experience, compliance in rehabilitation and RTP performance (Shrier et al. 2014; Buckthorpe et al. 2019; Hess et al. 2022; Grindem et al. 2018).

Psychological readiness is a criteria that has been highlighted as a determinant for RTP success which correlates with the finding within MDT studies (Ardern et al. 2016; Lentz et al. 2018; Driver et al. 2017; Forsdyke et al. 2017). Psychological stressors and anxieties are common in injured athletes (Podlog et al. 2011; King et al. 2010). It is important for practitioners to recognise the need for hardiness‐training and stress management programs within rehabilitation and RTP (Podlog et al. 2011; King et al. 2010; Wadey et al. 2012; Ivarsson et al. 2017). Negative psychological responses to injury have been linked to unsuccessful RTP outcomes and being psychologically ready has been shown to be associated with preinjury performance (Ivarsson et al. 2017; Webster et al. 2019).

Sports medicine practitioners involved can be divided into categories of health and performance (Ellapen et al. 2017). Practitioners of the sports science team work more at improving the overall performance of the athlete whereas doctors, physiotherapists and rehabilitation specialists, such as biokineticists, work to rehabilitate the injury (Ellapen et al. 2017). With that said, RTP as a concept is evolving towards have performance as an outcome measure as opposed to functional ability after rehabilitation only (Draovitch et al. 2022; Buckthorpe et al. 2019; Taberner et al. 2019). Improving the RTP process will reduce the athlete's risk of reinjury (Shrier et al. 2014; Buckthorpe et al. 2019; Hess et al. 2022; Grindem et al. 2018). The impression that clinical rehabilitation and RTP is separate could account for the limited overlap between practitioners and the different perceptions in what is important for RTP (Buckthorpe et al. 2019; Balcı et al. 2020; Read et al. 2018; Yeomans et al. 2018). One reason could be due to the vague definition and starting time of RTP clearance (Shultz et al. 2013; Mazer et al. 2010; Boudier‐Reveret et al. 2011). Another could be the nature of injury and performance requirements of the athlete (Shrier, 2015). Sport medicine practitioners may regard clearing an athlete to RTP at different stages such as sport specific skill, training or competition (Shrier, 2015). Monitoring training load can have a protective effect and prevent the reoccurrence of an injury (Ardern et al. 2016a; Chen et al. 2022; Green et al. 2022; Gabbett, 2016). An athlete returning to play after injury has an almost doubled risk of injury in their first match in comparison to the in season injury rate (Bengtsson et al. 2020). Increased training sessions before the match reduces that risk first match back after injury (Bengtsson et al. 2020). Thus training loads should progress through each phase of recovery, rehabilitation and RTP (Green et al. 2022; Taberner et al. 2019).

Return to play decisions are ideally made using guidelines, protocols and criteria (Ardern et al. 2016; Matheson et al. 2011; Shrier, 2015; Bizzini et al. 2014). However, in this review, the use of published guidelines, RTP protocols or checklists of criteria were reported in a third of the studies (Beardmore et al. 2005; von Aesch et al. 2016; Aguilaniu et al. 2021; Dunlop et al. 2020; Erickson et al. 2014; Erickson et al. 2015; Petersen et al. 2013; Sherman et al. 2021; Ukogu et al. 2020; Morganti et al. 2001; Glattke et al. 2022; Pandey et al. 2022; Wörner et al. 2018; Read et al. 2018; Yeomans et al. 2018; Riendeau et al. 2015; Aquino et al. 2021; Greenberg et al. 2018; van Melick et al. 2020; Mendonca et al. 2020; Gauthier et al. 2023; Valente et al. 2023; McVeigh et al. 2015). Previous research has highlighted the need to prioritise a standardised approach or assessment to measure RTP outcomes, which strengthens the finding that clarity on practitioners roles, involvement, criteria and protocols is lacking (Ardern et al. 2016; Bizzini et al. 2014; Wikstrom et al. 2020). Perhaps a reason for the large variation of RTP decisions‐making practices is partially due to the volume of different criteria or difficult to use definitions and clinical guidelines (Greenhalgh et al. 2014). This highlights the need to prioritise an approach or assessment to measure RTP readiness for MSK‐I as a whole (Ardern et al. 2016a; Bizzini et al. 2014; Wikstrom et al. 2020).

## 6 Strength and Limitations

This review included all study designs and there was heterogeneity in the analysed studies. Although this can be viewed as a limitation, we felt that including both quantitative and qualitative (and mixed) study designs gave added depth to the findings of SMPs RTP practices. Thematic analysis was used to interpret and generalise the results (Ma et al. 2020; Griffin et al. 2021; Braun et al. 2006). Again, although this approach may be viewed as a limitation, synthesising the information on SMPs decision‐making practices during RTP using thematic analysis allows the reader to get an understanding on how diverse this area of research is. Grouping themes and connecting different areas of research provides a platform on which to navigate future research (Griffin et al. 2021; Braun et al. 2006). It was worth noting that the majority of the studies included in the review were published over the last decade highlighting the growing interest in RTP, whereas older studies provided further context to the findings.

All the studies within the review were cross‐sectional in design. The studies consisted of surveys, questionnaires and interviews based on expert opinion (Obremskey et al. 2005; Burns et al. 2011). Most of the studies used self‐constructed questionnaires and surveys developed from focus group discussions, collaboration with colleagues and outcomes reported in literature (Ma et al. 2020). Many of the surveys were peer reviewed or underwent pilot review to ensure validity or reliability (Braithwaite et al. 2003). Survey studies on expert opinions are important in evidence‐based medicine as they provide a glimpse into an area of concern within a defined population (Ma et al. 2020). Also, cross‐sectional studies are effective at determining prevalence and identifying associations for future work (Mann 2003). Cross‐sectional studies are generally quick to administer and can result in multiple outcomes, providing a snapshot of the current decision‐making practice of practitioners (Mann 2003). However, explanation for those decisions are difficult to determine using predetermined answers within surveys, limiting the opportunity for more details (Mann 2003).

### 6.1 Recommendations for Future Research

The present review highlighted that without clear understanding of RTP practice including the use of criteria and factors that influence decision making, consistency in RTP can be difficult. Return to play decisions become complicated due to the many factors that SMPs need to consider before deciding that RTP after MSK‐I is safe. Sports medicine practitioners need to have a good understanding of their athlete, the type of injury and the injury prevention markers that need to be achieved. However, the variability within practices emphasise the need for simple implementation tools especially when working in more complex environments (Green et al. 2022). With the knee being the most researched anatomical area, SMPs use of RTP protocols and clearance may be skewed. Future research needs to define RTP clearance, validate the different criteria, and standardise RTP protocols at the various stages of the RTP process are needed. Highlighting the need to make all findings applicable to all conditions.

The RTP process and decision‐making criteria for all injuries, sporting codes and athletic populations were reported. Although different injuries, sports and athlete populations have unique complexities, this review provides an overview of RTP practice of MSK‐I in sport. However, future research could focus on individual sporting codes, specific injuries and specific athlete populations.

For an evidence‐based RTP clearance criteria or practice to be enforced, this review suggests better collaboration between SMPs within their roles in RTP. Future research should aim to understand SMPs roles within the RTP process. Understanding the overlap of roles may improve interpretation of RTP assessments, improve the use of referral systems and improve collaboration between SMPs. Making use of referral patterns may also improve an athletes compliance for a complete rehabilitation into RTP.

## 7 Conclusion

This is the first review aimed to provide an understanding of SMPs roles and decision‐making practice when returning athletes to play after MSK‐I. The findings of this review indicate that medical doctors, physiotherapists, rehabilitation specialists and a variety of additional SMPs of the MDT are involved with clearing an athlete to RTP. It is unclear from the MDT studies how individual MDT members navigate and contribute towards RTP decisions. However, the separate SMP groups give more insight into what each profession may consider important within RTP clearance. Many of studies addressed the knee and medical doctors were the most studied population in RTP. The rehabilitation specialist was the least studied population. Shared decision‐making is preferred amongst SMPs; however, the different roles decision‐makers have within RTP is not clear.

Although SMPs consider similar criteria within the RTP process, within practitioner’s group criteria is weighted differently. Return to play time is considered by majority of SMPs; however, variations in timelines could be due to additional factors that influence the SMPs' decisions. Factors include athlete pressure, injury characteristics and practitioner experience and qualifications. Injury or postoperative recovery timelines were the most considered criteria especially within medical doctor studies. Although most SMPs consider RTP time, time to RTP may vary. Functional assessment, strength and psychological readiness also appear to be important considerations. Multidisciplinary team studies consider psychological readiness and sport specific testing criteria most in RTP. A variety of SMPs are involved within MDT studies. Using a team of different SMPs, a more comprehensive RTP criteria can be considered. However within MDT studies there is a lack of clarity on each SMP roles and contribution towards RTP. Information on assessment tools and methods used in criteria, such as sport specific tests, and RTP protocols is limited.

The present review highlighted that without collaboration and practical tools with clear strategies, SMPs may struggle to be consistent in RTP practice. This review can assist MDT collaboration, future development of a reference tool, definition standardisation and RTP clearance criteria to support SMPs when making difficult RTP decisions.

## Ethics Statement

This review was approved by the University of Cape Town, Faculty of Health Sciences Human Research Ethics Committee (HREC/REF 207/2022).

## Conflicts of Interest

SH is an Associate Editor for EJSS and Social Media Editor.

## Supporting information


Supporting Information S1
