# Supplementary material for: Ready or Not; A Narrative Synthesis of Sports Medicine Practitioners' Practices During Return to Play in the Management of Musculoskeletal Injuries
Source: Eur J Sport Sci. 2025 Oct 25;25(11):e70071. doi: 10.1002/ejsc.70071 (PMC12553449; doi:10.1002/ejsc.70071)
Supplement: Supplementary file 1 — Supporting Information S1 [file EJSC-25-e70071-s001.docx]

Supplementary Table 1: PRISMA Checklist

| **Section and Topic** | **Item #** | **Checklist item** | **Location where item is reported** |
| --- | --- | --- | --- |
| **TITLE** | | |  |
| Title | 1 | Identify the report as a systematic review. | Pg 1 |
| **ABSTRACT** | | |  |
| Abstract | 2 | See the PRISMA 2020 for Abstracts checklist. | Pg 1 |
| **INTRODUCTION** | | |  |
| Rationale | 3 | Describe the rationale for the review in the context of existing knowledge. | Pg 4 |
| Objectives | 4 | Provide an explicit statement of the objective(s) or question(s) the review addresses. | Pg 5 |
| **METHODS** | | |  |
| Eligibility criteria | 5 | Specify the inclusion and exclusion criteria for the review and how studies were grouped for the syntheses. | Pg 5-6 |
| Information sources | 6 | Specify all databases, registers, websites, organisations, reference lists and other sources searched or consulted to identify studies. Specify the date when each source was last searched or consulted. | Pg 5 |
| Search strategy | 7 | Present the full search strategies for all databases, registers and websites, including any filters and limits used. | Pg 5 + supplementary Table 3 |
| Selection process | 8 | Specify the methods used to decide whether a study met the inclusion criteria of the review, including how many reviewers screened each record and each report retrieved, whether they worked independently, and if applicable, details of automation tools used in the process. | Pg 6 |
| Data collection process | 9 | Specify the methods used to collect data from reports, including how many reviewers collected data from each report, whether they worked independently, any processes for obtaining or confirming data from study investigators, and if applicable, details of automation tools used in the process. | Pg 6-8 |
| Data items | 10a | List and define all outcomes for which data were sought. Specify whether all results that were compatible with each outcome domain in each study were sought (e.g. for all measures, time points, analyses), and if not, the methods used to decide which results to collect. | Pg 8 |
|  | 10b | List and define all other variables for which data were sought (e.g. participant and intervention characteristics, funding sources). Describe any assumptions made about any missing or unclear information. | Pg 8 |
| Study risk of bias assessment | 11 | Specify the methods used to assess risk of bias in the included studies, including details of the tool(s) used, how many reviewers assessed each study and whether they worked independently, and if applicable, details of automation tools used in the process. | Pg 8 |
| Effect measures | 12 | Specify for each outcome the effect measure(s) (e.g. risk ratio, mean difference) used in the synthesis or presentation of results. | Pg 8-10 |
| Synthesis methods | 13a | Describe the processes used to decide which studies were eligible for each synthesis (e.g. tabulating the study intervention characteristics and comparing against the planned groups for each synthesis (item #5)). | Pg 8-10 |
|  | 13b | Describe any methods required to prepare the data for presentation or synthesis, such as handling of missing summary statistics, or data conversions. | Pg 8-10 |
|  | 13c | Describe any methods used to tabulate or visually display results of individual studies and syntheses. | Pg 8-10 |
|  | 13d | Describe any methods used to synthesize results and provide a rationale for the choice(s). If meta-analysis was performed, describe the model(s), method(s) to identify the presence and extent of statistical heterogeneity, and software package(s) used. | Pg 5, 8-10 |
|  | 13e | Describe any methods used to explore possible causes of heterogeneity among study results (e.g. subgroup analysis, meta-regression). | Pg 10-11 |
|  | 13f | Describe any sensitivity analyses conducted to assess robustness of the synthesized results. | NA |
| Reporting bias assessment | 14 | Describe any methods used to assess risk of bias due to missing results in a synthesis (arising from reporting biases). | Pg 8-10 |
| Certainty assessment | 15 | Describe any methods used to assess certainty (or confidence) in the body of evidence for an outcome. | Pg 8-10 |
| **RESULTS** | | |  |
| Study selection | 16a | Describe the results of the search and selection process, from the number of records identified in the search to the number of studies included in the review, ideally using a flow diagram. | Pg 7 |
|  | 16b | Cite studies that might appear to meet the inclusion criteria, but which were excluded, and explain why they were excluded. | Pg 7 |
| Study characteristics | 17 | Cite each included study and present its characteristics. | Pg 10-11 |
| Risk of bias in studies | 18 | Present assessments of risk of bias for each included study. | Pg 11 |
| Results of individual studies | 19 | For all outcomes, present, for each study: (a) summary statistics for each group (where appropriate) and (b) an effect estimate and its precision (e.g. confidence/credible interval), ideally using structured tables or plots. | Pg 11-17 |
| Results of syntheses | 20a | For each synthesis, briefly summarise the characteristics and risk of bias among contributing studies. | Pg 11 |
|  | 20b | Present results of all statistical syntheses conducted. If meta-analysis was done, present for each the summary estimate and its precision (e.g. confidence/credible interval) and measures of statistical heterogeneity. If comparing groups, describe the direction of the effect. | Pg 11-17 |
|  | 20c | Present results of all investigations of possible causes of heterogeneity among study results. | Pg 11-17 |
|  | 20d | Present results of all sensitivity analyses conducted to assess the robustness of the synthesized results. | NA |
| Reporting biases | 21 | Present assessments of risk of bias due to missing results (arising from reporting biases) for each synthesis assessed. | Pg 11 |
| Certainty of evidence | 22 | Present assessments of certainty (or confidence) in the body of evidence for each outcome assessed. | Pg 11 |
| **DISCUSSION** | | |  |
| Discussion | 23a | Provide a general interpretation of the results in the context of other evidence. | Pg 18-22 |
|  | 23b | Discuss any limitations of the evidence included in the review. | Pg 23 |
|  | 23c | Discuss any limitations of the review processes used. | Pg 23 |
|  | 23d | Discuss implications of the results for practice, policy, and future research. | Pg 23 |
| **OTHER INFORMATION** | | |  |
| Registration and protocol | 24a | Provide registration information for the review, including register name and registration number, or state that the review was not registered. | Pg 5 |
|  | 24b | Indicate where the review protocol can be accessed, or state that a protocol was not prepared. | Pg 5 |
|  | 24c | Describe and explain any amendments to information provided at registration or in the protocol. | Pg 5 |
| Support | 25 | Describe sources of financial or non-financial support for the review, and the role of the funders or sponsors in the review. | Pg 25 |
| Competing interests | 26 | Declare any competing interests of review authors. | Pg 25 |
| Availability of data, code and other materials | 27 | Report which of the following are publicly available and where they can be found: template data collection forms; data extracted from included studies; data used for all analyses; analytic code; any other materials used in the review. | Supplementary Material |

*From:*  Page MJ, McKenzie JE, Bossuyt PM, Boutron I, Hoffmann TC, Mulrow CD, et al. The PRISMA 2020 statement: an updated guideline for reporting systematic reviews. BMJ 2021;372:n71. doi: 10.1136/bmj.n71. This work is licensed under CC BY 4.0. To view a copy of this license, visit <https://creativecommons.org/licenses/by/4.0/>

Supplementary Table 2: PRISMA Abstract Checklist

| **Section and Topic** | **Item #** | **Checklist item** | **Reported (Yes/No)** |
| --- | --- | --- | --- |
| **TITLE** | | |  |
| Title | 1 | Identify the report as a systematic review. | yes |
| **BACKGROUND** | | |  |
| Objectives | 2 | Provide an explicit statement of the main objective(s) or question(s) the review addresses. | yes |
| **METHODS** | | |  |
| Eligibility criteria | 3 | Specify the inclusion and exclusion criteria for the review. | yes |
| Information sources | 4 | Specify the information sources (e.g. databases, registers) used to identify studies and the date when each was last searched. | yes |
| Risk of bias | 5 | Specify the methods used to assess risk of bias in the included studies. | yes |
| Synthesis of results | 6 | Specify the methods used to present and synthesise results. | yes |
| **RESULTS** | | |  |
| Included studies | 7 | Give the total number of included studies and participants and summarise relevant characteristics of studies. | yes |
| Synthesis of results | 8 | Present results for main outcomes, preferably indicating the number of included studies and participants for each. If meta-analysis was done, report the summary estimate and confidence/credible interval. If comparing groups, indicate the direction of the effect (i.e. which group is favoured). | yes |
| **DISCUSSION** | | |  |
| Limitations of evidence | 9 | Provide a brief summary of the limitations of the evidence included in the review (e.g. study risk of bias, inconsistency and imprecision). | yes |
| Interpretation | 10 | Provide a general interpretation of the results and important implications. | yes |
| **OTHER** | | |  |
| Funding | 11 | Specify the primary source of funding for the review. | NA |
| Registration | 12 | Provide the register name and registration number. | yes |

Supplementary Table 3: Search Strategy Examples

| An example the search key in PubMed: ((((((("return to play"[Title/Abstract]) OR ("return to sport"[Title/Abstract])) OR ("return to performance"[Title/Abstract])) OR ("return to participation"[Title/Abstract])) OR ("return to function"[Title/Abstract])) OR ("return to training"[Title/Abstract])) OR ("return to competition"[Title/Abstract])) AND ((((((("knowledge"[Title/Abstract]) OR ("attitude*"[Title/Abstract])) OR ("behaviour*"[Title/Abstract])) OR ("behavior*"[Title/Abstract])) OR ("practic*"[Title/Abstract])) OR ("perception*"[Title/Abstract])) OR ("opinion*"[Title/Abstract]))  An example of the search key in Scopus: ( ( TITLE-ABS-KEY ( "return to play" ) )  OR  ( TITLE-ABS-KEY ( "return to sport" ) )  OR  ( TITLE-ABS-KEY ( "return to performance" ) )  OR  ( TITLE-ABS-KEY ( "return to participation" ) )  OR  ( TITLE-ABS-KEY ( "return to function" ) )  OR  ( TITLE-ABS-KEY ( "return to training" ) )  OR  ( TITLE-ABS-KEY ( "return to competition" ) ) )  AND  ( ( TITLE-ABS-KEY ( "opinion*" ) )  OR  ( TITLE-ABS-KEY ( "knowledge*" ) )  OR  ( TITLE-ABS-KEY ( "behaviour*" ) )  OR  ( TITLE-ABS-KEY ( "behavior*" ) )  OR  ( TITLE-ABS-KEY ( "practic*" ) )  OR  ( TITLE-ABS-KEY ( "perception*" ) )  OR  ( TITLE-ABS-KEY ( "attitude*" ) ) ) |
| --- |

##

Supplementary Table 4: Brief overview of studies included in the systematic review

| **Author** | **Year** | **Journal** | **Title** | **Source** | **Study design** | **Response Rate % (n= sample size)** | **JBI appraisal tool** | **AXIS appraisal tool /20** |
| --- | --- | --- | --- | --- | --- | --- | --- | --- |
| MEDICAL DOCTORS | | | | | | | | |
| RTP PRACTICE (e.g. injury scenarios, ethics, general practice) | | | | | | | | |
| Anderson, L. C.  Gerrard, D. F. | 2005 | Clinical Ethics | Ethical issues concerning New Zealand sports doctors | BMJ | Questionnaire using survey closed and open ended questions | 40%  (n=18) | - | 16 |
| Hobusch, G. M., Keusch, F., Tsuchiya, H., Joyce, M., Windhager, R. | 2020 | Journal of Clinical Medicine | What Opinions Do Tumor Reconstructive Surgeons Have about Sports Activity after Megaprosthetic Replacement in Hip and Knee? Results of the MoReSports Expert Opinion Online Survey | MDPI | Cross sectional – web survey | 12.3-21.9% (n=76) |  | 16 |
| Schrock, J. B., Carver, T. J., Kraeutler, M. J., McCarty, E. C. | 2018 | Sports Health | Evolving Treatment Patterns of NFL Players by Orthopaedic Team Physicians Over the Past Decade, 2008-2016 | Sage | Cross sectional - Descriptive epidemiology study | 97% (n=31) + 97% (n=29) | - | 16 |
| SPINE | | | | | | | | |
| Abla, A. A., Maroon, J. C., Lochhead, R., Sonntag, V. K., Maroon, A., Field, M. | 2011 | J Neurosurg: Spine | Return to golf after spine surgery | Neuroscience publications | Cross sectional – emailed survey | 52.3% (n=523) | - | 14 |
| Backer, H. C.  Johnson, M. A.  Hanlon, J.  Chan, P.  Turner, P.  Cunningham, J. | 2024 | European Spine Journal | Return to sports following discectomy: does a consensus exist? | Springer | Cross-sectional | 18.5% (n=31) |  | 15 |
| France, J.C., Karsy, M., Harrop, J.S., Dailey, A.T. | 2016 | Global Spine Journal | RTP after Cervical Spine Injuries: A consensus of opinion | Thieme | Cross sectional - survey | 100% (n=25) | - | 12 |
| Ho, D., Du, J. Y., Erkilinc, M., Glotzbecker, M. P., Mistovich, R. J. | 2021 | Journal of Paediatric Orthopaedics | Getting Them Back in the Game: When Can Athletes With Adolescent Idiopathic Scoliosis Safely Return to Sports? A Mixed-effects Study of the Pediatric Orthopaedic Association of North America | Wolters Kluwer | Cross sectional study - questionnaire | 11.36% (n=170) | - | 17 |
| Morganti, C., Sweeney, C.A., Albanese, S.A., Burak, C., Hosea, T., Connolly, P.J. | 2001 | Spine | RTP After Cervical Spine Injury | Lippincott Williams & Wilkins, Inc. | Cross sectional study - questionnaire | 32.7% (n=113) | - | 13 |
| Ukogu, C., Bienstock, D., Ferrer, C., Zubizarreta, N., McAnany S., Chaudhary, S. B., Iatridis, J. C., Hecht, A. C. | 2020 | Clinical Spine Surgery | Physician Decision-making in RTP after Cervical Spine Injury | Wolters Kluwer | Cross sectional study - Prospective survey study | 78.48% (n=62) | - | 19 |
| THORAX | | | | | | | | |
| McAdams, T. R.  Deimel, J. F.  Ferguson, J.  Beamer, B. S.  Beaulieu, C. F. | 2016 | The Orthopaedic Journal of Sports Medicine | Chondral Rib Fractures in Professional American Football | The Author(s) | Case series with cross sectional survey component | 71.8% (n=23) | - | 6 |
| SHOULDER | | | | | | | | |
| Golant, A., Christoforou, D., Zuckerman, J. D., Kwon, Y. W. | 2012 | Journal of shoulder and elbow surgery | Return to sports after shoulder arthroplasty: a survey of surgeons' preferences | Elsevier | Cross sectional stud – web based survey | 30.3% (n=94) | - | 16 |
| Moore, D. M., Hurley, E.T., Mullett, H. | 2020 | The Surgeon: journal of the Royal Colleges of Surgeons of Edinburgh and Ireland | Current practices in the management of anterior glenohumeral instability in rugby union players | Elsevier | Cross sectional  Online survey administered via email and annual meeting | -  (n=98) | - | 15 |
| Sharareh, B., Edwards, T. B., Shah, A., Shybut, T. | 2021 | Journal of Shoulder and Elbow Surgery | Variation in technique and postoperative management of the Latarjet procedure among orthopedic surgeons | Elsevier | Cross sectional study | -  (n=242) | - | 17 |
| ELBOW | | | | | | | | |
| Sambare, N. D.  Chalmers, P. N.  Camp, C. L.  Bowman, E. N.  Erickson, B. J.  Sciascia, A.  Freehill, M. T.  Smith, M. V. | 2024 | Journal of Shoulder and Elbow Surgery Reviews, Reports, and Techniques | High variability among surgeons in evaluation, treatment, and rehabilitation of medial ulnar collateral ligament injuries | Elsevier | Cross-sectional | 77% (n=24) |  | 19 |
| HAND AND WRIST INJURIES | | | | | | | | |
| Dy, C. J., Khmelnitskaya, E., Hearns, K. A., Carlson, M. G. | 2013 | Orthopedics | Opinions Regarding the Management of Hand and Wrist Injuries in Elite Athletes | Healio | Cross sectional study | 47.44%  (n=37) | - | 14 |
| HIP/GROIN/PELVIC INJURIES | | | | | | | | |
| Domb, B.G., Stake, C.E.  Finch, N.A., Cramer, L. | 2014 | Orthopaedics | Return to Sport After Hip Arthroscopy: Aggregate Recommendations From High-volume Hip Arthroscopy Centers | Healio | Cross sectional – survey | 100% (n=27) | - | 11 |
| Vu-Han, T., Hardt, S., Ascherl, R., Gwinner, C., Perka, C. | 2021 | Archives of Orthopaedic and Trauma Surgery | Recommendations for return to sports after total hip arthroplasty are becoming less restrictive as implants improve | Springer | Cross sectional – emailed survey | 33% (n=99) | - | 17 |
| KNEE INJURIES | | | | | | | | |
| Arliani, G.G., Pereira, V.L., Leao, R.G., Lara, P.S., Ejnisman, B., Cohen, M. | 2019 | Rev Bras Ortop | Treatment of Anterior Cruciate Ligament Injuries in Professional Soccer Players by Orthopedic Surgeons | Thieme | Cross sectional study | 100% (n=61) | - | 17 |
| Bakowski, P., Bakowski-Zywicka, K., Pointek, T. | 2020 | BMC Musculoskeletal Disorders | Clinical practice and postoperative  rehabilitation after knee arthroscopy vary according to surgeons’ expertise: a survey among polish arthroscopy society members | BMC | Cross sectional survey study | 100% (n=205) | - | 17 |
| Betsch, M.,  Darwich, A.,  Chang, J.,  Whelan, D.,  Ogilvie-Harris, D.,  Chahal, J.,  Theodoropoulos, J. | 2022 | Arthroscopy, Sports Medicine, and Rehabilitation | Wide Variability in Return-to-Sport Criteria used by Team Physicians After Anterior Cruciate Ligament Reconstruction in Elite Athletes - A Qualitative Study | Elsevier | Semi-structured interviews - Qualitative | -  (n=18) | JBI Qualitative 9/10 | 16 |
| Coskunsu, D., Bayrakci, Tunay, V., Akgun, I. | 2010 | Acta Orthopaedica et Traumalogica Turcica | Current trends in reconstruction surgery and rehabilitation of  anterior cruciate ligament in Turkey | DergiPark Academik | Cross sectional study - questionnaire | 70.9% (n=39) | - | 13 |
| Ebert, J. R., Webster, K.E., Edwards, P. K., Joss, B. K., D’Alessandro, P. , Janes, G., Annear, P. | 2020 | Journal of Sport Rehabilitation | Current Perspectives of the Australian Knee Society on Rehabilitation and Return to Sport After Anterior Cruciate Ligament Reconstruction | Human Kinetics | Cross sectional survey study | 85% (n=73) | - | 14 |
| Erickson, B. J., Harris, J. D., Fillingham, Y. A., Cvetanovich, G. L., Bush-Joseph, C., Cole, B. J., Bach, B. R., Verma, N.N. | 2015 | The American Journal of Orthopedics | Orthopedic Practice Patterns Relating to Anterior Cruciate Ligament Reconstruction in Elite Athletes | Research Gate | Cross sectional study | 50% (n=47) | - | 18 |
| Erickson B. J., Harris, J. D., Fillingham, Y. A., Frank, R. M., Bush, Joseph, C. A., Bach Jr, B. R., Cole, B. J., Verma, N. N. | 2014 | Arthroscopy: The Journal of Arthroscopic and Related Surgery | Anterior Cruciate Ligament Reconstruction Practice Patterns by NFL and NCAA Football Team Physicians | Elsevier | Cross sectional study - survey | 51% (n=137) | - | 16 |
| Farber, J.  Harris, J.D.  Kolstad, K.  McCulloch, P.C. | 2014 | The Orthopaedic Journal of Sports Medicine | Treatment of Anterior Cruciate Ligament Injuries by Major League Soccer Team Physicians | Sage publishers | Cross sectional study | 100% (n=22) | - | 18 |
| Feller, J. A.  Cooper, R.  Webster, K. E. | 2002 | The Knee | Current Australian trends in rehabilitation following anterior cruciate ligament reconstruction | Elsevier | Cross sectional study - questionnaire | 95% (n=36) | - | 13 |
| Glattke, K.E.,  Tummala, S.V., Goldberg, B., Menzer, H., Chhabra, A. | 2022 | Arthroscopy | There Is Substantial Variation in Rehabilitation Protocols Following Anterior Cruciate Ligament Reconstruction: A Survey of 46 American Orthopaedic Surgeons | Elsevier | Cross-sectional – online survey | -  (n=46) | - | 15 |
| Grassi, A.  Vascellari, A.  Combi, A.  Tomaello, L.  Canatas, G.L.  Zaffagnini, S. | 2016 | European Journal of Orthopaedic Surgical Traumatology | Return to sport after ACL reconstruction: a survey  between the Italian Society of Knee, Arthroscopy, Sport, Cartilage and Orthopaedic Technologies (SIGASCOT) members | Springer | Cross sectional study – survey | 16% (n=123) | - | 17 |
| Mahnik, A.  Mahnik, S.  Dimnjakovic, D.  Curic, S.  Smoljanovic, T.  Bojanic, I. | 2013 | World Journal of Orthopaedics | Current practice variations in the management of anterior cruciate ligament injuries in Croatia | Baishideng | Cross sectional study – emailed survey | 20.63% (n=39) | - | 14 |
| Marshall, N.E.  Keller, R.A  Dines, J.  Bush-Joseph, C.  Limpisvasti, O. | 2019 | Musculoskeletal Surgery | Current practice: postoperative and RTP trends after ACL  reconstruction by fellowship‑trained sports surgeons | Springer | Cross sectional study | 32% (n=143) | - | 16 |
| McRae, S. M.  Chahal, J.  Leiter, J. R.  Marx, R. G.  MacDonald, P. B. | 2011 | Clinical Journal of Sports Medicine | Survey Study of Members of the Canadian Orthopaedic Association on the Natural History and Treatment of Anterior Cruciate Ligament Injury | Lippincott Williams & Wilkins, Inc. | Online survey cross sectional study | 49% (n=283) | - | 19 |
| Pandey, V.  Madi, S.  Thonse, C.  Joseph, C.  Rajan, D.  Varughese, J.  Thilak, J.  Jayaprasad, P. S.  Acharya, K.  Ramamurthy, K. G.  Reddy, R.  Amravathi, R.  Rao, S.  Gangavarapu, S.  Srinivas, M.  Jose, S.  Sundararjan, S. R. | 2022 | Indian Journal of Orthopaedics | Trends in Primary Anatomical Single-Bundle Anterior Cruciate Ligament Reconstruction Practice in Adult Patients Prevalent Among Arthroscopy Surgeons of Six Southern States of India | Springer | Cross sectional survey study | 55% (n=324) | - | 17 |
| Petersen, W.  Zantop, T. | 2013 | Arthroscopy and Sports Medicine | RTP following ACL reconstruction: survey among experienced arthroscopic surgeons (AGA instructors) | Springer | Questionnaire study – cross sectional | 80.6% (n=221) | - | 16 |
| Sherman, S. L.  Calcei, J.  Ray, T.  Magnussen, R. A.  Musahl, V.  Kaeding, C. C.  Clatworthy, M.  Bergfeld, J. A.  Arnold, M. P. | 2021 | Journal of ISAKOS | ACL Study Group presents the global trends in ACL reconstruction: Biennial survey of the ACL Study Group | BMJ | Survey | 100% (n=140) | - | 15 |
| Thaler, M.  Khosravi, I.  Putzer, D.  Michael T.  Hirschmann, M. T.  Kort, N.  Tandogan, R. N. Liebensteiner, M. | 2021 | European Society of Sports Traumatology, Knee Surgery, Arthroscopy (ESSKA) | Twenty‑one sports activities are recommended by the European Knee Associates (EKA) six months after total knee arthroplasty | Springer | Cross-sectional study - Survey | 57.7% (n=120) | - | 16 |
| Vascellari, A.  Grassi, A.  Combi, A.  Tomaello, L.  Canata, G. L.  Zaffagnini, S.  Sigascot Sports Committee | 2017 | Knee Surgery Sports Traumatology Arthroscopy | Web-based survey results: surgeon practice patterns in Italy regarding anterior cruciate ligament reconstruction and rehabilitation | Springer | Web-based survey | 17% (n=131) | - | 16 |
| **Ankle and foot injuries** | | | | | | | | |
| Aguilaniu, A.  Croisier, J. L.  Schwartz, C.  Dardenne, N.  D'Hooghe, P.  Martens, G.  Collin, R.  Kaux, J. F. | 2021 | Foot and Ankle Surgery | Current practice for safe return-to-play after lateral ankle sprain: A survey among French-speaking physicians | Elsevier | Online survey | 21.8% (n=109) | - | 16 |
| Beck, J.J., Carpenter, C.M., West, N., Sabatino, M.J., Ellis, H.B. | 2022 | The Orthopaedic Journal of Sports Medicine | Treatment Variability and Complications Associated with Pediatric Lateral Ankle Injuries: A POSNA Quality, Safety, and Value Initiative Survey | Sage publishers | Cross sectional study | 16.4% (n=229) | - | 15 |
| Dams, O.C.  Van den Akker-Scheek, I.  Diercks, R.L.  Wendt, K.W.  Zwerver, J.  Reininga, I.H.F | 2019 | Knee Surgery, Sports Traumatology, Arthroscopy | Surveying the management of Achilles tendon ruptures  in the Netherlands: lack of consensus and need for treatment  guidelines | Springer | Cross sectional study | 45.5% (n=91) | - | 18 |
| Vertullo, C.  Nunley, J. A. | 2002 | Ankle and Foot International | Participation in Sports after Arthrodesis of the Foot or Ankle | American Foot & Ankle Society, Inc. | Questionnaire | 20.6% (n=103) | - | 15 |
| Yokoe, T.  Tajima, T.  Yamaguchi, N.  Morita, Y.  Chosa, E. | 2021 | BMC Musculoskeletal disorders | The current clinical practice of general orthopaedic surgeons in the treatment of lateral ankle sprain: a questionnaire survey in Miyazaki, Japan | Springer Nature | Questionnaire | 82.7% (n=129) | - | 17 |
| **Physiotherapists** | | | | | | | | |
| **Generalised RTP** | | | | | | | | |
| Silva, Anderson A  Bittencourt, Natália FN  Mendonça, Luciana M  Tirado, Marcella G  Sampaio, Rosana F  Fonseca, Sérgio T | 2011 | Brazilian Journal of Physical Therapy | Analysis of the profile, areas of action and abilities of Brazilian sports physical therapists working with soccer and volleyball | Scielo | Cross sectional survey | -  (n=49) | - | 14 |
| **Shoulder injuries** | | | | | | | | |
| Brindisino, Fabrizio  De Santis, Andrea  Rossettini, Giacomo  Pellicciari, Leonardo  Filipponi, Marco  Rollo, Giuseppe  Gibson, Jo | 2021 | Disability & Rehabilitation | Post-surgery rehabilitation following rotator cuff repair. A survey of current (2020) Italian clinical practice | Taylor & Francis | Web-based observational cross-sectional study | -  (n= 1160) | - | 16 |
| Brindisino, F.  Lorusso, M.  Usai, M.  Pellicciari, L.  Marruganti, S.  Salomon, M. | 2023 | Archives of Physiotherapy | Rehabilitation following shoulder arthroplasty: a survey of current clinical practice patterns of Italian physiotherapists | BMC | Cross-sectional observation | -  (n=607) |  | 19 |
| Gauthier, M. L.  Unverzagt, C. A.  Mendonça, L. M.  Seitz, A. L. | 2023 | International Journal of Sports Physical Therapy | Missing the forest for the trees: a lack of upper extremity physical performance testing in sports physical therapy |  | Cross-sectional | 2.3%  (n=498) |  | 20 |
| **Hamstring injuries** | | | | | | | | |
| Nasser, A. M.  Pizzari, T.  Grimaldi, A.  Vicenzino, B.  Rio, E.  Semciw, A. I. | 2021 | Physical Therapy in Sport | Proximal hamstring tendinopathy; expert physiotherapists’  perspectives on diagnosis, management and prevention | Elsevier | Semi-structured interviews – qualitative study | 92.8%  (n=13) | Qualitative JBI tool | 17 |
| Valente, H. G.  Oliveira, R. R.  Baroni, B. M. | 2023 | Physical Therapy in Sport | How are hamstring strain injuries managed in elite men's football clubs? a survey with 62 Brazilian physical therapists | Elsevier | Cross-sectional | 65% (n=62) |  | 20 |
| **Knee injuries** | | | | | | | | |
| Alshehri, Y. S.  Aljohani, M. M. A.  Alzahrani, H.  Alzhrani, M.  Alkhathami, K. M.  Alshahrani, A.  Khaled, O. A. | 2024 | Journal of Sport Rehabilitation | Current rehabilitation practices and return to sports criteria after anterior cruciate ligament reconstruction: a survey of physical therapists in Saudi Arabia | Human Kinetics | Cross-sectional | 88% (n=177) |  | 18 |
| Aquino, C.F.  Ocarino, J. M.  Cardosos, V. A.  Resende, R. A.  Souza, T. R.  Rabelo, L. M.  Fonseca, S. T. | 2021 | Brazilian Journal of Physical Therapy | Current clinical practice and return-to-sport criteria after anterior cruciate ligament reconstruction: a survey of Brazilian physical therapists | Elsevier | Cross sectional study – electronic survey | -  (n= 439) | 8/8 | 16 |
| Fausett, W. A.  Reid, D. A.  Larmer, P. J. | 2022 | Physical Therapy in Sport | Current perspectives of New Zealand physiotherapists on rehabilitation and return to sport following anterior cruciate ligament reconstruction: A survey | Elsevier | Online cross sectional survey | 29.3% (n=318) | - | 15 |
| Greenberg, E. M.  Greenberg, E.T.  Albaugh, J.  Storey, E.  Ganley, T.J. | 2018 | Journal of Orthopaedic & Sports Physical Therapy | Rehabilitation Practice Patterns Following Anterior Cruciate Ligament Reconstruction: A Survey of Physical Therapists | JOSPT | Cross-sectional study | -  (n= 1074) |  | 16 |
| Kaye, J. A.  Spence, D.  Alexanders, J. | 2021 | Physiotherapy Theory and Practice | Using a Biopsychosocial Approach within ACL Rehabilitation: An Exploration of Student Physiotherapists` Perceptions and Experiences | Taylor & Francis Group | Qualitative study | -  (n=10) | JBI Qualitative tool 10/10 | 19 |
| Korakakis, V.  Kotsifaki, A.  Korakaki, A.  Karanasios, S.  Whiteley, R. | 2021 | Physical Therapy in Sport | Current perspectives and clinical practice of physiotherapists on assessment, rehabilitation, and return to sport criteria after anterior cruciate ligament injury and reconstruction. An online survey of 538 physiotherapists | Elsevier | cross-sectional design online survey | 7% (n=538) |  | 17 |
| Mendonca, L. D. M.  Bittencourt, N. F. N.  Alves, L. E. M.  Resende, R. A.  Serrão, F. V. | 2020 | Brazilian Journal of Physical Therapy | Interventions used for Rehabilitation and Prevention of Patellar Tendinopathy in athletes: a survey of Brazilian Sports Physical Therapists | Elsevier | Cross sectional study | -  (n= 121) | 5/8 | 17 |
| Pulver, M.  Hilfiker, R.  Bizzini, M.  Mathieu, N.  Meyer, S.  Allet, L. | 2024 | Physical Therapy in Sport | Clinical practice and barriers among Swiss physiotherapists treating patients with anterior cruciate ligament reconstruction: a survey of pre-operative rehabilitation to return to sport | Elsevier | Cross-sectional | -  (n=247) |  | **17** |
| Tondelli, E.  Feroldi, A.  García, F.  Meza, F.  Dingenen, B. | 2024 | Physical Therapy in Sport | Rehabilitation and return-to-sport after anterior cruciate ligament injury and reconstruction: exploring physical therapists' approaches in Argentina | Elsevier | Cross-sectional | -  (n=619) |  | 19 |
| van Melick, N.  Hoogeboom, T. J.  Pronk, Y.  Rutten, B.  van Tienen, T. G.  Nijhuis-van der Sanden, M. W. G.  van Cingel, R. E. H. | 2020 | International Journal of Sports Physical Therapy | Less than half of ACL-reconstructed athletes are cleared for RTP based on practice guideline criteria: Results from a prospective cohort study | IJSPT | Prospective observational cohort study | -  (n= 108) | JBI cohort  11/11 | 15 |
| Von Aesch, A. V.  Perry, M.  Sole, G. | 2016 | Physical Therapy in Sport | Physiotherapists' experiences of the management of anterior cruciate ligament injuries | Elsevier | Qualitative study | -  (n=15) | JBI Qualitative  10/10 | 14 |
| Witjes, S.  Hoorntje, A.  Koenraadt, K. L. M.  Goossens, P.  KerKHoffs, G. M. MJ.  van Geenen, R. C. I. | 2018 | Acta Orthopædica Belgica | Considerable variety in usual care rehabilitation after knee arthroplasty: a survey amongst physiotherapists | - | Cross sectional observational study | 62% (n=82) |  | 17 |
| **Rehabilitation specialists** | | | | | | | | |
| **Hamstring injuries** | | | | | | | | |
| Di Trani Lobacz, A.  Glutting, J.  Kaminski, T. W. | 2016 | Journal of Athletic Training | Clinical Practice Patterns and Beliefs in the Management of Hamstrings Strain Injuries | National Athletic Trainers’ Association, Inc. | Cross sectional study - Electronic survey | 17% (n=1356) |  | 18 |
| McVeigh, F.  Pack, S. M. | 2015 | Journal of Sport Rehabilitation | An exploration of sports rehabilitators' and athletic rehabilitation therapists' views on fear of reinjury after anterior cruciate ligament reconstruction | Human Kinetics, Inc. | Qualitative study – purposive sampling | 1% (n=8) | JBI qualitative 10 | 17 |
| **Multidisciplinary Team** | | | | | | | | |
| **Generalised RTP / injury vignettes** | | | | | | | | |
| Barrette, A.  Harman, K. | 2020 | Journal of Sport Rehabilitation | Athletes Play Through Pain—What Does That Mean for Rehabilitation Specialists? | Human Kinetics | Qualitative with a focused ethnography method | -  (n=3) | JBI qualitative 10 | 16 |
| Beardmore, A.L.  Handcock, P. J.  Rehrer, N. J. | 2005 | Physical Therapy in Sport | Return-to-play after injury: practices in New Zealand rugby union | Elsevier | Questionnaire cross sectional study | 73% (n=58) |  | 16 |
| Boudier-Reveret, M.  Mazer, B.  Feldman, D. E.  Shrier, I. | 2010 | British Journal of Sports Medicine | Practice management of musculoskeletal injuries in active children. | BMJ | Cross-sectional survey | 33.6% (n=464) |  | 17 |
| Chen, Y.  Buggy, C.  Kelly, S. | 2022 | International Journal of Environmental Research and Public Health | Managing the Wellbeing of Elite Rugby Union Players from an Occupational Safety and Health Perspective | MDPI | Qualitative | -  (n=15) | JBI Qualitative tool 9 | 15 |
| Geldenhuys, A. G.  Burgess, T.  Roche, S.  Hendricks, S. | 2023 | Physical Therapy in Sport | Return to rugby following musculoskeletal injuries: A survey of views, practices and barriers among health and sport practitioners | Elsevier | Cross-sectional observational | 0.9% (n=64) |  | 20 |
| Hess, C. W.  Meyer, B. B. | 2022 | Journal of Sport Rehabilitation | Lived Experiences of an Elite Performance Management Team Through Injury Rehabilitation: An Interpretative Phenomenological Analysis | Human Kinetics | Qualitative | -  (n=5) | JBI Qualitative tool 10 | 16 |
| Horan, D.  Kelly, S.  Hägglund, M.  Blake, C.  Roe, M.  Delahunt, E. | 2023 | Sports medicine - open | Players', head coaches', and medical personnels' knowledge, understandings and perceptions of injuries and injury prevention in elite-level women's football in Ireland | Springer | Qualitative | -  (n=8) | JBI Qualitative tool 10 | 19 |
| Mazer, B.  Shrier, I.  Ehrmann Feldman, D.  Swaine, B.  Majnemer, A.  Kennedy, E.  Chilingaryan, G. | 2010 | Clinical Journal of Sports Medicine | Clinical Management of Musculoskeletal Injuries in Active Children and Youth | Lippincott Williams & Wilkins, Inc. | Cross sectional study | 34% (n=464) |  | 20 |
| Müller, P. O.  Helbling, M.  Verhagen, E.  Spörri, J.  Bolling, C. | 2024 | BMJ Open Sport & Exercise Medicine | 'I want to ski and race, not just ski': a qualitative study on athletes' and stakeholders' perspectives on return-to-sport in high-performance Snowsports | BMJ | Qualitative | -  (n=14) | JBI Qualitative tool 10 | 19 |
| Read, D.  Rosenbloom, C. | 2024 | Science and Medicine in Football | What contextual factors influence pain management decision making concerning player availability in professional men's football? a qualitative analysis of practitioner perceptions | Routledge Taylor & Francis Group | Qualitative | 21.8% (n=20) | JBI Qualitative tool 10 | 18 |
| Read, P. J.  Jimenez, P.  Oliver, J. L.  Lloyd, R. S. | 2018 | Journal of Sports Sciences | Injury prevention in male youth soccer: Current practices and perceptions of practitioners working at elite English academies | Routledge Taylor & Francis Group | Cross sectional, quantitative online survey | 55% (n=41) |  | 16 |
| Riendeau, C.  Parent-Houle, V.  Lebel-Gabriel, M. E.  Gauvin, P.  Liu le, Y.  Pearson, I.  Hunt, M. R. | 2015 | Journal of Orthopaedic *&* Sports Physical Therapy | An Investigation of how university sports team athletic therapists and physical therapists experience ethical issues | JOSPT | Qualitative | -  (n=11) | JBI Qualitative tool 10 | 17 |
| Shultz, R. Bido, J.  Shrier, I.  Meeuwisse, W. H.  Garza, D.  Matheson, G.O. | 2013 | Clinical Journal of Sports Medicine | Team clinician variability in return-to-play decisions | Lippincott Williams & Wilkins, Inc. | Survey questionnaire cross sectional study | 66.3% (n=67) |  | 19 |
| Shrier, I.  Safai, P.  Charland, L. | 2014 | British Journal of Sports Medicine | RTP following injury: Whose decision should it be? | BMJ | Online survey cross sectional study | 24% to 37% (n=736) |  | 19 |
| Shrier, I.  Serner, A.  Wangensteen, A.  Steele, R.J.  Weir, A. | 2017 | Journal of Science and Medicine in Sport | Measuring heterogeneity of re-injury risk assessments at the time of clearance to RTP: A feasibility study | Elsevier | Feasibility study with a descriptive prospective case-series. | -  (n=10) |  | 17 |
| Yeomans, C.  Comyns, T.M.  Cahalan, R.  Warrington, G.D.  Harrison, A.J.  Hayes, K.  Lyons, M.  Campbell, M.J.  Kenny, I.C. | 2018 | Physical Therapy in Sport | Current injury monitoring and player education practices in Irish amateur rugby union | Elsevier | Cross sectional study – survey design | 76% (n=44) |  | 18 |
| **Shoulder injuries** | | | | | | | | |
| Maher, N.  Willmore, E.  Bateman, M.  Blacknall, J.  Chester, R.  Horsley, I.  Gibson, J.  O' Sullivan J  Jaggi, A. | 2024 | Shoulder & Elbow | Rehabilitation following shoulder arthroscopic stabilisation surgery: a survey of UK practice | Sage | Cross-sectional | -  (n=138) |  | 19 |
| **Hip injuries** | | | | | | | | |
| Worner, T.  Thorborg, K.  Moksnes, H.  Eek, F. | 2018 | Knee Surgery, Sports Traumatology, Arthroscopy | Similar views on rehabilitation following hip arthroscopy  among physiotherapists and surgeons in Scandinavia: a specialized care survey | Springer | Cross sectional survey | -  (n=90) |  | 17 |
| **Hamstring injuries** | | | | | | | | |
| Balcı, Aydın  Ülkar, Bülent | 2020 | Turkish Journal of Sports Medicine | Return to Sports After Hamstring Injuries: Importance of the Criteria and Their Applicability in Clinical Practice | Journal of sports medicine | Cross sectional survey study | 51%-69% (n=75) |  | 17 |
| Dunlop, G.  Ardern, C.L.  Andersen, T.E.  Lewin, C.  Dupont, G.  Ashworth, B.  O’Driscoll, G.  Rolls, A.  Brown, S.  McCall, A. | 2020 | Sports Medicine | Return‑to‑Play Practices Following Hamstring Injury: A Worldwide Survey of 131 Premier League Football Teams | The Author(s) | Cross sectional study | 42% (n=131) |  | 18 |
| **Knee injuries** | | | | | | | | |
| Ebert, J. R.  Webster, K.E.  Edwards, P. K.  Joss, B. K.  D’Alessandro, P.  Janes, G.  Annear, P. | 2019 | Physical Therapy in Sport | Current perspectives of Australian therapists on rehabilitation and return to sport after anterior cruciate ligament reconstruction: A survey | Elsevier / Science Direct | Cross sectional Survey-based study | -  (n=223) |  | 14 |
| Lambert, C.  Rotzmann, R.  Ellermann, A.  Carvalho, M.  Akoto, R.  Wafaisade, A.  Lambert, M. | 2020 | The physician and sportsmedicine | Return to competition after anterior cruciate injuries in world class judoka | Taylor & Francis Group | Cross sectional survey | -  (n=50) |  | 18 |
| Lyng. K. D.  Rathleff, M. S.  Dean, B. J. F.  Kluzek, S.  Holden, S. | 2020 | Scandinavian Journal of Medicine & Science in Sports | Current management strategies in Osgood Schlatter: A cross-sectional mixed method study | Wiley Online Library | Cross-sectional mixed-method study using questionnaire and semi-structured interviews | -  (n=255) | JBI Qualitative 8/10 | 16 |
| Lower leg injuries | | | | | | | | |
| Green, B.  McClelland, J. A.  Semciw, A. I.  Schache, A. G.  McCall, A.  Pizzari, T. | 2022 | Sports medicine - Open | The Assessment, Management and Prevention of Calf Muscle Strain Injuries: A Qualitative Study of the Practices and Perspectives of 20 Expert Sports Clinicians | Springer Open | Qualitative –  In-depth interviews | 76.9% (n=20) | JBI Qualitative tool 10/10 | 17 |
| **Ankle and foot injuries** | | | | | | | | |
| Aguilaniu, A.  Delvaux, F.  Schwartz, C.  Martens, G.  Forthomme, B.  Kaux, J-F.  Croisier, J-L. | 2023 | Physiotherapy Research International | Survey of physicians' and physiotherapists' ankle muscle strength assessment practices for safe return to sports after lateral ankle sprain: A short report. | Wiley | Cross-sectional | 93.8% (n=212) |  | 19 |
| Johnson-Lynn, S.  Townshend, D. | 2017 | Journal of Surgical Education | How Knowledge Relates to Confidence in Orthopedics and Emergency Medicine Regarding Return to Sport and Rehabilitation in Foot and Ankle Trauma | Clinical Key | Questionnaire | 51%  (n=1020 |  | 17 |

Supplementary Table 5: Characteristics of included studies in the systematic review

### Medical doctors

| **Author** | **Type of study & data collection** | **Participant’s profession** | **Sample size** | **Purpose** | **Injury location** | **RTP level – patients activity level profile** | **RTP practices** | **Conclusions** |
| --- | --- | --- | --- | --- | --- | --- | --- | --- |
| **RTP practices** | | | | | | | | |
| Anderson, L. C.  Gerrard, D. F. | Questionnaire | Sports doctors in New Zealand   - 17/18 work in a MDT | 18 | Identify ethical issues in sport. | General RTP practice unspecified MSK injury | Individual athletes and teams ranging from national level, club teams and school teams | Conflicts within decision-making:   - Conflicts between the patients injury, sport requirements needed and the pressure to RTP - Pressure placed on the doctor by the coach or athlete to allow a quicker RTP - Doctors concerned that an early RTP after injury = attached risk or long term consequence - The athlete, coach or other team was identified as the source of pressure - Some doctors expressed concern that athletes were not always honest about their injury – especially head injury. A few doctors gave a reason for this being that athletes were fearful that the doctor would inform the coach or team management.   Doctors responsibilities:   - Conflicts of interest –shared responsibility - In conflict 89% (n=16) athlete comes first (primary responsibility) - All doctors felt somewhat responsible to the athlete - 72% (n=13) felt responsibility to the coach - 55% (n=10) responsibility to the manager or management team - 44% (n=8) sense of responsibility to other team members - 28% (n=5) felt responsible to themselves. - Other groups listed = 33% (n=6) medical council, 11% (n=2) other sports physicians, 5.5% (n=1) other team members   The type of relationships:   - Relationship between the doctor and team to address RTP issues was a concern for a small number of doctors - Concern over the use of analgesics to permit an athlete to continue playing - Concern over time pressure for an adequate on-field assessment   National and regional teams have higher expectations when the stakes are raised. | Ethical issues are reported in this study that serves to begin the process of identifying the wide variety of ongoing problems that concern sports doctors. Further study into this topic is needed. |
| Hobusch, G. M., Keusch, F., Tsuchiya, H., Joyce, M., Windhager, R. | Web survey | Professional organizations of tumour reconstructive surgeons: the European Musculoskeletal Oncology Society (EMSOS), the International Society of Limb Salvage (ISOLS) and the Musculoskeletal Tumour Society (MSTS) | 76 | This study surveyed orthopaedic tumour surgeons who regularly do lower-extremity bone tumour and mega-prosthetic replacements in active younger patients. The purpose of the study was to help determine sport activity possible for these patients after treatment. | Hip or knee mega-prosthetic treatment | Younger, active cancer survivors (not clearly defined) | Surgeons opinions:   - 85% (38% & 47%) = sport is important for well-being of patients after modular bone-joint replacement - 88% (44% &44%) = sport has a positive effect for bone sarcoma survivors - >50% encourages survivors to participate in sport that they can do regularly - Fear of sports participation: - Periprosthetic fracture - Allograft failure/fracture - Loosening - Prosthetic or bearing fracture - Early polyethylene wear - *the fear of complications seemingly worse than the actual witness of complications - 64% allow RTP if patient had prior experience in that sport vs 36% who said it made no difference - Type of sport: - walking, swimming and stationary cycling is the most recommended sporting activity   Surgeons from Europe and the Americas are more inclined to encourage a higher level of activity compared to Asian Pacific  e.g. 71% of surgeons from Europe and America will allow golf compared to 29% from Asia Pacific | The study provided some insight into some of the sport recommendations made by surgeons. Surgeons understand that sport activity is important, however it is important that patients understand the potential risks of returning to higher levels of sport. The study highlights the need for objective data to determine the relation between activity levels and prosthetic failure. |
| Schrock, J. B.  Carver, T. J.  Kraeutler, M. J.  McCarty, E. C. | Descriptive epidemiology study | Orthopaedic team physicians   - 81-93% fellowship trained - 90% of orthopaedic team physician were orthopaedic surgeons | 2008 = 31  2016 = 29 | To update the literature on treatment preferences of National Football Leag (NFL) team physicians from 2008 to 2016 | Multiple MSK injury:  Knee: ACL, MCL, PCL  Shoulder:  Anterior shoulder dislocation, ACJ  Elbow: UCL  Thumb: UCL  5^th^ metatarsal fractures  Lower leg: Tibial fractures | Football (NFL) | ACL  Surgeons allowing RTC at 6 months  2008- 49% vs 2016 – 14%  Surgeons allowing RTC at 10-12 months  2008- 4% vs 2016 – 24%  PCL  Gr1-2 - Same day RTP  2008 – 22% vs 2016 – 14%  Anterior shoulder dislocation  Athletes RTP without surgery needed to use a harness 2008 – 93% vs 2016 – 90%  After surgery surgeons allowing RTC at 6 months  2008- 40% vs 2016 – 48%  Majority did not require a harness for RTP post ant shoulder stabilisation  ACJ  Gr 1-2 physicians use local anaesthetic injection  2008 – 60% vs 2016 – 86%  The use of local anaesthetic injections prior to games to allow for RTP  2008 90% vs 2016- 100%  UCL – thumb  Casting and allowing RTP  2008 – 63% vs 2016 – 55%  5^th^ metatarsal fractures  RTP at 7-9 weeks  2008 – 59% vs 2016 – 52%  RTP at >10weeks  2008 – 17% vs 2016 – 34%  Tibial fractures  100% of surgeons in 2008 & 2016 preferred not to remove the intermedullary nail prior to RTP  Toradol injections  The use prior to a game to assist nagging injuries  2008 – 93% vs 2016 – 48%  Administering >5 Toradol injections prior to a game  2008 – 75% vs 2016 – 28% | RTP treatment preferences became more conservative over 8-10 years |
| **Spine injuries** | | | | | | | | |
| Abla, A. A.  Maroon, J. C.  Lochhead, R.  Sonntag, V. K.  Maroon, A.  Field, M. | Emailed survey | Neurosurgeons and orthopaedic  spine surgeons   - Spine specialists - all geographic areas of America - members of the North American Spine Society | 523 | Surgeons RTP opinions and advice on patients wanting to return to golf after either cervical or lumbar surgery, with or without fusion | Cervical or lumbar spine surgery | Non athlete and athlete golfers | Patient asks when can he/she return to golf?  Scenario dependant RTP advice.  Scenario 1: L3-5 Laminectomy  4-8 weeks RTP (most common answer)  2-3 months (2^nd^ most common answer)  Scenario2: Left L4-5 microdiscetomy  4-8 weeks RTP (most common)  2-3 months (2^nd^ most common answer)  Scenario 3: C5-6 ACDFP  2-3 months RTP most common  4-8 weeks RTP (2^nd^ most common answer)  Scenario 4: L4-5 Laminectomy & fusion  6months RTP (most common)  2-3 months (2^nd^ most common answer)  Surgeons hardly ever chose “never golf again”  In 2/5 scenarios: tissue healing time and follow-up radiographs needed before RTP (golf)  Other considerations in RTP decisions:   - degree of incisional pain - muscle strength and tightness - tissue healing - athletic level (athletes quicker RTP than non-athletes) - Type of player position (ball hitter vs putter) - Injury/surgery dependant: Complex injury scenarios = ↑recovery time and more doctors choosing no RTP - Physical therapy, rehabilitation and gradual introduction to technique (putting/chipping before full swings) important RTP considerations. - Patients should be pain free in their swing and other movements. - Sex and age of patient didn’t affect RTP | - RTP: Bony fusion at 2-3 months post cervical fusion and > 6 months post lumbar fusions. - More surgeons would allow an elite golfer to RTP sooner if there was financial pressure to RTP - Important factors to facilitate RTP: swing technique that reduces lower back pain, rehabilitation, core strengthening, spinal flexibility and cardiovascular fitness. - Patient related factors such as age, conditioning level, determination to RTP can create wide variations in RTP decisions.   Spine surgeons recommendations for RTP (golf) after surgery. The study aimed to guide surgeons and their patients regarding time to RTP after spinal surgery. |
| Backer, H. C.  Johnson, M. A.  Hanlon, J.  Chan, P.  Turner, P.  Cunningham, J. | Questionnaire | Surgeon members of the Spine Society of Australia (SSA) | 31 | To assess whether a consensus exists on surgeons opinions about RTP, the importance of sport following single level discectomy and the influence of the surgical technique | Single level discectomy | Highly active patients | Importance of sport in management:  RTP decision:   - 83.9% of surgeons discuss post-operative RTP - 16.1 only talk about this topic if the patients ask specifically   RTP based on:   - 45.2% Pre-operative level is influential - 16.1% morphology/anatomy of the disc - 6.5% body mass index - 3.2% age   Risk factors for recurrent prolapse   - 35.5% the size of the annular defect - 29.0% patient body habitus - 25.8% size of prolapse - 12.9% location of disc prolapse - 6.5% preoperative neurological impairment - 9.7% postoperative sport engagement   RTP importance to certain sports:   - 41.9% important - 19.4% very important - 35.5% neutral - 3.2% not important   View that sport improves function:   - 71.0% important - 19.4% neutral - 9.7% unimportant   Recurrent disc herniation risk due to sport:   - 32.3% neutral - 25.8% negative - 22.6% no negative impact - 19.4% unsure   Type of sport chosen by patients post-surgery:   - 51.6% about right - 29.0% low or too low - 19.3% high or too high   Surgical procedure and postoperative recommendation:   - 80.6% Type of surgery is not based on the preoperative level of activities - 50.0% open approach - 33.3% a minimal invasive technique - 16.7% arthroplasty - 58.1% surgical technique does not affect the outcome   RTP - no consensus for high performing athletes:   - 9.7% immediate RTP to previous level - 16.1 leave it up to the patient - 19.4% RTP on lower levels - 16.1% no more high impact sports - 32.3% previous level of activity however, over a certain period of time ranging from 6 weeks to 3 years postoperatively - 3.2% dependent on the annular defect size of the disc - 3.2% biomechanical alignment of the spine   RTP time:  High impact   - 22.6% 6 weeks - 48.4% 3 months   Low impact   - 38.7% 3 weeks - 51.6% 6 weeks   Type of sport  Unlike the timing no consensus for majority of sports was found except for weightlifting/CrossFit. Many sports listed in figure. | Functional outcomes are reported when patients are able to return to sport after discectomy. However, no system or rehabilitation protocol was found which highlights the importance of this study. According to the members of the Spine Society of Australia, there are some uncertainties especially in weightlifting, contact sports and timing when to return to sports exist. No correlation between individual surgeons activity, level of activity or the medical training was observed. Therefore, current research should focus on large cohort prospective randomized control trials comparing early with delayed return to activities. In addition, biomechanical studies should be performed to assess the impact of different activities on the intervertebral disc space before and after discectomy. Further prospective spine registries will help to gain more accurate data to assess long-term functional outcome and the revision rate for recurrent disc herniation. |
| France, J.C.  Karsy, M.  Harrop, J.S.  Dailey, A.T. | Survey | Surgeons   - Spine Trauma Study Group | 25 | Expert opinion regarding cervical spine injury to assist the treating physician incorporate unique factors of the case in RTP decisions. | Cervical spine injuries | 16 -21 year old athlete case scenarios in high contact sports such as horse riding, diving, ice hockey, football, wrestling, skiing. | Type of injury: Case dependant.  Category: cervical neurapraxia and stenosis:   - episodes of CCN with early resolution of symptoms and no stenosis on MRI : 64% -88% allowed a return to high contact sport. - Diffuse stenosis and early resolution of CCN: 27% -35% would allow return to high contact sport - In all 4 cases under this category surgeons usually recommended 2-4 weeks RTP. - Category: atlantoaxial cervical injury with or without fusion - C1 ring or C2 hangman’s fracture healing nonoperatively: 67% would allow return to high contact sport, with fusion only 20%. - In both cases surgeons recommend 2 - >6months RTP   Category: sub-axial cervical injury with or without fusion   - C5-6 unilateral facet dislocation, neurologically intact with subsequent posterior fusion 56% would allow return to high contact sport. - Herniated disks repaired operatively 71% would allow return to high contact sport. - In all 3 cases surgeons recommended 2 - >6months RTP   Recommendations for X-rays (64 to 92%), CTs (20 to 84%), and MRIs (50 to 100%) varied among different cases, indicating a high consensus for obtaining imaging in making recommendations. | Most surgeons agreed in on the RTP advice. However, the RTP questions were limited. Reasonable consensus was reached for the type of sport the athlete can return to after cervical spine injury.  Surgeons use time based criteria and extensive imaging with radiographs, CT and MRI before advising RTP. |
| Ho, D.  Du, J. Y.  Erkilinc, M.  Glotzbecker, M. P.  Mistovich, R. J. | Questionnaire (emailed) | Orthopaedic surgeons   - Paediatric Orthopaedic Society of North America (POSNA) - North America - 137 (80.6%) specialised in paediatric orthopaedics - 26 (15.3%) specialised in spine and paed ortho - 7 (4.1%) specialised in spine | 170 | To understand the recent trends, physician demographics and patient characteristic that affect RTP decisions after posterior spinal fusion (PSF) in patients with adolescent idiopathic scoliosis (AIS). | Adolescent Idiopathic Scoliosis – spine | Clinical scenarios that relate to different adolescent athletes and sport activity types:   - Jogging - Non-contact:   -gym class  -swimming  -recreational activity  -cross country running  -sprinting  -no stunt cheerleading   - Contact:   -soccer,  -basketball  -cheerleading  -volleyball   - Collision   -NFL  -rugby  -hockey  -MMA  -Wrestling | Higher sport demand = more time to RTP (reported in months)   - jogging: 4.1; noncontact: 4.6; contact: 6.8; and collision: 9.8   Patient characteristics (such as sex, age, obesity, skeletal maturity, levels fused, and fusions ending in thoracic versus lumbar spine) did not change RTP recommendations for jogging, noncontact, contact, or collision activities.  Surgeon-related factors [such as volume, experience (esp. 16-20 years’ experience), fellowship type (esp paediatric ortho), and practice setting] all affected RTP. Surgeons with prior complications (11.8%) from RTP delayed return to collision activities (9.4,) vs surgeons without complications (7.2).  27 Complications found including broken rods, endcap failures, screw pull out, other  Routine physical therapy prescription:  142 (83.5%) No  12 (7.1%) Yes  16 (9.4%) under special circumstances | Compared to previous studies, POSNA members allow earlier RTP after PSF in AIS.  RTP varied based on practitioner-related factors rather than patient related factors. Surgeons with more experience and case volume allowed faster RTA showing increased confidence.  Although complications are low using current RTP protocols, these protocols represent expert opinion rather than evidence-based protocols. |
| Morganti, C.  Sweeney, C.A.  Albanese, S.A.  Burak, C.  Hosea, T.  Connolly, P.J. | Questionnaires | Spinal surgeons | 113 | Understand the influencing factors (such as published guidelines, type of sport, years in practice, subspecialty interest and sports participation) that effect spinal surgeons RTP decision-making after a cervical spine injury. | Cervical spine injury | Not described in the case scenarios | 105/113 surgeons have given RTP advice with only 49% of surgeons using published guidelines to assist decision-making  Surgeons often used the hierarchy of risk i.e. type of sport: collision (football, hockey), contact (lacrosse, basketball), non-contact high velocity (skiing, gymnastics), non-contact repetitive load (running), non-contact low impact (golf, bowling), no sports.  Years of practice: a lower level of play was generally recommended by more senior surgeons  Speciality: surgeons with a spine subspeciality interest generally recommend a higher RTP.  Surgeons previous sports participation didn’t have an influence on the result | Varied opinions especially because of surgeon-related factors such as improved treatment technique, knowledge, experience and career bias.  This study highlighted the need for utilisation and development of criteria to assist surgeons RTP decisions after cervical injury. |
| Ukogu, C.  Bienstock, D.  Ferrer, C.  Zubizarreta, N.  McAnany S.  Chaudhary, S. B.  Iatridis, J. C.  Hecht, A. C. | Prospective survey study | Spine surgeons | 62 | Determine the level of competitive sport and impact allowed by surgeons treating significant cervical spine injury . | Cervical spine injury | - Type of sport: - maximal impact sports (football, lacrosse, rugby, lacrosse, boxing) - moderate impact (soccer, basketball, soccer skiing) - minimal impact (running, cycling, golf, bowling, baseball, swimming) - no sports - The level of play: - professional/collegiate - high school - recreational - none. | Proposed guidelines are not consistently being used by surgeons to determine a patients RTP: 70% respondents do not use either the Watkins or Torg guidelines to make decisions.  Per clinical scenario:   - High levels of variability for the type of sport and the level of play. - practitioner related factors include: years in practice, percent of sports-related activities in the surgeon’s practice, surgeon’s previous athletic experience, and the surgeon’s orthopaedic board certification status   More surgeons allowing RTP for maximal impact sports:   - spinal orthopaedic board certification - previous college/professional athletic level experience   Importance of athlete level in RTP decision:  Not important 6.82%  Somewhat important 38.67%  Important 34.09%  Very important 20.45% | Poor consensus amongst spine surgeons for RTP after cervical spine injury. Published guidelines (Watkins and Torg guidelines) are not being used. RTP decision-making is subjective and affected by demographic factors. Development of standardised guidelines need to be considered. |
| **Thoracic injuries** | | | | | | | | |
| McAdams, T. R.  Deimel, J. F.  Ferguson, J.  Beamer, B. S.  Beaulieu, C. F. | Survey | NFL team physicians  No other information stated | 23 | To present 2 cases of chondral rib injuries in the NFL and discuss the current practice patterns for management of these injuries among the NFL team physicians. | Chondral rib fractures | 2 clinical scenarios discussed: 25 year old line-backer NFL and 26 year old defensive back | Diagnostics: physicians used plain films to rule out rib fracture but if negative and still suspicious of a rib injury 70% utilized CT scans. 43% physicians used MRI for diagnosis especially if CT was negative.  57% use anaesthetic blocks to speed up RTP in both the acute and elective setting.  39% of physicians use elective anaesthetic blocks for subsequent games.  4% (one team) never offers or uses anaesthetic block for rib fractures.  Two cases discussed:  No specific criteria was used to determine safety for the athlete to RTP.  From the cases RTP was 2-4 weeks.  1 case used protective device in RTP.  Pain was the limiting factor for RTP for the athlete. The athlete that utilized the anaesthetic block was able to RTP quicker (2 weeks) than the athlete that refused (4 week). The athlete that received anaesthetic block had longer lasting discomfort (8 weeks vs 6 weeks respectively) | RTP can be up to four weeks which can be assisted using a protective device and anaesthetic block can be considered to speed up RTP only after risks and benefits are discussed with the athlete. |
| **Shoulder injuries** | | | | | | | | |
| Golant, A.  Christoforou, D.  Zuckerman, J. D.  Kwon, Y. W. | Web- based survey | Surgeons   - American Shoulder & Elbow Surgeons (ASES) | 94 | Summarise the RTP recommendations for shoulder arthroplasty patients | Shoulder arthroplasty | General population  - Athletic activity | 45.7% of surgeons allow RTP at 3-6 months.  All sports and all arthroplasty options: 74.1% of the respondents allowed some return to athletic activity. Surgeons based RTP advice on type of sport esp. contact load.  % of surgeons allowing RTP:   - 95.8% allowed cycling and other low-load sports - 21.4% allowed contact sports such as football and rugby   All arthroplasties analysed together:   - 90% allowed participation in non-contact low-load sports, such as golf and swimming. - 51% of surgeons allowed participation in contact sports, such as hockey and football   Across all activities, the % of respondents allowing some level of RTP   - Humeral head replacement (92.0%) - Humeral hemiarthroplasty (87.2%) - Total shoulder arthroplasty (76.5%) - Total shoulder resurfacing (74.2%). - Reverse total shoulder arthroplasty (45.2%) | RTP based on type of surgery and type of sporting load. High impact sports such as rock climbing, martial arts, football, rugby, hockey, and lacrosse pose a high risk injury to the shoulder and are the least recommended activity by surgeons. |
| Moore, David M.  Hurley, Eoghan T.  Mullett, Hannan | Online survey administered via email and annual meeting | Orthopaedic surgeons   - American Shoulder and Elbow Surgeons (ASES) - British Elbow and Shoulder Surgeons (BESS)   Average length practice = 10 years | 98  31 BESS  67 ASES | Current management of anterior glenohumeral instability. The different in practice between the two societies (ASES and BESS) were compared) | Anterior glenohumeral instability - shoulder | Rugby Union players (15 year old and 20 year old case study) | Time based criteria for RTP (weeks)  ASES vs BESS  RTP following first dislocation:  Conservative treatment  12 vs 13  Arthroscopic stabilisation  23 vs 18  Open bankart repair  23 vs 18  Laterjet procedure  22 vs 18  RTP following recurrent dislocations:  Conservative treatment  12 vs 12  Arthroscopic stabilisation  24 vs 18  Open bankart repair  24 vs 18  Laterjet procedure  20 vs 17  British surgeons are more prepared to allow players to return to contact sooner than American surgeons. | Further research of the optimal RTP time to reduce re-injury after anterior shoulder instability treatment will assist surgeon decision-making. BESS surgeons allow quicker RTP after arthroscopic stabilisation and Bankart repairs than ASES.  Suggestions from this study include, regulating the level of contact to enforce player safety and to limit contact levels in younger age groups. |
| Sharareh, B.  Edwards, T. B.  Shah, A.  Shybut, T. | Cross- sectional study | Orthopaedic surgeons | 242 | Variability in practice patterns including influencing factors among fellowship-trained orthopaedic shoulder surgeons. | Shoulder Laterjet procedure | Not described | Clearance to RTP including contact or collision sports:  13.2% 3 months  37.2% 4-5 months  42.1% >6 months  3.7% >9months  3.7% case dependant | The variation in RTP timeline post Laterjet suggests a lack of validated criteria-based post-operative rehabilitation protocols. |
| **Elbow injuries** | | | | | | | | |
| Sambare, N. D.  Chalmers, P. N.  Camp, C. L.  Bowman, E. N.  Erickson, B. J.  Sciascia, A.  Freehill, M. T.  Smith, M. V. | survey | Surgeons trained in hand/upper extremity, shoulder/ elbow, or sports-medicine | 24 | The purpose of this study is evaluate surgeon variability in the presurgical, surgical, and postsurgical treatment of MUCL injuries regarding the imaging modalities used for diag- nosis, indications for acute surgical treatment, and postoperative treatment recommendations for rehabilitation and return to play (RTP). | Medial ulnar collateral ligament (MUCL) reconstruction and repair with internal brace | Throwing and non-throwing athletes  -specifically baseball and softball (pitching) | Start a throwing program  Time criteria   - MUCL without a brace   - 45% 4 months - MUCL with a brace   - 54% 3months   - 17% 4 months   Other criteria   - Functional progression   RTP to field position  Time   - MUCL recon without brace   - 46% 6 months - MUCL with internal brace   - 42%-45% 6 months (3-7 months)   Baseball and softball   - Variable time point agreement: return to throwing from the mound, return to unrestricted pitching, and return to hitting. - Return to throwing off the mound was most commonly allowed at 6 or 7 months after MUCL reconstruction and was more variable after MUCL repair with internal brace.   Return to unrestricted pitching   - criteria was completion of the throwing program regardless of the time since surgery   - baseball: 58.3% for MUCL reconstruction and 50% for MUCL repair with internal brace   - softball: 66.7% for MUCL reconstruction and 50% for MUCL repair with internal brace).   Respondents with >20 MUCL surgeries per year:   - 75% respondents to start a formal throwing program at 3 months after MUCL repair with internal brace. - All other RTP (common as well as sport-specific) milestones (for both reconstruction and repair) exhibited less than 75% agreement among these respondents.   RTP time varies:   - MUCL recon (2-8 months) - repair with internal brace (1-8months)   *6months most common time point across all nonthrowing sports except tennis and track  *There was no high-level agreement regarding time to RTP for any sport, including baseball and softball, regardless of the type of surgical technique.  *MUCL repair with internal brace, the time to RTP is more variable across sports than after reconstruction. | There is agreement for the indication of acute surgical treatment of distal MUCL tears, duration of bracing after surgery, and the time to initiate physical therapy after surgery. There is not clear agreement on indications for surgical treatment for every MUCL tear pattern, RTP time for throwing, hitting, and participation in nonthrowing sports. |
| **Hand and wrist injuries** | | | | | | | | |
| Dy, C. J.  Khmelnitskaya, E.  Hearns, K. A.  Carlson, M. G. | Cross sectional study | Hand surgeons | 37 | To determine recommendations for treatment, surgery and RTP for 10 common hand injuries. | Hand and wrist injuries | Surgeons affiliated to a professional sports team  Baseball (n=22)  Football (n=22)  Basketball (n=19)  Hockey (n=12) | RTP time for different hand and wrist injuries.  Metacarpal fracture (non-displaced)  Protected play:   - 56.8% RTP 3-4 weeks - 37.8% immediate RTP - Unprotected - 73% RTP   Scaphoid fracture (non-displaced)  Protected:   - 51.4% RTP protected 4-6 weeks - 32.4% immediate RTP protected - Unprotected - 24.3% 4-6 weeks RTP - 48.6% 6-12 weeks - 27% >12 weeks   Pisiform fracture  Treatment decision   - 29.7% immediate RTP - 18.9% immediate excision - 21.6% 4 weeks splinting prior to RTP - 8.1% excision after 4 weeks if not healed - 21.6% excision after 8 weeks if not healed   Hamate hook fracture  Protected play   - 30.6%immediate RTP - 52.8% 2 weeks RTP - 16.7% 6 weeks RTP - Unprotected play - 54.1% 6 weeks - 40.5% 2 weeks - 2.7% immediate RTP   Thumb collateral ligament injuries  Treatment decision   - 37.8% immediate repair - 32.4% repair at end of season (if within 3 months) - 18.9% repair at end of season (even if >6months post injury) - 2.7% non-operative - Protected play - 13.9% immediate RTP - 55.6% 2 weeks RTP - 27.8% 6 weeks RTP - 2.8% 3 months RTP - Unprotected play - 63.9% 3 months RTP   Stable proximal interphalangeal joint dislocation  Protected play   - 94.4% immediate RTP - 5.6% 4 weeks RTP   Unprotected play   - 29.7% 4 weeks RTP - 37.8% 4-8 weeks RTP - 8.1% 3 months RTP | Difficult to determine the best RTP when treating athletes. Individualised RTP is needed and must consider the athletes desires and demands, weigh up the benefits of an early RTP vs the risk of potential complications. Surgeons should collaborate in multicentre prospective studies in hand and wrist injuries regarding elite athletes. |
| **Hip, groin and pelvic injuries** | | | | | | | | |
| Domb, B.G.  Stake, C.E.  Finch, N.A.  Cramer, L. | Survey | Orthopaedic surgeons | 27 | Determine what percentage of surgeons recommend RTP after hip arthroscopy. | Hip arthroscopy | General population, not described | Time based criteria  70% surgeons suggested 12-20 weeks before return to competitive sport.  Objective Criteria:   - Single leg squat 19% - Single leg squat pain-free 56% - Lateral agility drills 19% - Lateral agility drills pain-free 70% - Run pain-free 70% - Jump pain-free 59% - All sport movements pain-free 85% - Minimal cartilage damage seen at arthroscopy 4%   High risk sports:   - Kick boxing or striking martial arts, football, basketball, wrestling   Medium risk:   - Surfing, volleyball, distance running, baseball, tennis, sprinting, golf   Low risk:   - Golf, distance running | Most surgeons suggest that athletes can RTP in 12 to 20 weeks after hip arthroscopy. Athletes must perform pain-free running, jumping, lateral agility drills, and single-leg squats before RTP. Sports with high impact and ROM of the hip, such as football and martial arts are high risk. Sports with low impact and low ROM, such as golf, are low-risk sports for RTP. |
| Vu-Han, T.  Hardt, S.  Ascherl, R.  Gwinner, C.  Perka, C. | Questionnaire emailed | Surgeons   - specialized in arthroplasty - German   Arthroplasty Society  82.8% >10 years surgical experience  52.5% >20 years surgical experience | 99 | Identify the current practice trends including influencing factors of total hip arthroplasty (THA) to help RTP decisions in patients with high RTP expectations. | Total hip arthroplasty | Not described | Recommendations:  High impact sports:   - 51.5% if received adequate training - 8.1% without limitations - 34.3% not recommended - 3% up to the patient - Low impact sports - 72% without limitations   Type of sports/activity   - Not recommended (or only with adequate training): basketball, boxing, soccer, gymnastics, handball, hockey, squash, climbing, volleyball, tennis, and skiing on slopes - Recommended (without limitations or training): Walking, swimming, hiking, and level biking - Varying opinions: Ballroom dancing, cross country biking, bowling, dancing, e-scooters, fitness/weights, golf, horseback riding, jogging, Pilates, cross country skiing, table tennis, and yoga   Time based criteria  Low impact sports   - 3months 72% - 6 months 22% - Not recommended 1% - Undecided 1%   High impact sports   - 3 months 10% - 6 months 50% - Not recommended 32% - Undecided 1%   Surgeons opinions on whether they consider physical activity important:   - 81.9% in favour of PA after THA - 58.6% surgeons did not think that RTP had a negative impact on the longevity of the hip implant - 79.8% expected a positive impact of sports on the longevity of hip implant - Data suggested that many experts (~ 39.4%) would encourage patients to increase overall PA. | This study suggested that most surgeons include their patients lifestyle and sport activity expectations in their assessment. Patients should be encouraged to be more active as complications associated with RTP are considered minimal. High expectations for RTP can influence surgical decision-making.  Recommendations for RTP in higher impact sport is increasing due to improving implant material and surgical techniques. This study highlighted the need for future studies with updated implant registry data to show whether the developments shown in this study are justified. |
| **Knee injuries** | | | | | | | | |
| Arliani, G.G.  Pereira, V.L.  Leao, R.G.  Lara, P.S.  Ejnisman, B.  Cohen, M. | Cross sectional study | Orthopaedic surgeons and traumatology specialists | 61 | To describe ACL injury treatment in Brazilian major league  professional soccer players. | ACL - knee | Professional soccer players in Brazil | Unrestricted RTP post operatively was allowed:   - 65.6% 6 to 8 months - 24.6% 8 to 10 months   - 8.2%, 4 to 6 months  - 1.6 % > 10 months  Parameter for RTP   - 49.2% isokinetic strength test - 23% rehabilitation and postoperative time longer than 6months - 13.1% normal and painless physical examination - 9.8% hop test - 8.2% physiotherapy assessment - 8.2% other parameters (among them the combination of the previously mentioned parameters with kinematics and force platform, comparative contralateral functional tests, and the application of the “Functional Movement Screen”   Surgeons (%) belief in the % athletes that return to professional level post ACLR  90% of athletes: 73.8%  80-90% of athletes: 18%  60-80%: 8.2% | Time to surgery is 1-4 weeks after ACL injury.  Most surgeons allow their players to RTP after six to eight months of surgery. The main factor used to allow RTP is isokinetic strength testing. Surgeons do not use post-operative functional braces. |
| Bakowski, P.  Bakowski-Zywicka, K.  Pointek, T. | Survey study – Cross sectional | Registered orthopaedic surgeons   - Poland | 205 | The purpose of this study was to determine the treatment practices of Polish orthopaedic surgeons treating meniscus tear injuries. | Meniscus repair – knee arthroscopic procedures | Athletic population – example given of 18 and 30 year old footballer | RTP decision makers: In most cases, either the surgeon alone or the surgeon together with a physical therapist. Slightly more orthopaedic surgeons favoured the RTP decision to be shared compared to being made in isolation.  Most important decision-making criteria was:   - functional state (80% = 93% of experts and 74% non-experts) - use of objective physical tests (78%) - Use of functional tests 58% - Lack of discomfort (42%) - Time since surgery (35%) - Use of dynamometer (28%) - Imagining (19%) - Use of subjective surveys (17%) | This survey offered useful RTP recommendations in the management of meniscus injury (arthroscopy). In most meniscal treatment options there was agreement between experts and non-experts however rehabilitation differed between groups. |
| Betsch, M.,  Darwich, A.,  Chang, J.,  Whelan, D.,  Ogilvie-Harris, D.,  Chahal, J.,  Theodoropoulos, J. | Semi-structured interviews | Orthopaedic surgeons  specialising in either: orthopaedic sports medicine, orthopaedic trauma and upper extremity reconstruction | 18 | The exploration of the current RTP criteria for ACLR used in elite athletes. The study aimed to understand the decision making process of professional team physicians. | ACLR - knee | - 50% National Hockey League (NHL) - 11% National Basketball Association (NBA) - 11% National Football League (NFL) - 11% Major League Baseball - 6% Major League Soccer (MLS)   11% - collegiate level sport | Four main themes discussed   1. Informative feedback  - Interdisciplinary decision making   including athletic trainers, physiotherapists, S&C coaches, psychologists and sport scientists   - External pressures   Incl general management, coaching staff, parents, families, teammates, players, financial situation, stage of career, stage of season, and team standing in the league   1. Objective findings  - Clinical signs and tests   Incl muscle girth, absence of knee joint effusion, ROM, stability tests   - Functional tests   No standardised battery of test  Single leg hop and triple hop test used most   - Objective strength   Computerised testing such as Biodex and Cybex used for elite athletes   - Radiographic studies   Not used routinely   1. Subjective findings  - PROM’s   Limited value   - Psychological scores   Not regularly used but do ask screening questions to determine if referral to sport specific psychologist is needed   1. Timeframe and Type of sport  - Type of sport: cycling and swimming can be 3-4 months RTP vs pivoting sports can be 12 months or longer - RTP 6-12 months most commonly - Pain free sporting movements   *Main criteria for RTP was listed in this study based off the above themes and findings. | Four main themes where identified in this study however even with professional team physicians, the ranking of RTP criteria is inconsistent and a standardised set of guidelines was not able to be determined from this study. The study highlighted the need for future research. |
| Coskunsu, D.  Bayrakci Tunay, V.  Akgun, I. | Questionnaire study | Turkish orthopaedic surgeons | 39 | To determine the surgical approaches and rehabilitation protocols for ACLR  used by Turkish orthopaedic surgeons and to compare their results with the data of “ACL Study Group”. | ACLR - knee | Not described | RTP contact:  4-5 months = 16.4%  Over 6 months = 83.6%  RTP without contact:  4-5 months = 50.9%  Over 6 months = 45.5%  RTP tests:  Routine strength test application   - Hamstring tendon graft = 45.7% (n=16) - Patella tendon graft = 40% (n=8)   Use of KT 1000/2000 (or equivalent) arthrometer device:   - hamstring tendon 25.7% (n=9) - patella tendon 35% (n=7) | RTP decision included RTP time and strength testing. There were some differences in postoperative management of ACLR with hamstring tendon and patella tendon grafts however there were also similarities in ACLR management between the Turkish orthopaedic surgeons and the “ACL study group”. |
| Ebert, J. R.  Webster, K.E.  Edwards, P. K.  Joss, B. K.  D’Alessandro, P.  Janes, G.  Annear, P. | Cross sectional survey study | Specialist knee surgeons   - Australian Knee Society (AKS) | 73 | Update the current perspectives, pre- and post-operative rehabilitation and RTP clearance criteria (including assessment methods) of AKS members. | ACLR - knee | Not described | RTP Time from surgery:   - 42.5% 9-12months - 34.2% 12-18 months - 23.3% 6-9 months   High demand sport RTP clearance criteria:   - 90.4% Functional capacity (e.g. jump and/or hop tests) - 96% Lower limb and trunk mechanics during jumping/landing tasks - 64.4% psychological readiness - 78.1% knee strength - 90.4 time from surgery - 64.4% ROM/laxity - 63% limb asymmetry - 64.4% patient age - 23.3% PRO questionnaires - 8.2% Other e.g. sport specific skill, agility/COD, hip strength, proprioception, effusion   Knee strength and functional capacity for RTP clearance - assessment methods:   - 19.2% In practice rooms with ortho - 58.9% uses a preferred rehab specialist that he/she refers to - 4.1% Instructed patient to go and find a therapist/ practitioner to do assessments - 21.9% assumed the patient’s own therapist/ practitioner is handling RTP - 4.1% I do not formally request the patient undertakes these tests   Percentage of ortho estimating the retear rate of ACLR in past 5 years:   - <2% = 4.1% - <5% = 38.4% - 5-10% = 47.9% - 10-15% = 8.2% - >15% = 0% - No comment/ no idea = 1.4% | Variation in beliefs and practices surrounding rehabilitation and RTP assessment.  Many surgeons refer to specialist rehabilitation practitioners for late-stage rehabilitation and RTP evaluation.  Many surgeons also do not follow up with their patients at the later stages of recovery (Improving this practice could ensure athletes follow good rehabilitation and RTP protocols).  Current ACLR evidence documents the benefit of preoperative and postoperative rehabilitation and the development of RTP assessment criteria consisting of objective strength and functional measures to reduce re-injury rates.  This study highlight the important of ongoing education of orthopaedic surgeons on the importance of rehabilitation and good referral systems for rehabilitation and RTP testing. |
| Erickson B. J.  Harris, J. D.  Fillingham, Y. A.  Frank, R. M.  Bush-Joseph, C. A.  Bach Jr, B. R.  Cole, B. J.  Verma, N. N. | Cross-sectional study | Orthopaedic surgeons   - NFL and NCAA Division I team physicians | 137 | To determine the practice patterns for NFL and NCAA Division I football team  orthopaedic surgeons regarding management of ACL  tears in elite, young, and middle-aged football running backs. | ACL - knee | Elite, young and middle age recreational adults  Regular season game play of a starting running back (all athletes were male) | Criteria:   - 74.5% permit matches after athlete has passed a set of RTP tests (e.g., Vail, single-leg hop). - 56.9% require normal ROM, no pain, full strength, and subjective stability. - 55.47% wait a minimum of 6 months postoperatively to allow their athletes to RTP - 35% require 6 months to have passed since the operation, with a normal examination and passing RTP tests. - 64.23% surgeons do not recommend post-operative braces for their starting running back on RTP | Most surgeons do not recommend a brace on RTP. RTP mostly occurs at a minimum of 6 months postoperatively, with some surgeons requiring a normal examination and normal RTP testing (e.g. single leg hop). |
| Erickson, B. J.  Harris, J. D.  Fillingham, Y. A.  Cvetanovich, G. L.  Bush-Joseph, C.  Cole, B. J.  Bach, B. R.  Verma, N.N. | Survey | Team orthopaedic surgeons   - Olympic Ski/Snowboard - NHL - MLS | 47 | To determine practice patterns relating  to ACL reconstruction in elite athletes. | ACLR - knee | NHL, MLS and Olympic level athletes | Time criteria:   - 60.6% surgeons recommended waiting at least 6 months before RTP - 6.1% recommended waiting at least 9 months - no surgeon recommended waiting at least 12 months - others did not have a specific time frame for RTP.   Other criteria:   - 81.8% recommended RTP after an athlete passed a series of RTS tests (eg, Vail, single-leg hop). - 57.6% recommended waiting until the athlete had full ROM, no pain, full strength, and subjective stability in the knee. | Most of the NHL, MLS, and Olympic team orthopaedic surgeons do not require athletes to brace when they RTP after ACLR. Majority of the surgeons require athletes to complete a series of RTP tests before going back to competition. |
| Farber, J.  Harris, J.D.  Kolstad, K.  McCulloch, P.C. | Cross sectional study | Orthopaedic surgeons   - MLS team physicians | 22 | To identify practice preferences among MLS team orthopaedic surgeons for ACL injuries. | ACL injury - knee | MLS athletes | Assuming the athlete had achieved full ROM, strength, and no effusion.  Time based criteria:  Straight-line run:   - 27% < 3months - 64% 3-4 months - 9% 4-6 months   Begin noncontact ball handling and ball drills   - 37% 2-4 months - 4-6 months 59% - 4.5% 6-8months   RTP without restrictions   - 9% 4-6 months - 82% 6-8 months - 9% 8-10months   What percentage of elite soccer players:  RTP after ACLR?  60-80% = 37%  80-90% = 32%  >90% = 27%  40-60% = 4.5%  RTP at their prior level of performance or greater when compared to pre-injury level?   - 4.5% = 40-60% - 37% = 60-80% - 32% = 80-90% - 27% = >90% | The study did not analyse clinical outcomes post op to allow RTP such as physical exam, radiographs, MRI clinical outcome general health, quality of life, and limb- or knee-specific outcome scores. However there was consensus for the timing of RTP post op - most allowed RTP at 6-8 months post op. |
| Feller, J. A.  Cooper, R.  Webster, K. E. | Cross sectional study  Questionnaire | Orthopaedic surgeons   - AKS | 36 | Orthopaedic surgeons’ preferences for rehabilitation following ACL reconstruction using hamstring and patella grafts. | ACL injury - knee | General population   - RTP depends on type of sport | Time from surgery criteria:  Return to non-contact competitive sport  6-9 months :   - 72% (Patella) -73% (hamstring)   Return to contact competitive sport  6-9months :   - 50% (Hamstring) – 55% (Patella)   9-12 months :   - 27% (Hamstring) - 24% (patella)   Other criteria for RTP   - 25% use strength testing prior to RTS (22% use KT1000/2000 (or similar) arthrometry) - >75% do not use strength tests, arthrometry (aka ROM) or bracing as a prerequisite before RTP. | Post-operative management of hamstring and patella ACL reconstruction is similar. Future research needs to establish the rate of progression through rehabilitation, as this study showed a more conservative approach than other recent reports. Majority of surgeons allow RTP after 6 months. Over 75% do not use strength testing or arthrometry in the decision to RTP. |
| Glattke, K.E.,  Tummala, S.V., Goldberg, B., Menzer, H., Chhabra, A. | Online survey | Surgeons   - Arthroscopy Association of North American (AANA) - American Orthopaedic Society for Sports Medicine (AOSSM) | 46 | The practices of orthopaedic surgeons during the rehabilitation of ACLR | ACLR - knee | General population ranging from children through to geriatrics | RTP protocols:  63% use time and phase  30.4% use phase only  6.5% use time only  84.8% believe quantitative technology is helpful but many believe there are barriers that limit its potential  [40/46 surgeons responded to the unrestricted RTP question]  RTP timeline for unrestricted sports:   - 50% 9 months or later - 42.5% 6-8 months   RTP criteria used to allow unrestricted sports:   - 75% = Physical exam incl ROM, effusion, laxity - 75% = Strength including MMT, HHD, isokinetic testing - 75% = Functional assessment incl single leg hop for distance, single leg vertical hop, cross over hop for distance, triple hop for distance, 6m hop for time, Y-balance - 62.5% = LSI - 42.5% = PROM | The study provided an insight into protocols and modalities used by orthopaedic surgeons when progressing ACLR patients through the rehabilitation phases towards unrestricted RTP. There was variety in rehabilitation patterns and preferences especially in criteria used when progressing patients through each phase. Additionally, surgeons believe in the use of quantitative technology to assist RTP decisions, however there are concerns for the barriers that prevent implementation in clinical practice. |
| Grassi, A.  Vascellari, A.  Combi, A.  Tomaello, L.  Canatas, G.L.  Zaffagnini, S. | Cross sectional study | Orthopaedic surgeons   - Members of Italian Society of Knee, Arthroscopy, Sport, Cartilage and Orthopaedic Technologies (SIGASCOT). | 120 | To assess how the members of the  SIGASCOT  evaluate RTP time and criteria  after ACLR  . | ACLR - knee | General population and professional athletes | Timeline  Sports specific rehab   - 6 months: 89% allow sport specific rehab - 4 months: 31%   Return to training for non-contact sports:   - 6 months: 87% - 4 months: 20%   Return to training for contact sports:   - 6 months: 43% - 6-8 months: 49% - >8Months : 8%   Return to competitive non-contact sports   - 8 months: 92% - 4 months: 48%   Return to competitive contact sports:   - 8 months: 72% - 6 months: 13% - >10 months: 12%   Criteria for RTP:   - Full ROM 77% - Lachmans test 65% - Pivot shift test 65% - 48% used a combination of above 3. - Muscular force evaluation 44%-56% - Functional (hop test) 31% - Proprioceptive tests 29% - Anterior drawer 26% - KT 1000 arthrometer 15% - MRI 12% - Other: 1% kira accelerometer 2% metabolic tests   Validated clinical score for RTP:   - Subjective IKDC 53% - Objective IKDC 50% - Tegner activity scale 19% - Lysholm 15% - KOOS 1% - None 10%   Further criteria used:   - No 89% - If Yes: Psychiatric eval 2%, Metabolic tests 2%, MRI 2%, Isokinetic test 3%, Sport specific tests 3% | Various approaches in RTP after ACLR. Differences seen in time frame for RTT and RTC, and contact and noncontact sports. Testing consisting of ROM, Lachman’s and pivot shift were the most used criteria for RTP decisions. Strength and functional tests are considered less. The survey highlights the need for clear definition for sport activity when assessing RTP outcomes and need for more objective and well-structured RTP criteria that can be implemented into daily clinical practice. |
| Mahnik, A.  Mahnik, S.  Dimnjakovic, D.  Curic, S.  Smoljanovic, T.  Bojanic, I. | Cross sectional study | Orthopaedic and trauma surgeons   - Croatian Orthopaedics and Traumatology Association (COTA) | 39 | Gain an understanding of preferences and opinions of COTA members regarding the treatment of ACL injuries. | ACL | General population | The recommended duration of physiotherapy by 66.67% surgeons was 6-12 wk.  RTP after ACLR allowed by surgeons:  6-9 months : 66.67%  3-6 months : 25.64%  >9 months : others | ACL reconstruction surgery in Croatia correlated with current international recommendations |
| Marshall, N.E.  Keller, R.A  Dines, J.  Bush-Joseph, C.  Limpisvasti, O. | Cross-sectional study | Surgeons | 143 | Evaluation of current trends in immediate postoperative and RTP practices after ACL reconstruction. | ACLR - knee | Not described | Final clearance for athletes RTP:   - 6-9 months: 67% (96/143) - 9-12 months: 27% (39/143) - 3-6 months: 5% (7/143) - >12 months: 1% (1/143)   Brace for RTP:  No: 52% (74/143)  During first year in RTP: 42% (60/143)  Specific sports only: 6% (9/143)  There was no majority consensus for specific metric used to clear athletes to sport as the highest percentage of use in one metric was about one-third.  Most common RTP metric:   - hop test 33% (44/135) - specific time point after surgery 32% (45/139) - One-legged squat test at 25% (34/136) - clearance by physical therapy at 24% (34/140) - general clinical appearance (i.e. effusion, ROM, knee stability, strength) at 22% (30/138) - The Biodex testing was listed as N/A metric in 31% (41/132) and listed as most important in only 9% (12/132). | RTP is allowed after at least 6 months with some form of functional testing and timing from surgery being most important metrics for allowing RTP; however, the variety of metrics used for clearance shows a lack of consistency. |
| McRae, S. M.  Chahal, J.  Leiter, J. R.  Marx, R. G.  MacDonald, P. B. | Online survey cross sectional study | Canadian orthopaedic surgeons | 283 | To conduct a survey of to gain an understanding of  preferences and opinions of Canadian orthopaedic surgeons regarding history and  treatment of ACL injuries. | ACLR - knee | Not described | Time based recommendation for resumption of sport   - 56.3% 6-9months full return to sport - ± 30% recommending 9-12 months   Majority of all surgeons recommended a duration of 6-24 weeks of physiotherapy | Surgeons can evaluate a range of treatment decisions based on the general opinions of their colleagues in Canada. Surgeons and other health practitioners can use this information to educate and advise patients on areas of consensus and areas of uncertainty within Canada, and differences in preference between Canada and other countries. |
| Pandey, V.  Madi, S.  Thonse, C.  Joseph, C.  Rajan, D.  Varughese, J.  Thilak, J.  Jayaprasad, P. S.  Acharya, K.  Ramamurthy, K. G.  Reddy, R.  Amravathi, R.  Rao, S.  Gangavarapu, S.  Srinivas, M.  Jose, S.  Sundararjan, S. R. | Survey questionnaire | Registered sport orthopaedic surgeons in India | 324 | The purpose of the study was to determine the current trends and practices of primary single bundle anatomic ACLR in India and compare it to global trends and practices.  . | ACLR - knee | Athlete/ high demand patient | RTP criteria:  94.4% = Clinical and functional assessment along with the therapist agreement  96.6% do not ask for MRI to determine ACL healing prior to making RTP decision  RTP time:  46.3% = after 6 months  36.3% = after 9 months | The study provided insights into Indian practices and opinions in ACL management. The study explored the evolving trends that have been previously missed in previous ACL studies such as soaking the graft with antibiotic, pre-tensioning, footprint identification, using various portals, extra-articular procedures, and post-operative bracing. This study provides a platform for future research. |
| Petersen, W.  Zantop, T. | Questionnaire study | Arthroscopic surgeons – Germany, Switzerland, Austria | 221 | To determine the factors considered in RTP among experienced arthroscopic surgeons. | ACLR - knee | Professional athletes / competitive level I sports | Majority (50%) said muscle strength training after 4 months  76.6% Begin sport specific rehabilitation after 4 months  48.6% RTT at 4 months, 44% RTT after 6 months  RTP competitive: 35.3 % answered to allow return to competi­tive sports after 6 months. 30.0 % allow RTS after 8 months, 13.3% after 10 months, and 20.2% after 12 months. A total of 63.5 % recommend a time point later than 6 months allowing RTP after ACL reconstruction in the athlete.  RTP criterion:   - a negative Lachman test (81.7 % positive answers) - free ROM (78.4 %) - negative pivot shift (60.1 %). - Anterior drawer was used by 45.4 %. - A proprioception test was used by 43.1 % - muscular strength analysis by 40.8 % - a single-leg hop jump test by 39.0 %, - KT 1000 measurement by 16.1 % - MRI only by 4.1 % - Other: - 11.5 % absence of effusion is NB. - 10.1 % depend on associated injuries, such as cartilage and meniscal inju­ries. - 5.5 % answered that they use the subjective feeling of the patient as criterion to allow RTP. - 4.5 % depend on the sport and on the sport level of the ath­lete (professional, amateur). - 1.4% use a battery of different tests as return to sports criterion.   Use of a clinical score  85.8 % Do not use a clinical score prior to RTP  Out of the 14.2% that use a clinical score, the most frequently clinical used score was the subjective IKDC score (10.6 %), followed by the Lysholm score (8.3 %), the objective IKDC score (7.4 %), and the Tegner activ­ity scale (3.7 %). None of the study participants suggested another clinical score to decide if the athlete can RTP in the open choice field. | This study believed that RTP should not only be timeline based. Although time is an important factor to consider, after a minimum of 6-8 months, test batteries should be used to assist decision-making in RTP clearance. The test battery should include passive stability testing including KT 1000, ROM, no effusion, quadriceps and hamstring strength, balance test, one legged hop tests, and vertical drop jump test. From the battery of tests that are placed into a criteria, a “return to sports score” can be developed. The results of this study should promote discussion about RTP after ACLR. |
| Sherman, S. L.  Calcei, J.  Ray, T.  Magnussen, R. A.  Musahl, V.  Kaeding, C. C.  Clatworthy, M.  Bergfeld, J. A.  Arnold, M. P. | Survey | Orthopaedic surgeons from the ACL study group   - 50% from Europe - 29% USA - 15% Asia Pacific - 6% Latin America, Middle East, New Zealand & Africa   35% Academic practice setting  37% private practice  24% mixed practice  4% retired | 140 | To determine the latest global trends in ACLR by the members of the  ACL Study Group. | ACL - knee | Athletes described as higher level i.e. competitive basketball, soccer, football; moderate level i.e. competitive racquet sports, recreational skiing and lower level athletes i.e. bikers, joggers, swimmers | Return to jogging: 70% only initiate jogging until 3−5 months after primary ACLR.  Timing for initiation of lateral movements and agility training are as follows:   - 5% initiate at 6 weeks - 3 months - 22% at 3−4 months - 49% at 4−6 months - 24% at greater than 6 months. - 83% do not use a functional brace before initiating lateral movements.   Determination of RTP is most commonly  (71%) done using minimum time criteria and functional assessment.  The most common responses for minimal time to RTP after primary ACLR were 6−8 months (44%) and 8−12 months (41%). | This study presented the current trends of the ACL study group. The results provide insight into ACLR management. This is supported with current orthopaedic surgery research. The study facilitates the discussion on ACLR management as it provides insight into the global trends of ACL surgery. |
| Thaler, M.  Khosravi, I.  Putzer, D.  Hirschmann, M. T.  Kort, N.  Tandogan, R. N.  Liebensteiner, M. | Cross-sectional study  Survey | Orthopaedic surgeons   - Members of the European Knee Associates | 120 | To elaborate recommendations for sports participation following total knee arthroplasty (TKA) among the members of the European Knee  Associates (EKA). | Knee - Total knee arthroplasty | Depended on the different sporting codes. 6 weeks; 6-12 weeks; 12 weeks - 6 months; 6months | Type of surgery, time from surgery time frame  0-6 weeks: walking, stair climbing, swimming, aqua fitness, static cycling  6-12 weeks: plus cycling in level ground, yoga  >12 weeks: plus tennis doubles, golf, fitness/weight-lifting, aerobics, hiking, Nordic walking, sailing  >6months: plus mountain biking/incline cycling, table tennis, rowing, canoeing, kayaking, skiing, Tai-Chi.  After 6 months 21 of 47 sports recommended, 20 sports did not reach consensus, 1 sport (squash) not recommended. | 21 of 47 sports recommended after six months but no consensus reached for other sports, especially higher impact or change of direction sports. Surgeons were more inclined to allow RTP if the athlete had experience in the sport before surgery. |
| Vascellari, A.  Grassi, A.  Combi, A.  Tomaello, L.  Canata, G. L.  Zaffagnini, S.  Sigascot Sports Committee | Web based survey | Surgeons (88% orthopaedic surgeons)   - SIGASCOT members | 131 | Report the  management of ACLR, rehabilitation, and compare surgical applications  and rehabilitation approaches of Italian surgeons to  the current approaches of “ACL Study Group”. | ACLR - knee | Not described | 84.3% have a standard rehabilitation programme  Starting specific activities/exercises:  Using a time-based approach  Sports specific rehabilitation  2-4 months 29.8%  4-6 months 56.5%  Skills on sports not requiring contact  2-4 months 21.8%  4-6 months 64.5%  Skills on sports requiring contact  4-6 months 41.6%  6-8 months 48.0%  Return to sports not requiring contact  4-6 months 44.7%  6-8 months 43.9%  Return to sports requiring contact  6-8 months 58.4%  8-10 months 20.8% | This is the first conducted in Italy to report the preferences of Italian surgeons regarding ACLR and rehabilitation. The results obtained from the study revealed a more conservative approach among SIGASCOT members when compared to the current approaches of “ACL Study Group. |
| **Ankle and foot injuries** | | | | | | | | |
| Aguilaniu, A.  Croisier, J. L.  Schwartz, C.  Dardenne, N.  D'Hooghe, P.  Martens, G.  Collin, R.  Kaux, J. F. | Online survey | French speaking physicians   - Sports medicine specialisation include: Sports med education, Physical, rehab and sports med education, Orthopaedic & sports med education, General med and sports med education   Location: Belgium, France & Switzerland | 109  (46% n=50 with specialisation)  54% n=59 (without specialisation) | Practice of a range of assessment criteria for a safe RTP after a lateral ankle sprain. | Lateral ankle sprain | Recreational and professional athletes | This RTP criteria based off the Rehabilitation-Oriented ASsessmenT (ROAST) guidelines for ankle injury and the used quantitative measures:  (quantitative % are relative to the % of respondents that stated criteria that was used)  Pain: used by 90% of physicians. Only 51% use quantitative measures e.g. VAS  Functional tasks: 82% used by physicians; only 31% use objective measure e.g. hop test, balance test, gait and/or sport movement analysis.  Functional instability: used by 73%; only 4% use objective measures such as questionnaires.  ROM: used by 61% physicians; 27% used objective measures e.g. goniometer and/or a measurement tool  Proprioception: 47% physicians use it; 18% use objective tools e.g. Myolux1 and/or goniometer tools and/or arthro-motor.  Mechanical instability: used by 39% physicians; 17% use objective tools e.g. anterior drawer test and/or varus test  Strength: 38% physicians use it in their assessment however 20% use an objective tool e.g. isokinetic and/or Myolux1 and/or hand-held-dynamometer tool  Swelling: 31% physicians use it in their criteria; 24% use an objective tool e.g. figure-of-8  Specialist physicians used pain, functional tasks and ankle ROM more than non-specialist physicians. Specialist physicians assess ankle instability less than non-specialist physicians. Years of experience and percentage of athletes treated did not influence physicians choices. | A large proportion of French speaking physicians are aware of the criteria for pain, ability to engage in functional tasks, functional ankle instability and ankle ROM criteria from ROAST, few are using recommended quantitative measurement tools in practice.  Sports medicine education is a factor that increases the use of some quantitative measurement tools with some criteria. Strength as a criteria was lacking despite strength being considered a modifiable risk factor to reduce the risk of re-injury.  The use of quantitative and qualitative tools could assist physicians making RTP decisions. |
| Beck, J.J., Carpenter, C.M., West, N., Sabatino, M.J., Ellis, H.B. | Survey | Orthopaedic surgeons   - POSNA | 229 | To determine how members of POSNA treat paediatric lateral ankle sprains including ankle sprain and non-displaced Salter Harris type I distal fibular fractures. | Lateral ankle injuries – ankle sprain and salter Harris type 1 | Paediatric patients (age not specified) | **RTP timelines**  Ankle sprains:   - 1-2 weeks: 9.5% - 3-4 weeks: 60.2% - 5-6 weeks: 22.9% - 7-8 weeks: 4% - >8 weeks: 3.5%   Salter Harris-1   - 1-2 weeks: 0.5% - 3-4 weeks: 38.6% - 5-6 weeks: 48% - 7-8 weeks: 10.4% - >8 weeks: 2.5%   Influencing factors related to surgeons:   - Age - Sex - Experience - In terms of RTP, main variability seen between surgeons from academic institutions vs private or solo practice | This study found significant variability in treatment of ankle sprain injuries and Salter Harris 1 distal fibular fractures. The age, sex and experience of surgeons created the greatest variability in practice. Ankle sprains were more like referred to physiotherapy and saw a quicker RTP in comparison to Salter Harris-1 which was treated with cast immobilisation. Complications included problems with cast treatment, continued pain/reflex sympathetic dystrophy, non-union, growth arrest, recurrent fracture and infection due to injury, treatment, and neglected treatment after ankle sprains and Salter Harris-1. |
| Dams, O.C.  Van den Akker-Scheek, I.  Diercks, R.L.  Wendt, K.W.  Zwerver, J.  Reininga, I.H.F | Cross sectional study | Orthopaedic surgeons and trauma surgeons | 91 | This study aimed to investigate the management of Achilles tendon ruptures by orthopaedic surgeons  and trauma surgeons in the Netherlands. | Achilles tendon injury | Not described | (Study group was asked what time they advise for RTP, not what criteria they use in the decision for RTP.)  Majority of trauma surgeons (about 50%) recommended RTP for both surgical and non-surgical treatment to be 10-14 weeks post injury or op.  Orthopaedic surgeons recommended waiting a longer period before RTP for both surgical and non-surgical responses compared to trauma surgeons. Similar responses (about 25%) at 14-18 weeks and (25%) >26 weeks post injury or op. | Diagnosis and treatment preferences for Achilles rupture in the Netherlands are based on clinical factors. There is only partial agreement due to limited available scientific evidence and guidelines.  There was consensus for management however there was a lack of consensus for time RTP when comparing orthopaedic surgeons with trauma surgeons.  The study encourages the use of existing evidence, the development of clinical guidelines for primary treatment of different patient populations and the application of evidence-based rehabilitation principles. |
| Vertullo, C.  Nunley, J. A. | Questionnaire | Orthopaedic surgeons - Members of the American Orthopaedic Foot and Ankle Society | 103 | Explores the opinions on RTP  after ankle arthrodesis. Details the type of arthrodesis and type of  sports of professional athletes successfully  returned to were reported by surgeons and  trainers. | Ankle and foot arthrodesis | Professional athlete returning to sport = NBA basketball, NFL and CFL football, 1^st^ division European soccer | RTP depended on the type of sport   - respondents would allow patients to return to golf after IPJ arthrodesis of the great toe - lowest rate of allowance was 11% for football players, basketball players and soccer players returning to their sport after ankle arthrodesis. - 12% of respondents allowed RTS irrespective of the arthrodesis - 88% selectively allowed their patients to return based on arthrodesis and sport. - 4.8% would allow return to any sport provided the athlete was able to do the sport, but doubted that patients could play some sports with a hindfoot or ankle arthrodesis. - Golf achieved the highest allowance rate, followed by skiing. | Variety in RTP advise was due to the range of intensity at which different sports are played. Surgeons are supportive of a selective RTP policy as long as the athlete is empowered to make the best decision and knows the consequences of RTP. |
| Yokoe, T.  Tajima, T.  Yamaguchi, N.  Morita, Y.  Chosa, E. | Questionnaire survey | Orthopaedic surgeons working in Miyazaki, Japan | 129 | This study evaluated the current clinical practice of general orthopaedic surgeons treating lateral ankle sprains. | Lateral ankle sprain | Athletes - generalised | RTP advice:  74.4% of surgeons in struct athletes to wear an ankle supporter during sports activity after lateral ankle sprain  RTP criteria:   1. *Absence of pain 2. Absence of instability 3. Time after injury 4. Full ankle ROM 5. No swelling   65.9% of surgeons only use one criteria to determine RTP. *45.7% use absence of pain | The current management of lateral ankle sprains by orthopaedic surgeons in Miyazaki, Japan were reported in this study. When performing plain radiographs, the Ottawa ankle rules were not considered by majority of clinicians. Rehabilitation was also only performed by less than 60% of patients who sustained a lateral ankle sprain. For RTP decisions in athletes with lateral ankle sprain, no objective criteria was used. |

### Physiotherapists

| **Author** | **Type of study & data collection** | **Participant’s profession** | **Sample size** | **Purpose** | **Injury location** | **RTP level – patients activity level profile** | **RTP practices, attitudes and behaviours** | **Conclusions** |
| --- | --- | --- | --- | --- | --- | --- | --- | --- |
| **RTP practices** | | | | | | | | |
| Silva, Anderson A  Bittencourt, Natália FN  Mendonça, Luciana M  Tirado, Marcella G  Sampaio, Rosana F  Fonseca, Sérgio T | Cross sectional survey | 49 Brazilian physical therapists   - 27 work in soccer clubs, 22 work in volleyball - 44 male; 5 female - Specialisation: - 47% sports physical therapy - 15% acupuncture - 7% trauma & orthopaedic - 31% others | 49 | To analyse the profile of Brazilian physical therapists working with professional soccer and volleyball teams | General RTP practice | Soccer & Volleyball | Final RTP decision-making responsibility:  SDM between physician and physiotherapist: 74.5% of the cases  SDM participation between athlete and physiotherapist 63.6%  Only physician: 14.5%  Only physical therapist: 10.9%  RTP clearance criteria:  “subjective of the health team”: 33%  “excellent physical, technical and functional capacity”: 22%  “pathophysiological criteria”: 20%  “needs of the club”: 20%  Tests or evaluations for RTP:   - 67.3% “always” performed - 27.3% “almost always” - 5.5% “sometimes”   RTP (e.g. functional activities in soccer and volleyball) performed by:   - physical therapists with the physical educator (70.9%) - physical therapist alone (27.3%). - Other interactions (1.8%)   Physical therapists’ areas of activity:  100% RTP  92.7% prevention  98% functional rehabilitation  87.3% emergency care  Physiotherapists decisions in sports team were restricted by the directors, the coach and the physician. The physician restricts physiotherapists decisions the most. | Sports physiotherapists are active within the MDT. Physiotherapists are often involved in performance of injured athletes, functional rehabilitation, RTP decision. This study shows the relationship of the physiotherapist in rehabilitation process and RTP within the Brazilian sport community. |
| **Upper extremity/Shoulder injuries** | | | | | | | | |
| Brindisino, Fabrizio  De Santis, Andrea  Rossettini, Giacomo  Pellicciari, Leonardo  Filipponi, Marco  Rollo, Giuseppe  Gibson, Jo | Web-based observational cross-sectional study | Physiotherapists based in Italy | 1160  - 631 Male  - 1160  Female | To determine if specific post graduate training impacts evidence based practice. | Rotator cuff repair – shoulder | Not described | The advice the clinicians give clients post RC repair:  Starting light duty work e.g. computer work etc:  9% 3-4 week  33% 4-6 weeks  36% 7-12 weeks  18% 13-24 week  Heavy work e.g. working in a warehouse  15% 7-12 weeks  53% 13-24 week  29% >24 week  Non overhead sports  4% 4-6 weeks  15% 7-12 weeks  52% 13-24 week  28% >24 weeks  Overhead sports  4% 7-12 week  26% 13-24 week  68% >24 weeks  The follow up evaluation modalities:  PROMs are used by 45.6% of participants; mobility tests are used by 19.5%; muscle strength tests used by 16.0%; personal interviews 14.9% and telephone interviews used by 3.1%. | Italian physiotherapists’ management of postoperative RC surgery patients is in-line with the published guidelines by the American Society of Shoulder and Elbow Therapists and other UK surveys. Similarities is specific to RC post-operative immobilisation periods, active and passive mobilisation and RTP activities. There is less practice consistency with regards to exercise, patient follow up and referral. |
| Brindisino, F.  Lorusso, M.  Usai, M.  Pellicciari, L.  Marruganti, S.  Salomon, M. | survey | Physiotherapists working in Italy | 607 | The first aim of this study is to investigate the Italian physiotherapists (PTs) clinical practice in the management of patients with TSA and RTSA and to compare it with the best evidence available in the literature. The second purpose of this study is to assess any existing difference between the survey answers and the different sample subgroups. | Total shoulder arthroplasty (TSA) & Reverse total shoulder arthroplasty (RTSA) | Not described | In your clinical practice, following TSA surgery, when do you expect the patient to be able to return to sport? (in details: involving the upper limb, non-contact sport and non-throwing sport) (Q21)   - 6-12 weeks 3.1% - 13–24 weeks 27.5% 7 months–1 year 56.0% - Over a year 13.3%   In your clinical practice, following RTSA surgery, when do you expect the patient to be able to return to sport? (in details: involving the upper limb, non-contact sport and non-throwing sport) (Q30)   - 6-12 weeks 4.0% - 13–24 weeks 19.6% 7 months–1 year 46.6% - Over a year 29.8% | The clinical practice of Italian PTs in the rehabilitation of patients with TSA and RTSA effectively reflects the indications of the literature as far as the strengthening of the main muscle groups and the prevention of movements which may result in a dislocation are concerned. Conversely, there are still substantial differences when considering the passive and active joint mobilization starting time and progression, muscle strengthening techniques (timing and dosage) and the return to sport management (timing). These disagreement aspects actually reflect the uncertainty of current literature degree of knowledge on post-surgical rehabilitation for shoulder prosthesis in the rehabilitation field. |
| Gauthier, M. L.  Unverzagt, C. A.  Mendonça, L. M.  Seitz, A. L. | survey | Physical therapists   - With port specialisation (26.9%) - without sports specialisation (73.1%) | 498 | The purpose of this study was to explore the reported frequency of physical performance testing for return to sport readiness by physical therapists treating athletes with upper extremity injuries and to identify potential barriers that may limit use of these tests. A secondary aim was to compare practice patterns of clinicians with sports physical therapy specialty certification to clinicians without. | Upper extremity injuries | athletes | ICF framework used to make RTP decision  (% of all PT)   - Contextual factors   - 97.8% Timeline from injury   - 93.2% Patient self-reported readiness   - 80.9% Patient reported outcome measures - Impairments, body structure and function   - 98.6% ROM   - 82.5% Manual muscle testing   - 55.6% Hand held dynamometry   - 19.7% Isokinetic testing   - 56.8% proprioception - Activity restrictions   - 23.1% Seated shotput test   - 45.8% Closed kinetic chain upper extremity stability test   - 42.6% Upper quarter Y-balance test   - 18.7% Timed push up test   - 9.0% One arm hop test   - 12.0% Athletic shoulder test   - 10.8% Shoulder arm return to sport test   “Almost all participants stated that they tailored their battery of tests based on the sport to which the athlete is returning, and expressed the desire to use more physical performance tests in their re- turn to sport assessments.”  Barriers to the use of physical performance tests   - 44.2% Lack of time - 51.8% Lack of equipment - 50.2% Lack of understanding of literature supporting PPTs - 38.0% Perceived lack of research supporting use of PPTs - 19.5% Lack of referral source interest - 7.6% Lack of patient interest   Less than 25% of participants reported that their patients consistently passed all physical performance tests before discharge. (36.6% of sports specialists and 20.6% of non-specialists)  Barriers to patients being discharged before full physical performance:   - 68.9% insurance visit limitation - 67.9% patient self-discharge - 54.5% financial reasons - 52.8% clearance from another healthcare provider - 34.5% external pressure for the athlete to return to sport | The results of this survey suggest that the vast majority of clinicians are not using physical performance tests in making return to sport decisions for athletes with upper extremity injuries. By omitting these tests, and neglecting to assess an athlete’s activity limitations, physical therapists are “missing the forest for the trees” and may be returning athletes to sport before full physical performance. While sports specialist clinicians are more likely to utilize these tests to assess an athlete’s activity limitations, all participants highlighted a perceived lack of supporting research, a lack of understanding of current research, and a lack of time and equipment to perform these tests in the clinic. Strategies recommended to overcome these barriers include increasing the emphasis on assessing activity limitations for return to sport clinical decision making in entry-level and post-professional education, reducing the barriers and challenges associated with accessing post-professional education, and developing clinical guidelines for the utilization of physical performance tests in athletes with upper extremity injuries. |
| **Hamstring injuries** | | | | | | | | |
| Nasser, A. M.  Pizzari, T.  Grimaldi, A.  Vicenzino, B.  Rio, E.  Semciw, A. I. | Semi-structured interviews- qualitative study | Expert physiotherapists  -Published in the topic area and/or had extensive clinical experience in treating patients with PHT.  -Registered physiotherapists with experience treating people who have PHT  -Hold a Master’s degree or Doctor of Philosophy  have a minimum of 10 years’ experience. | 13 | Physiotherapists’ opinions on assessment, management  and prevention of proximal hamstring tendinopathy (PHT) | Proximal hamstring tendinopathy | Athlete | Readiness to RTP physiotherapists perceived:  Key points:   - Assess hamstring strength and symptom response to loading tasks e.g. HHD 80-90% of unaffected side, pain free isometric muscle tests, pain free functional movements - Most athletes RTP with symptoms - Individuals in team sports can often continue to compete while recovering - Strength and conditioning exercises that required larger ranges of hip flexion, such as deadlift, leg press is only reintroduced if necessary once full RTP is reached and stable   RTP monitoring:  Key points:   - VISA-H is rarely used - Self-monitoring using a numbered rating scale in load based tests such as arabesque, single-leg bridge) - If there is a change in sitting pain 24 hours after sport/activity - Isometric strength using a knee flexion dynamometry - TAMPA scale for psychological readiness to RTP   Study indicated that due to the nature of competitive sport, RTP after PHT is often rushed  and rehabilitation is incomplete.  Preventing recurrence key points:   - Running technique - Strength training for kinetic chain - Hamstring strength - Education regarding load management   After breaks from sport preventing re-injury is important. There is a risk of re-injury due to the sudden spike in workload. Physiotherapists use objective measures for re-assessment such as:   - strength testing with HHD or in gym-based exercises | Using the combination of a progressive rehabilitation program, educating patients understanding of pain and tendon load and allowing self-monitoring and progression are vital in PHT management and prevention of  Re-injury. Physiotherapists perceived passive management strategies to be ineffective in the management of PHT. |
| Valente, H. G.  Oliveira, R. R.  Baroni, B. M. | Online survey | Brazilian physical therapists | 62 | To describe perceptions and practices of physical therapists from elite men's football clubs on the management of athletes with hamstring strain injury (HSI). | Hamstring strain injury (HIS) | Elite men’s football athletes (Brazilian Men’s Football Championship) | Do the club's physical therapists follow a standard protocol for RTP of athletes with HSI?   - No (11.3%) - Yes (88.7%)   Do you adopt specific criteria to release athletes following HSI to return to training with the team (or with the staff responsible for transition)?   - No 3.2% - Yes 96.8%   If yes, which one(s)?   - Muscle strength (71%) - Sprints/high speed running (58.1%) - Intense actions (e.g. accelerations) (43.5%) - Pain (43.5%) - External workload (GPS metrics) (32.3%) - Range of motion (27.4%) - Functional tests (e.g. hops, H-test, SLHB) (27.4%) - Imaging exam (22.6%) - Time since injury event (3.2%) | The present study allowed the sports physical therapy community to become aware of the approaches usually adopted for management of athletes with HSI who play in the highest level of Brazilian men's football. Despite the heterogeneity of choices regarding assessment practices, all respondents use imaging exams, adopt injury classification scales, and evaluate aspects related to pain, range of motion, muscle strength, and functional status of athletes with HSI. Rehabilitation programs are usually divided into 3 to 4 phases. Electrophysical agents, manual therapy, stretching, strengthening exercises (including eccentrics), lumbopelvic stabilization exercises and exercises that mimic the functional demands of football are used by vast majority of respondents. Muscle strength was the most reported RTP criterion. |
| **Knee injuries** | | | | | | | | |
| Alshehri, Y. S.  Aljohani, M. M. A.  Alzahrani, H.  Alzhrani, M.  Alkhathami, K. M.  Alshahrani, A.  Khaled, O. A. | Survey | Physical therapists | 177 | To investigate the current rehabilitation practices and RTP criteria after ACLR among physical therapists in Saudi Arabia | ACL | Active population | 61% PT (n=108) allow RTS at 6 to 9 months post-ACLR if they were satisfied with their patient’s progress and physical capacity.  RTP clearance criteria:   - 86.4% - functional capacity - 78% on knee range of motion and/or laxity - 72.9% on knee strength - 62.7% on lower limb and trunk mechanics during jumping/landing tasks - 57.6% on time from surgery.   If knee strength important to clear RTP:   - 39% reported using manual muscle testing to evaluate knee strength - 15.3% who used handheld dynamometry - 15.3% who felt strength is important, but they do not have access to necessary equipment (and/ or do not feel manual testing methods are accurate enough) so they refer on to someone who can provide such an evaluation for them.   For the assessment of lower-limb functional capacity   - 61% of the respondents reported using single-limb hop for distance to assess lower-limb functional capacity - 44.1% 6-m timed hop test - 44.1% single-limb vertical hop - 42.4% star excursion and/or Y-balance test - 40.7% triple crossover hop for distance - 33.9% triple hop for distance - 27.1% at least 2 hop tests battery   Decision making: 64.4% of the therapists reported that in their work area, both orthopaedic and rehabilitation specialists engaged in the decision making to determine patients’ readiness to return to unrestricted sports. | Physical therapists in Saudi Arabia demonstrated variations in their current rehabilitation practices and RTS criteria after ACLR. About two-thirds of the therapists preferred working with their patients immediately following the surgery. More than half of the therapists believe that preoperative rehabilitation is important to the overall postoperative outcomes. The current perspective of the therapists should be updated with current evidence-based regarding the RTS timeframe, the importance of assessing psychological readiness to RTS, and the use of objective measurements when evaluating knee strength. |
| Aquino, C.F.  Ocarino, J. M.  Cardosos, V. A.  Resende, R. A.  Souza, T. R.  Rabelo, L. M.  Fonseca, S. T. | Cross sectional study – electronic survey | Brazilian Physical therapists  19.8% Bachelors degree  62.0% specialisation course  13.9 Masters  4.3 Doctoral  Certification in sports PT 19.4% (80.6% no)  Certification in orthopaedic PT 13.2% (86.8% no) | 439 | To describe the treatment of ACLR and RTP decision-making criteria In Brazilian physiotherapists. To investigate factors associated with RTP clearance. | ACLR - knee | 44.4% Recreational athletes, 39.6% amateur athletes, 9.8% professional athletes, 6.2% sedentary athletes | Indicated decision-maker: 62.1% multidisciplinary team. 25.1% physical therapists. 12.8% orthopaedic surgeon.  Time from surgery:  41% 6-7 months  21.9% >8months  21.2% 4-5 months  15.3% 1-3 months  RTP clearance: Majority >50% related to physical factors (quad, hamstring and hip muscle strength and ROM. 6.4% recommended use of all 5 measures in literature (quad and hamstring strength, functional test performance, functional questionnaire score and psychological readiness).  Results breakdown:  75.1% quad strength  68.5% hamstring strength  66.1% hip strength  65.3% ROM  49.6% movement pattern  46.4% time from surgery  42.8% performance in functional tests  31.8% Clinical orthopaedic tests  19.1% Psychological readiness  16.6% score in functional scales (PROMs)  6.1% no criteria  The only factor that was associated with the use of all recommended measures/criteria was having a specialist certification in Sports Physical Therapy  <19% have masters of doctoral degree. 62% have done a specialisation course however majority (80.6% & (86.8%) do not have certification in either Sports physical therapy or orthopaedic physical therapy. | Less than 10% of Brazilian physical therapists use all of the criteria recommended in literature to determine RTP readiness after ACLR. Most practitioners use measures related to physical elements in RTP decision-making process (e.g., muscle strength and knee ROM) and a small number of physical therapists use PROMs and psychological questionnaires. The use of recommended criteria to determine RTP was associated with Sports Physical Therapy certification, but not years of experience or academic degree. |
| Fausett, W. A.  Reid, D. A.  Larmer, P. J. | Online cross sectional survey | Physiotherapists in New Zealand | 318 | New Zealand physiotherapists clinical beliefs and practices of pre and post-surgical rehabilitation and RTP criteria following ACLR. | ACLR - knee | Generalised population | RTP timeline incl. high demand, pivoting and contact sports   - 11% 6-9 months - 63.2% 9-12 months - 24.2% 12-18 months - 1.3% >18 months - 0.3% don’t encourage athlete to return to high risk sports   RTP criteria most commonly considered:  98.1% Functional capacity  92.5% lower limb/trunk mechanics during functional tasks  90.3% knee strength  89.3% psychological readiness  75.2% time from surgery  Other criteria reported include: PRO measures, thigh girth, ROM/laxity, RTP test batteries or sport specific tasks, agility/proprioception, gym maximal tests, cardiovascular fitness, surgeon clearance, combination of multiple functional tests  Other factors for consideration: Age of patient | New Zealand physiotherapists have varied rehabilitation and RTP practices and beliefs following ACLR. Rehabilitation pre and post ACLR is recognised well and although the beliefs are in line with current recommendations, it is unclear if this is consistent in clinical practice. The time to start post-operative rehabilitation and the frequency of treatment visits varied due to many factors. Future research should include the discrepancies seen between therapists beliefs and the use of objective treatment data. Barriers that delay the start of post-operative rehabilitation need understanding and improving the use of clinical practice guidelines. Lastly, improving the access to appropriate methods of assessment for knee strength and RTP testing following ACLR. |
| Greenberg, E. M.  Greenberg, E.T.  Albaugh, J.  Storey, E.  Ganley, T.J. | Cross-sectional study | Physical Therapists in America  52.5% American Board of Physical Therapy speciality in either orthopaedic clinical specialist or sports clinical therapist. | 1074 | To understand the practice among members of the American Physical Therapy Association, regarding rehabilitation decision-making and criteria to guide activity progression fol­lowing ACLR. | ACLR - knee | Young athletes following ACLR | “Are there specific physical tests, examination findings, or criteria that you utilize in order to assist in the de­cision to progress to [jogging, modified sports activity, or unrestricted return to sports]?”p. 804  % = of participants  Criteria to initiate jogging:  91.6% knee strength  86.9% functional/balance tests  80.3% ROM  70.6% knee effusion  Criteria to initiate modified sports activity:  80% knee strength  82.5% functional/balance tests  61.9% ROM  59.6% knee effusion  54.7% said no further additional testing needed to progress patients at return to jogging or modified sports stage.  More than 80% of physiotherapists felt that a multifactor approach was important in decision-making after ACLR, using physical measures such as strength, lower extremity func­tion, and dynamic stability.  Time based criteria:  *Modified sports activity (eg, agility and coordination drills)*  50.1% 4-5 months  31.4% 6-7 months.  *Progression to unrestricted sports*  23.4% 6-7 months  14.7% 7-8 months  39.8% 9 -12 months  RTP common assessment method:  Testing knee strength: MMT 74.3%  Isometric dynamometry or HHD or rep max test 54.9%. 56.1% use only MMT whilst 43.9 use together with other objective measures. (> 90% LSI)  Functional testing:  89.2% single limb hop tests  48.8% Y balance test or star excursion test (  39.5%.drop vertical jump  7.4% Vail sport test  10.6% LESS  31.2% FMS  12.1% Balance assessment tool  PROMs used by 45.3% physical therapists to progress into modified sport: Lower extremity functional scale was use by the majority (39.2%).  80.1% use shared decision-making to progress rehab/RTP between orthopaedic surgeon and physical therapist.  41.1% physical therapists in favour of using functional bracing on RTP  74.9% physical therapists recommended injury prevention programs after ACLR:  - Prevent injury & enhance performance 31.3%  - FIFA 11+ 21.4%  - Individually adapted program 29.7% | The difficulty in predicting time line of RTP following ACLR likely reflects the complexity of decision-making in the later phases of reha­bilitation, such as type of sport, patient-specific factors and variability in guidelines.  The results indicate that there is a large degree of variation in rehabilitation pro­gression among physiotherapists, especially timing of activity progression, strength assessment, and use of PROMs. This pattern of inconsistency esca­lated as the time from surgery increased. |
| Kaye, J. A.  Spence, D.  Alexanders, J. | Qualitative research – semi structured interviews | Physiotherapy students | 10 | The aim of the study was to explore student physiotherapist perceptions and experiences of using a biopsychosocial approach within ACL rehabilitation. | ACL | Biopsychosocial framework in general practice in ACL rehabilitation | Physiotherapy students addressed different aspects of biopsychosocial approach  Training:   - lack of psychological raining and the application into practice - Students could identify a person displaying psychological symptoms but lacked understanding of the wider social complexities   Understanding the biopsychosocial approach   - students treated patients using a biopsychosocial approach and being patient-centred was the most prevalent feature in the approach. - Students understood the importance psychology within ACL rehab but felt that their education neglected the psychological state of the patient   Barriers   - Time and patient expectations - Limited time to assess and treat patient holistically - Building relationships and rapport with their patients is important to engagement and compliance in rehabilitation | This study identified student physiotherapist perceptions and opinions toward adopting a biopsychosocial approach within ACL rehabilitation and considered inadequacies of the research on physiotherapists’ education, knowledge and application to practice when providing rehabilitation for patients pre and post-ACL injury. There appears to be a lack of taught material on how to apply these approaches practically within a rehabilitation setting, and confidence in these tools when addressing all aspects of a patients care. |
| Korakakis, V.  Kotsifaki, A.  Korakaki, A.  Karanasios, S.  Whiteley, R. | Online survey | Greek physiotherapists | 538 | To understand physiotherapists perspectives and clinical practice in the rehabilitation after ACLR | ACLR- knee | Not described | 74.1% perform on-field rehabilitation prior to RTP  RTP time:  11.3% <6months  58.7% 6-9 months  24.5% 9-12 months  5.5% 12-18months  Criteria used for RTP:   - Double leg functional tests 82.3% - Single leg functional tests 95.4% - Laxity tests 53.8% - PROMs (ACL-RSI, KOOS, IKDC, Tampa scale of kinesiophobia) 19.8% - Muscle mass 55.2% - Patient (psychological) readiness 28.8% - Strength 74.7% - Signs & symptoms 49.5% - Age of patient 24.7% - ROM 51.1% - Functional capacity 73.6% - Other   83.9% of physiotherapists recommend injury prevention programs after ACLR (FIFA 11+, Prevent Injury and Enhance Performance Program, Sportmetrics ACL intervention Training program, individually adapted program)  Influencing factors: Experience, ACLR volume | A large variability in measures and objective criteria is used by physiotherapists for clinical decision making especially depending on practice experience and ACLR volume. The findings in this study are similar to other reports worldwide. ACLR rehabilitation practices are seemingly not aligned with latest research and guidelines. It is important that future research focuses on understanding the barriers that prevent clinicians from implementing research into practice, improving ways of spreading knowledge and promoting the usability of latest research into practice. |
| Mendonca, L. D. M.  Bittencourt, N. F. N.  Alves, L. E. M.  Resende, R. A.  Serrão, F. V. | Cross sectional study | Brazilian Physical Therapists  With speciality 80% (n=98)  Without speciality 20% (n=23) | 121 | Identify the interventions used by Brazilian physiotherapists in treatment, prevention and RTP criteria used in patella tendinopathy in athletes. | Patella tendinopathy - knee | Not described | RTP criteria options included: pain, function, the Victorian Institute of Sport Assessment --- Patella (VISA-P) score, performance in a functional test and others.  Criteria used to RTP:  Pain = 3.3%  Function = 4.9%  Functional test = 3.3%  Pain + function = 21.5%  Pain + functional test = 4.9%  Function + functional test = 14.8%  All = 44.6%  Cited VISA-P = 7.4%  No answer = 2.4%  *Most cited criteria to define RTP was the combination of pain intensity, function and performance in a functional test  Top 3 functional tests:   - hop test (*n* = 28; 23.2%) - single-leg decline squat/single-leg squat (*n* = 13; 10.8%) - step-down test (*n* = 8; 6.6%). | RTP for athletes with patella tendinopathy can be slow and depends on severity of pain, dysfunction and the quality of the rehabilitation. The results show Brazilian physiotherapists choose a combination of pain, function, and functional tests in RTP decisions.  Although the severity of patella tendinopathy in athletes can be used by the VISA-P as it assesses the presence of pain and dysfunction, only 10% of the participants cited VISA-P as a criterion to clear RTP. The main functional tests used by physiotherapists - hop test, single-leg decline squat/single-leg squat and step-down test - are standardized and indicated in patella tendinopathy RTP. |
| Pulver, M.  Hilfiker, R.  Bizzini, M.  Mathieu, N.  Meyer, S.  Allet, L. | Online survey | Swiss physiotherapists | 247 | To investigate current clinical practice of Swiss physiotherapists treating patients with ACLR from pre-operative rehabilitation until RTS. We assessed optimisation strategies in daily practice and the perceived barriers to these optimisations, and evaluated whether there was a relevant difference in clinical practice for physiotherapists with post-graduate certification in sports physiotherapy or deep clinical experience and those without such experience. | ACLR | Active population | Criteria considered as important for RTS   - Hop test(s) (91.1%)   - Single hop for distance (81.8%)   - Side hop test (71.6%)   - Triple hop test for distance (69.3%)   - Cross-over hop test for distance (48%)   - Single limb vertical hop (24.9%)   - 6m timed hop test (17.3%)   - Other (10.7%) - Strength test(s) (84.6%)   - Quadriceps (97.1%)     - Leg press (62.6%)     - Squat test (43.3%)     - Knee extension seated test (36.5%)   - Hamstrings (90.9%)     - Seated leg vurl (46.3%)     - Nordic Hamstring exercise (37.9%)     - Isokinetic test performed elsewhere (33.7%) - Time (number of months post op) (62.8%)   - +-9months med and median - Sport specific test(s) (59.5%)   - did not perform them 60.5%   - sport specific gestures/demands 20.4%   - on-field testing 4.8% - Balance test(s) (55.5%)   - Y-balance (80.3%)   - SEBT (28.5%)   - Other (5.8%) - Agility test(s) (46.6%) - Psychological test(s) (39.3%)   - ACL-RSI 78.4% - Other (2.4%) - I do not consider any criteria (0.4%)   Treatment and RTS barriers/limiting factors   - Time available (35.5%) - Limitation of prescriptions (surgeon/insurances) (35.5%) - Tariffication system (31.3%) - Infrastructure (less space or gym equipment) (24.7%) - Testing not paid (13.3%) - Administrative work not paid (7.8%) - Lack of equipment with testing (7.2%) - Interprofessional collaboration (4.8%) - Gym not paid (3%) - Patient compliance (2.4%) - No more precision (1.2%) - No group therapy (1.2%) | In Switzerland, large differences were found in current clinical practice for ACLR rehabilitation which some practices not supported with evidence. While most physiotherapists integrate open kinetic chain quadriceps exercise during rehabilitation, only a minority of physiotherapists treat patients pre-operatively, use PROMs during rehabilitation, or consider psychological aspects to be important for RTS. This suggests that the transfer of best evidence into practice remains a challenge. Limitations and barriers to the implementation of ACLR evidenced-based rehabilitation have been reported. Important limiting factors include the tariffication system, the time available for treatment, and the limited number of prescriptions available. There is a call for more pre-operative treatment, longer rehabilitation if necessary, and the use of criteria- based rehabilitation as standard. Further studies should focus on implementation strategies to put best evidence into practice and redefine the clinical process around ACLR rehabilitation in Switzerland in order to improve the overall quality of patient care. |
| Tondelli, E.  Feroldi, A.  García, F.  Meza, F.  Dingenen, B. | Online survey | Argentina Physical Therapists (PT) | 619 | To investigate the current clinical practice regarding pre- and post-surgical rehabilitation and return to sport (RTS) criteria following anterior cruciate ligament reconstruction (ACLR). | ACLR | Amateur to professional athletes | RTP decision  54.3% perform RTR and RTS assessment in clinical practice  Who decides:  4.2% Surgeon  16.7% PT  79.2% multidisciplinary  RTP criteria  47.8% Functional capacity  43.6% Muscle strength  38% Time from surgery  33.4% Lower limb and trunk mechanics in jumps  31.3% Training with team  26.7% ROM and knee laxity  23.7% Thigh circumference difference  16.8% Agility test  12.4% Self-reported functional questionnaires  12.3% Age  11 Psychological readiness  3.1% Other  Use of strength assessments:  16.6% Extrapolate from other measures such as hop capacity  15.7% Handheld dynamometry  15.3% Manual muscle testing  14.7% No access to assess  5.7% Isokinetic dynamometry  4.4% Not important  Use of lower extremity functional capacity assessment:  29.6% Single leg vertical jump  26% Single leg hop for distance  24.4% Star excursion balance test or Y-balance test  22.1% Tripe hope for distance test  22% Side hop  17.4% Hop test battery  15.5% Crossover triple hop  10% Medial triple hop  9.2% 6m timed hop test  5.5% Not important  3.7% Other such as FIFA 11+  Time from surgery:  RTR  41.7% 3-4months  24.1% 4-5 months  12.8% 2-3 months  10.7% do not consider time  7.7% >6 months  2.1% other criteria  0.9% 1-2 months  RTS  45.8% 9-12 months  43.5% 6-9 months  7.4% less than 6 months  3.3% after a year  Use of psychological self-reported functionality and psychological readiness questionnaires:  59.9% do not use  1.6% do not know them  28.7% don’t consider them important in RTS  5.8% use ACL-RSI  3.4% IKDC  1.4% Lower extremity functional scale (LEFS)  2.1% Tampa scale of kinesiophobia (TSK)  1.4% knee injury and Osteoathritis outcome score (KOOS) | This study reported substantial variability in clinical practice of PTs regarding pre- and post-surgical rehabilitation and RTS criteria following ACLR. Current rehabilitation practices following ACLR in Argentina are largely not aligned with contemporary evidence and scientific guidelines. Particularly, in the use of preoperative physical therapy, ROM and strength assessment, and the criteria used for RTR and RTS. Future research should be directed at understanding the barriers faced by Argentinian PTs in implementing the findings of this research into their practice, improving the dissemination of scientific knowledge and their implementation in clinical practice. |
| van Melick, N.  Hoogeboom, T. J.  Pronk, Y.  Rutten, B.  van Tienen, T. G.  Nijhuis-van der Sanden, M. W. G.  van Cingel, R. E. H. | Prospective observational cohort study | Physiotheraists in the Netherlands   - including specialised sports physiotherapists | 158 athletes  108 physical therapist (49 registered sports physical therapists) | The purpose of the study was to analyse if physical therapists adhere to ACLR practice guideline with regards to RTP criteria, testing and decisions. To explore differences in adherence between physical therapists specialised in sports versus non specialised physical therapists. | ACLR | Pivoting athletes  54 females and 104 males, mean age 24±6 years | RTP 12±3 months after surgery  Out of 158 athletes, 69 (44%) had per­formed the RTP measurements with their own physiotherapist. 56% of pivoting athletes were cleared for RTP by their physiotherapist without using standardised criteria  Of the 69 athletes tested by their own physiotherapist:   - 23% met all RTP criteria compared to 10% of the athletes who were not tested at all by their physiotherapist   Physio-related factors  Qualifications: Of the athletes rehabilitating with a sports physiotherapist, 52% had been tested by their own sports specific physical therapist compared to 34% of the athletes rehabilitating with a non-sports physiotherapist.  RTP measurement:  Quantitative measurements:   - Isometric knee extensor strength - Isometric knee flexor strength - Eccentric knee flexor strength - Isometric hip abduction strength - Vertical jump - Hop for distance - Side hop   Only 19% passed all quantitative criteria  Qualitative measurements:   - Single leg hop and hold - CMJ with LESS   51% passed all qualitative measurements  Only 16% athletes passed both quantitative and qualitative criteria for RTP | Only 16% of pivoting athletes met all RTP criteria, but athletes who were already tested by their own physical therapist more commonly met all RTP criteria (23% versus 10%), indicating those not tested might have a higher chance for a second ACL injury. Interestingly, 77% of the athletes tested by their primary physical therapist were given RTP advice, which was inconsistent with the ACLR prac­tice guideline. Based on these results, more atten­tion needs to be paid to implementing the ACLR practice guideline and ACLR rehabilitation and RTP measurements need to be incorporated into general physical therapy education. |
| Von Aesch, A. V.  Perry, M.  Sole, G. | Qualitative study | Physiotherapists working in New Zealand | 15 physio’s from 6 private clinics | To understand physiotherapists' experiences and influencing factors in ACL injury rehabilitation decisions to determine the research needed to support physiotherapists in management of ACLR. . | ACLR - knee | General population | Some participants use guidelines based on Greg Myer and Tim Hewitts principles.  RTP time   - 6 months if passed all criteria - 9 months high impact sports   Some physios had clear guidelines for RTP:   - 92% of IKDC International Knee documentation committee subjective form - 92% of strength, neuromuscular assessment and RTP programs.   Considerations in RTP post ACLR:   - psychological issues: confidence. - Physios use patient-centred approach: patients to make fully informed treatment decisions. - Prefer a milestone approach rather than timeframe approach to progression. - Patient outcomes improved with good MDT collaboration - Conflicts in MDT include a lack of communication between health professionals especially between the surgeon and physio. They suggested that stronger relationships build trust, promoting open discussion and sharing of useful information. | No specific criteria was discussed for RTP. This study highlighted the need for a publication of translational consensus statements, on exercise prescription and return to sport and occupation criteria. |

### Rehabilitation Specialists

| **Author** | **Type of study & data collection** | **Participant’s profession** | **Sample size** | **Purpose** | **Injury location** | **RTP level – patients activity level profile** | **RTP practices, attitudes and behaviours** | **Conclusions** |
| --- | --- | --- | --- | --- | --- | --- | --- | --- |
| **Hamstring injuries** | | | | | | | | |
| Di Trani Lobacz, A.  Glutting, J.  Kaminski, T. W. | Cross sectional study - Electronic survey | Certified athletic trainers  73.6% -Master’s degree | 1356 | How AT treat and rehabilitate hamstring strain injury (HSI), and  Have an understanding of the beliefs and opinions on current management practice effectiveness. | Hamstring strain injuries | Not described  (The assumption within the study is active sportsmen and women from high school age onwards) | Importance in RTP criteria:  90% Functional testing  ~80% active ROM  ~70% manual muscle testing  ~45% palpation  ~45% passive ROM  ~40% hamstring special tests  35% lower extremity biomechanical assessment  ~20 bilateral isokinetic tests  No consensus on MRI, MSK ultrasound, and upper extremity biomechanical assessment  Only 62% of AT were confident that a re-injury will not occur. | Wide variety on HIS management. Many AT want better treatment and rehabilitation guidelines and methods due to the high incidence and re-injury rate of HIS. Despite individual risk factors, AT can use their clinical skills better is they have a set protocol that can be adapted towards individualised athlete care. AT appear to lack confidence therefore having an evidence-based battery of treatment and rehabilitation methods and RTP criteria could lead to more confident RTP decisions and reduce the rate of HIS. |
| **Knee injuries** | | | | | | | | |
| McVeigh, F.  Pack, S. M. | Qualitative study | Rehabilitation therapists   - British Association of Rehabilitators and Trainers (BASRaT) - Athletic Rehabilitation Therapists Ireland (ARTI) | 8 | To investigate if rehabilitation therapists are able to identify if an athlete is limited to RTP due to fear or re-injury as a factor and if so, when and what processes were used to address this factor. | ACL injury - knee | Not described | Two themes emphasised for rehabilitation therapists is communication and education.   - Athlete-therapist relationship is important.   Misinformation = unrealistic RTP expectations from players, coaches, managers. Education lowers RTP pressure. They educate athlete on rehabilitation and RTP protocol but information on RTP protocol is not described.  Fear of re-injury:  early stages of rehabilitation > late stages of RTP   - Mitigate this with goal setting, more time with athlete and education.   In late stage:   - associated with mechanism of injury esp. fear of contact or specific event. - Important to gradually transition back to full sport. (methods not described) - Emphasised importance of psychological readiness for RTP but no method of assessment, written in documents or specific criteria used.   Rehabilitation therapists acknowledged lack psychological training.   - No referral to sports psychologist noted | Communication and education is fundamental for the athlete-therapists relationship. This study didn’t recognise fear of re-injury as a limiting factor in RTP. Therapists felt that their training is insufficient to address psychological aspects properly in ACLR. Validated tools for assessing RTP after ACLR were lacking. |

### Multidisciplinary Team

| **Author** | **Type of study & data collection** | **Participant’s profession** | **Sample size** | **Purpose** | **Injury location** | **RTP level – patients activity level profile** | **RTP practices, attitudes and behaviours** | **Conclusions** |
| --- | --- | --- | --- | --- | --- | --- | --- | --- |
| **RTP practices** | | | | | | | | |
| Barrette, A.  Harman, K. | Qualitative interview study | Rehabilitation specialists =  1 physiotherapists  1 chiropractor  1 athletic trainer | 3 | To explore the factors associated with the experiences of sub-elite athletes who play through pain in gymnastics, rowing, and speed skating. | MSK injury | Sub-elite athletes age 10-25 individual sport (gymnastics, speed skating and rowing) | Decision-making practices affected by:  Athletes dreams/career prospects   - long term consequences often downplayed during games or scholarship opportunity - Try first aid to get athlete through the injury but if injury too severe medical team will intervene.   Pain behaviours   - Rehabilitation specialists not always able to identify pain behaviours in athletes - Important to know the athlete: pain behaviours are difficult to identify when the athlete is not familiar to the medical team - Important to recognise performance errors to remove them from the field - Non-verbal expressions versus the severity of the injury triggers questions on athlete behaviour. | This study provided insight into the relationships between athlete, coach, and rehabilitation specialist. Dreams and goals are important to athletes and can outweigh the potential consequences of playing through the pain. Rehabilitation specialists need education on athlete pain behaviour. Athletes may limit their communication regarding pain to their coaches and rehabilitation specialists. A good relationships between athlete and rehabilitation specialist is linked with more involvement into the injury. A good relationship will allow rehabilitation specialists more opportunity to guide and educate athletes make informed decisions and understand the consequences of playing through pain. |
| Beardmore, A.L.  Handcock, P.J.  Rehrer, N. J. | Questionnaire cross sectional study | Elite and club level coaches (n=19), doctors (n=11), physiotherapists (n=15), and trainers (n=13) involved  with New Zealand rugby. | 58 | To analyse the RTP readiness practices in  in New Zealand rugby union.  The study also identified personnel involved in RTP  decision-making, and described the procedures that  help in make RTP decisions such as RTP criteria and tests  used in RTP assessments in rugby union. | MSK injury | Rugby players in New Zealand   - National Provincial Champion (NPC) and club level | Procedure for determining RTP readiness:   - Fitness testing: Performed by 50% NPC sides and 27% clubs - The use of procedures to assess RTP fitness: 48 % club sides are irregular with the use whereas majority of NPC make use of fitness tests. - Full recovery of the athlete essential prior to RTP: 16% of all participants: Only 21% of NPC and 12% of club personnel   Important criteria for RTP:  80% Physiotherapist examination/ clearance  78% Doctors examination/ clearance  78% Participation in final team run  65% Full participation in team practice  64% Ability to complete fitness testing  55% When player feels right  40% Participation in a light team warm up  28% The importance of the player to the team  22% Limited participation in team practice  0% Performance during weight training exercises  RTP fitness tests should consists of (in order of importance):  1.ability to complete position specific drills  2. injury-specific tests  3. sprint tests (acc/dec)  4. walk/jog/run patterns  5. tackling drills  6. scrummaging activities  7. agility speed tests  8. jumping/hopping tests  9.lifting drills  10. down & up drill  11. push up variations  12. beep test or 3km run  Responsible in RTP process:   - Physiotherapists ranked highest for administering and assessing fitness testing procedures. - Trainers and doctors have smaller roles in fitness testing. - Not always close agreement in individual personnel roles in the RTP decisions | The use of fitness testing prior to RTT and RTC is limited. In RTP decisions, fitness testing ranked 5^th^ and opinion of the physiotherapist and doctor considered highly. The lack of assessment towards the athletes functional ability restricts RTP assessment. Personnel with training or skills in the functional demands of the sport have little input in RTP process. RTP decisions are given to personnel with good skills in clinical assessment.  The early return of injured players into competition could be a reason for the significant injury problem in New Zealand rugby union. Not all rugby teams use standardised fitness testing or standardised assessment procedures in RTP. There is concern that full recovery is not always achieved when athletes RTC after injury. |
| Boudier-Reveret, M.  Mazer, B.  Feldman, D. E.  Shrier, I. | Cross-sectional survey | Certified Canadian sport medicine  doctors (MDs) and sport rehabilitation specialists  (physiotherapists (PTs) or athletic therapists (ATs)) | 464 | To examine the criteria  Used by sport medicine clinicians  to make return to activity (RTA) decisions in children with  MSK injuries. | MSK injuries – multiple areas | Adolescent competitive athletes e.g. 9-13 | Most common tools used in RTP criteria:   - **Sport specific skill:** standardised testing (e.g. shuttle run, sport-specific drills etc.) >observed field test >subjective report of game performance - **Pain:** Impact of pain on function > VAS - **Swelling**: palpation> visual inspection > girth measurement - **Strength:** MMT > endurance > dynamometer - **ROM:** visual inspection > goniometer - **Proprioception/balance:** one leg eyes closed > balance device > one leg eyes open > sport specific balance   RTP cut off values:   - MD, PT & AT ideally want normal but would allow RTP if athlete had mild to moderate restriction for sport specific skill, pain, swelling, strength, proprioception/balance. - 12-14% of AT & MD would allow RTP if ROM has a moderate to severe restriction. - 45% of respondents willing to return a child to activity if there was more than minimal pain (‘minimal’ level of pain was not defined in this study due to lack of definition within sports medicine guidelines) - A minimum strength of 90% compared with the uninjured side is needed. Just over half (53.2–57.4%) of the respondents felt that 90% was insufficient and would prefer equal strength before returning the child to activity.   Pain appears to be considered the most important for MDs (41.0%) and ATs (18.1%).  Balance not ranked as very important.  AT and PT were most similar compared to MD vs PT or MD vs AT.  PT (16.1%) used sport specific skill most commonly. | The most important clinical sign to determine RTA was pain. Sport skill: (2^nd^ most common choice) generally requires the child to have no restrictions before RTA, however mild restriction is often allowed.  Although normal balance was considered important before RTP, it was ranked as the lowest priority of all signs. Therefore, many children are likely allowed to RTP having deficits in this area.  The results of this study suggest areas of agreement and disagreement between different sport medicine specialists (MDs, PTs and ATs) in the criteria used in RTP decisions following MSK injuries in children. |
| Chen, Y.  Buggy, C.  Kelly, S. | Qualitative study | Senior support staff:   - 5 medical staff - 6 coaching staff - 4 other management staff | 15 | This study aimed to assist coaches and management in decision making to improve rugby players health, welfare, safety and long term well-being | Health and safety in rugby | Elite rugby union | RTP decision making:  Start of the season medical team discuss players health, safety and treatment protocol if injury were to occur during the game.  RTP depends on diagnosis and prognosis of injury   - Collaborative decision making is preferred as they need to balance performance and team goals with players health and safety. - Medical team known to be more conservative in RTP approach - Conflicts between medical team and coaching team are known   Influencing factors   - Squad importance - Coach pressure - Athlete desire to play – they can hide injury - Athletes ability to play - pain threshold   Medical management:  Medical team monitor health related data, training workloads, practice on-pitch case scenarios and perform post-match injury assessments/follow ups.  This study stressed the importance of the medical team and the athlete needing to be empowered to make safety related decisions. The study noted that medical team may be unable to openly discuss safety related concerns due to the lack of support from coaching and management team.  Framework developed:  (Can be considered from two dimensions)   1. Management commitment to safety  - Safety prioritisation - Safety empowerment - Safety justice  1. Players involvement in safety  - Safety prioritisation - Trust in co-workers safety competence | This study gives insight into the rugby unions player’s safety. The theoretical framework can help inform and frame the decision making process being used by coaches, managers and medical personnel. |
| Geldenhuys, A. G.  Burgess, T.  Roche, S.  Hendricks, S. | Online survey | Sports physicians/team doctors, orthopaedic surgeons, physiotherapists, biokineticistsand/or sport scientists/strength and conditioning practitioners.  South Africa  35.9% physiotherapists  34.4% orthopaedic surgeons  21.9% biokineticists  7.8% sports physicians | 64 | The aim of this study was therefore to investigate the views, current practices and barriers encountered among health and sport practitioners to facilitate RTS following musculoskeletal injuries in | Musculoskeletal injury | Rugby players (rugby union and rugby 7s) – amateur, school, university, semi-professional and professional | % of very important RTP criteria rating:  (non-contact training : contact training : match)  Presence of pain   - 82.8%: 79.7% : 79.7%   Subjective function and/or disability rating:   - 39.1 : 73.4% : 68.8%   Player subjective feelings of readiness or psychological readiness   - 62.5% : 65.6% :73.4%   Injured players position   - 23.4% : 40.6% : 43.8%   Players years of experience   - 14.1% : 14.1% : 20.3%   History or previous injury to same region   - 56.3% : 50% : 48.4%   History of previous injury to different region   - 18.8% : 20.3% : 23.4%   Nutritional status of player   - 9.4% : 10.9% : 9.4%   Socio-economic circumstances of player   - 6.3% : 3.1%: 4.7%   Genetics of player   - 10.9% : 6.3% : 4.7%   Medications and supplementation usage of player   - 12.5% : 18.8% : 21.9%   Pathological status and/or stage of healing   - 59.4% : 62.5% : 73.4%   Duration time from surgery   - 63.6% : 72.7% : 70.9%   Level of cardiovascular fitness   - 25% :39.1% : 54.7%   Workload   - 34.4% :45.3% : 59.4%   Ability to balance   - 31.3% : 40.6% : 42.2%   Proprioceptive ability   - 39.1% : 53.1% : 50%   Biomechanical alignment   - 18.8% : 35.9% : 35.9%   Muscle strength/power   - 56.4% : 69.1% : 76.4%   Muscle flexibility   - 26.6% : 40.6% : 50%   Muscle endurance   - 35.9% : 56.3% : 64.1%   ROM   - 54.7% : 67.2% : 70.3%   Speed/sprinting performance   - 29.7% : 43.8% : 64.1%   Level of agility   - 31.3% : 48.4% : 65.6%   Ability to complete sport specific drills   - 64.1% : 73.4% : 85.9%   Influencing factors/barriers in decision making:  Timing in relation to competition/tournament: often  Timing in relation to season: often Media perception: never Pressure from coach or management: sometimes  Fear of litigation: rarely  Influencing factors/barriers RTP protocols:  Protocols are too time-consuming to perform: sometimes  Existing protocols are not appropriate for rugby settings: sometimes  Unable to access relevant publications regarding RTP protocols: sometimes  Existing protocols are not appropriate for MSK injuries: rarely  Equipment or facilities are not available to implement protocols: sometimes Health and/sport professionals are not trained to implement protocols: sometimes  Protocols have not been validated: sometimes  Restrictions related to public health emergency or humanitarian crisis: rarely  Socio-economic circumstances: rarely  Influencing factors/barriers to use of assessments:  50% insufficient financial means to cover tests  43.8% insufficient time to perform tests  43.6% insufficient equipment or facilities  37.6% insufficient availability of health and sport practitioners | Health and sport practitioners value the importance of RTS protocols, however, there is a need to develop and publish frameworks to better guide return to rugby following musculoskeletal injuries. A comprehensive range of RTS criteria related to time frames, clinical, functional, psychological and sport-specific factors were considered to be both important and practical to evaluate among practitioners. Common barriers encountered during return to rugby involved time-constraints and lack of access (to funding, equipment and/or health and sport practitioners). Future research should therefore focus on developing return to rugby protocols with consideration of the comprehensive range of criteria, along with awareness of the common barriers encountered during the process. The return to contact phase may be the most important component as it sets rugby apart from most other sports. |
| Hess, C. W.  Meyer, B. B. | Qualitative study | Performance management team (PMT)   - Coach - Physiotherapist - Sport psychology consultant - Case manager - Athlete | 5 | This study aimed to explore the experience of a single performance management team through two injury and rehabilitation cases leading up to and into the 2014 Olympic Winter Games. | Case 1   - Meniscus tear   Case 2   - ACL | 28 year old female athlete slopestyle skier operating outside the National Sport Organisation for the previous 10 years | One athlete experiences in two injury cases. From Case 1, the lack of collaboration, conflict and negative experience between the multidisciplinary team (PMT) was due:   - being unfamiliar with each other - had different professional sport culture and norms - geographically in different locations - pressure to win in the Olympic Games.   Whereas in case 2, they had been working together for so long (in case 1) that they were now   - familiar with each other - Their differences in professional styles because a complement in their collaborative efforts - They were now all located at the Olympic Games so could see each other daily - No pressure to win but rather just enjoy the experience of being at the competition   RTP decision:   - Case 1 was able to go through full rehabilitation and time - Case 2, the PMT needed to speed up rehabilitation and time for RTP   On case 2 RTP was made using a panel of medical professionals. The coach had made a video showing the athlete performing functional sport specific routine as the PMT were concerned that the athlete would fail RTP if she was forced to go traditional RTP tests. | Even though there are advances in medicine and technology, RTP outcomes in sport are still suboptimal. This study showed that a way to improve outcomes is by improving the integration and functioning of the team surrounding the athlete or sport team. Understanding team function, the sociocultural context that surrounds PMTs, and the challenges faced by each member can help to improve rehabilitation outcomes, RTP and the experiences for athletes and team members. |
| Horan, D.  Kelly, S.  Hägglund, M.  Blake, C.  Roe, M.  Delahunt, E. | Qualitative semi-structured interviews | Players (n=17), head coaches (n=7) and medical personnel (n=8) | 32 | The aim of our study was to use qualitative research methods to explore the knowledge, attitudes, and behaviours of players, head coaches, and medical personnel in the Irish Women’s National League (WNL) to injury prevention and injury management. | MSK injury | Elite level women’s football in Ireland | Themes   - Injuries   - Common injury   - Player availability   - Rick factors - Prevention of injury   - Monitoring   - Injury surveillance   - Injury prevention strategies   Barriers to comprehensive RTP decisions   - Student medical personnel increase players risk of injury - Lack of medical staff available at weekly training sessions - Lack of same day assessments - Sharing medical personnel at matches causes frustration and lack of confident in decision making   Injury management   - Medical personnel need to have knowledge on RTP strategies - Interpersonal and communication skills are key attributes - Clear communication between coach and medical - Decisions need to be in best interest of players (although two respondents believed this was not always the case) - Concussion management is important - Injury prevention knowledge is important - Coaches tend weigh up information from medical and players plus use their own judgement on injury risk and game importance when considering management of injured players - Players honesty in injury communication should not negatively affect availability for match selection - Player importance to team performance and success can influence pressure from coaches on to medical team   More experienced players are perceived to be trusted more with their own decisions regarding injury and availability whereas less experienced often have medical personnel and coach making the decision | Poor quality and availability of medical care and S & C support were considered to be a major obsta- cle in the effective implementation of injury risk reduction strategies and successful return-to-sport practices. More original research is required in elite-level women’s football to explore injury risk factors, injury prevention initiatives, and contextual return-to-sport strategies, so that players, head coaches, and medical personnel can use evidence that is both up-to-date and specific to their environment. |
| Mazer, B.  Shrier, I.  Ehrmann Feldman, D.  Swaine, B.  Majnemer, A.  Kennedy, E.  Chilingaryan, G. | Cross sectional study | Canadian Medical doctors (MD), physical therapists (PT), and athletic therapists (AT)   - sport medicine specialty organizations. - All had post graduate interests in sports | 464 | Compare the management approach of MSK injuries in active children between sport medicine and rehabilitation professionals | Various injury scenarios discussed | Adolescent and childhood competitive athletes  -Active children between ages 7-16 years of age. | Clinical, personal, and sport-related factors affected RTP decision in the case scenarios in medical doctor, physiotherapist and athletic trainer. Dependant on age, severity of injury:  Order of most common answer. Answers reported as a collective but varying percentages in study  Case 1 No effusion, laxity remains, and reports mild pain after jogging 10 minutes.   - Majority: return with restriction   Case 2 Bone scan is normal. No pain at rest & during most gymnastic activities but is very sore after practicing back walkovers (lumbar hyperextension). Neurological signs all  normal.   - Majority: return with restriction   Case 3 On-court examination. She walks without a limp, it is painful to run, but she feels she can finish the tournament.  +- 50%: return with cautionary advice  1/3: do not return  +- 20%: return with restriction  Cade 4 He has a mild limp if he is very active, usually only 1 to 2 times per week. Sx increased last week when he increased his snowboarding. Mother kept him out of activities.  Similar amount recommend return with cautionary advice or return with restrictions  Case 5 There is mild pain at the end of extension and flexion. There is no concern of scaphoid or other fracture. Pain when he dives and if he ‘‘misses’’ the dive. He wants to continue the competition.  Majority: return with cautionary advice  +-20% return with restrictions  Factors considered:   - Pushy parent - Cautious parent - Protective equipment - Past injury - ↑MSK maturity - ↑game importance - Longer time since injury - Team sport - Less risky position | Variability in opinions among specialists (both within and between professions) in the management of musculoskeletal injuries in children and youth. There is a need for evidence-based practice guidelines to ensure active children are managed and RTP safely after injury. |
| Müller, P. O.  Helbling, M.  Verhagen, E.  Spörri, J.  Bolling, C. | Qualitative semi-structured interviews | - (n=2) Active or retired athletes with at least one severe injury - (n=2) coaches - (n=2) S&C coaches - (n=1) psychologist - (n=3) physiotherapists   (n=2) physicians working in | 14   - (2 athlete - 2 coach - 2 S&C - 1 psych - 3 PT   2 physician) | The study explored perspectives and expe- riences regarding the RTS process in high-performance Snowsports to understand current practices, challenges and opportunities. | Severe MSK injury | High performance Snowsports (alpine skiing, freestyle skiing & snowboarding) | RTS is a process   - Requires goals (short and long term through different phases)   - Small steps help reach big goals   - Shaped by expertise: physician (swelling, inflection), PT (ROM, strength, mass)   - Goal: RTP as quickly and safely as possible   - RTP process = milestones and criteria     - Milestones = points in timeline that structure RTP process     - Criteria = verify that intermediate goals are achieved, assess deficits etc     - Milestones and criteria-based approach easier in early and middle rehab but lack in later RTS stages   RTS for individual athlete   - Athlete-centered approach - Trust in the experts and the process - Build a safe environment for athlete   Multiple resources and many experts   - RTS is better if you are able to use multiple resources (expert networks and infrastructure)   Expert cooperation, communication, shared planning and work integration = RTS success | In high-performance Snowsports, current RTS best practices involve an athlete-centred process with distinct phases and milestones monitored by functional and clinical criteria. In this context, the RTS should be considered a continuous process supported by the experts and resources tailored to the athlete’s needs. In addition, there is a need for more interprofessional collaboration and better information flow between the different RTS phases. |
| Read, D.  Rosenbloom, C. | Semi-structured interviews | Doctors (n=8), physiotherapists (n=6), head of medicine and performance (n=8) | 20 | It is important to understand how, when, and why analgesic treatments to mask pain modify tolerance for risk of (re)injury. Identifying current practices can inform guidance, policy, and medical governance to help practitioners and improve player welfare, cognisant of real-world work settings. | Musculoskeletal injury | Professional men’s football in the United Kingdom | Based StARRT framework:  Contextual factors in using analgesia to mask injury functions as risk tolerance modifiers (willingness to use analgesia to facilitate return to play):   - access to medical resources   - Physician employment   - Staff and facilities   - Medical networks   “The time, resources, and information available to medical staff impacted the ability to assess players and their risk tolerance in relation to analgesia.”   - trust between staff and players   - manager support   - player health benefits   - external practitioners   “decision-making is a multistakeholder process and the nature of relationships with players and staff influenced risk tolerance”   - prioritising short-term success   - availability   - player vulnerability   - private medication use   “decisions are made against a performance working environment that privileges short term success over long term wellbeing” | The data shows that the decision to use pain management in professional men’s football is influenced by the players and the staffs working context. Furthermore, practitioners viewed potential harm to a player from a socio-economic and physical perspective. There finding support the adoption of a patient centered care approach and can inform clear recommendations from international and national governing bodies. |
| Read, P. J.  Jimenez, P.  Oliver, J. L.  Lloyd, R. S. | Cross sectional, quantitative online survey | Representatives of sports science and medical teams  48.8% S&C  24.4% sport scientists  12.2% Physiotherapists | 41 | To examine the current practices and perceptions of practitioners that implement injury reduction strategies at elite male academy soccer clubs. | Non-specific MSK injury | All age groups of academy soccer players – United Kingdom | 80.5% responders have specific RTP criteria for relevant injuries.  Most common methods used during RTP:   - 95.1% monitored training load - 42% rating of perceived exertion (RPE) - 27.2% global positioning systems (GPS) - 25.9% heart rate   48.8% use a neuromuscular assessment including:   - countermovement jump - drop jumps (with measures of jump height and reactive strength index) - wellness questionnaires - six seconds cycle ergometer sprints - groin squeeze tests.   Highest risk of injury:  49% 13-16 year olds  24% all age groups  Reported most common cause of injury:  29% contact  28% overuse  25% non-contact  18% Other responses e.g. growth related, faulty mvt, fatigue, previous injury  Perceived importance of physical related components to prevent injuries:   - lower limb strength (74%) - proprioception (51%) - movement skill (46%) - hamstring strength (44%) - mobility (38%)   Common methods to prevent injury: resistance training (73%), core stability (29%), agility training (22%), balance training (20%), flexibility training (20%), plyometrics training (17%) | Preventing injury is emphasised with strength, proprioception, movement skill, and mobility being essential for injury prevention. However, these components are not used in assessment during RTP decision-making. This study highlighted the disconnect between preventing injury versus re-injury. |
| Riendeau, C.  Parent-Houle, V.  Lebel-Gabriel, M. E.  Gauvin, P.  Liu le, Y.  Pearson, I.  Hunt, M. R. | Qualitative study – in-depth semi-structured interviews | Athletic trainers and Physical Therapists  -Canadian population | 11 | Understand the ethical issues experienced by university sports team athletic therapists and physical therapists | Ethics in RTP decisions | University varsity teams working with team, individual athletes from non-contact through to contact sport | Ethical issues confronted:   - RTP - confidentiality - professional boundaries   RTP considerations   - Athletes must be educated potential consequences in contentious RTP decisions - Ensure clear communication about the risk of quicker RTP especially when there is pressure from coaches and athletes. - RTP process and decisions must be controlled and objective - ATs and PTs able to collaborate with management but can be difficult when there is conflict of opinion e.g. coach disputes a clinical decision. - The amount of pressure varies between settings, teams and players - Good working relationship between coaches and therapists = less disagreements. Effective communication include respect and trust - MDT collaboration consideration in decision-making authority. Role definition is important as there may be hierarchy in some decisions - Physician often makes final RTP decisions in complex cases often with input of therapists and others. - If no physician present, therapists are then responsible for decisions but clarity in how decisions are made in these situations is less clear. - ATs & PTs expressed that an algorithm would be helpful for on-field RTP decisions esp. identifying who is responsible for decisions. Establishing this rule would assist in resolving conflicts that arise when there are differences in resources between universities (including availability of skilled health professionals)   How AT & PT seek answers to ethical issues:   - Colleagues working in the same location are important sources of support - Clear guidelines reduce ethical issues - RTP concussion guidelines are helpful, support communication and give a clear foundation during the decision-making process - Only 1 participant had access to clear RTP guidelines for MSK injury. The participant felt that this tool was helpful when the therapist was pressured to return an athlete to play too early - AT & PT draw on a variety of sources and experiences when trying to understand and manage ethical dilemmas.   AT & PT long term effects of difficult ethical decisions:   - AT & PT : athlete-centred decision - Being responsible for decisions is difficult and can result in repercussion or fallout in some situations. - Although therapists are perceived to make fair decisions, there is concern that trust is easily lost when other team members feel that a decision made was unjustified - Team sports are more complex as decisions due to decisions affecting the team - Concern that other team members do not understand or appreciate the professional standards that therapists are expected to uphold. - Therapists often feel anger, helplessness, sadness, empathy, frustrated and isolated within ethically challenging situations including RTP decisions - Therapists feel anxious or stressed about the repercussions of ethical decisions | Ethical issues reported by therapists are similar to the sports medicine physicians. Ethical issues are experienced differently due to differences in professional roles and identities.  The study highlighted how sports therapists experience and manage ethical issues. Frustration and anxiety can surround ethical issues. Sports therapists will benefit from better communication and collaboration, awareness of university policies and professional codes of ethics, opportunities to consult with colleagues and mentors and access to guidelines for specific clinical decision-making process surrounding ethical dilemmas |
| Shultz, R. Bido, J.  Shrier, I.  Meeuwisse, W. H.  Garza, D.  Matheson, G. O. | Survey questionnaire cross sectional study | Sport medicine clinicians:   - Physicians - Physiotherapists - Chiropractors | 67   - 48 male - 38 female | To describe the variability in the RTP  decisions and opinions of experienced team clinicians  relevant to 19 factors described in a RTP decision-making model. | Generalised injury | Athletes ranging from soccer, running, rugby, soft ball etc.  Team clinicians for sports:  64.2% soccer  55.2 running  43.3 track and field  37.3 Skiing  35.8 Basketball  34.3 Cycling  34.3 Tennis | Definition of “cleared for participation”:  (participant % answering yes)   - 95% The athlete is asymptomatic with no elevated risk of re-injury and no risk of long-term sequelae - 85% The athlete does have symptoms or signs, but they are not of sufficient concern to place the athlete at risk for either acute re-injury or long-term sequelae - 23% The athlete has an increased risk of acute re-injury but no increased risk of long-term sequelae - 29% The athlete has no increased risk of acute re-injury but does have an increased risk of long-term sequelae - 6% The athlete has an increased risk of both acute re-injury and long-term sequelae   Factors considered important according to decision-based model 0=least NB → 8=most NB  (Ranking 5-8/8):  Step1 medical factors  Potential seriousness 72%  Symptoms 74%  Signs 76%  Functional testing 62%  Personal medical testing 45%  Laboratory tests 31%  Psychological state 19%  Patient demographics 16%  Step2 sport risk modifiers  Type of sport 57%  Competition level 21%  Ability to protect 10%  Position played 0%  Limb dominance 12%  Step3 decision modifiers  Timing of season 65%  Pressure from athlete 55%  External pressure 12%  Masking the injury 27%  Fear of litigation 12%  Conflict of interest 7% | Injury affects a range of people (athletes, team, coaches, and parents) and make RTP decisions complicated.  This study felt that although more data is needed to understand clinical decision-making, a more detailed definition of clearance would assist quantitative analyses. Most sports medicine clinicians in this study believe that injury risk factors are important to consider. |
| Shrier, I.  Safai, P.  Charland, L. | Online survey cross sectional study | Canadian Stakeholder groups:  Clinician:   - Medical doctors (sports physicians) - Physiotherapists - Athletic trainers - Chiropractors - Sports massage therapists - Vs - Non clinicians: - Athletes - Coaches - Sports associations - Olympic Committee | 736 Clinicians  +  175 stakeholder group  (911) | Comparison of professionals’ opinion on RTP criteria and who is should decide RTP. | Generalised injury | Canadian sport population | The relative importance (from Mandatory to Not a Criterion) of potential injury risk-related criteria to consider in RTP decisions:  Clinician injury risk-related criteria in RTP decisions: (rated as mandatory)   - ±78% state of healing - ±82% risk of re-injury - ±66% risk of short-term problems - ±65% long term problems - ±31% risk of injury to team members - ±25% risk of injury to opponents   Clinician non-injury risk-related criteria in RTP decisions: (rated as mandatory)   - ± 20% Desire to compete - < 45% ↓Injury risk: equipment - ± 45% ↓ injury risk: modified training - ± 25% psychological impact: competition - < 5% potential financial loss - <5% potential loss of competitive standing   Top decision-makers to assess RTP considering healing, risk of re-injury, short term and long-term consequences.   - Medical doctors 33,3% - Athletic trainers 33.3% - Physiotherapists 23,3% - Chiro 10% - Coach 6.66%   Who is best to assess:  Healing state   - 63% medical doctors - 50% ranked PT   Re-injury risk   - 78% MD - 76% PT - 56.5% AT - 56% Chiro | Medical doctors, physiotherapists and athletic therapists are best able to assess factors related to risk of injury and complications from injury. Criteria used to by each professional to assess state of healing, risk of re-injury, risk of short- and long- term problems were not stated in this study.  RTP decisions were different within stakeholder groups compared to between-stakeholder groups. Each clinician group rated their own profession above other clinician groups when assessing certain RTP criteria. Advantages and disadvantages of non-injury factors that affect the well-being of the athlete were best assessed by the athlete, coach and sport associations. Open conversations and collaboration is important. |
| Shrier, I.  Serner, A.  Wangensteen, A.  Steele, R. J.  Weir, A. | Feasibility study with a descriptive prospective case-series. | Athlete, physiotherapist and sport physician | 10  5 physiotherapists  5 physicians | To determine if re-injury risk assessments using Bayesian methods and risk tolerance using questionnaires between clinicians and athletes in real-world RTP settings is feasible | Athletes with ongoing hamstring and groin injuries (partaking in other studies) | 10 injured athletes (9 football, 1 basketball) was 26.7 years (range: 22–37). | This study conducted within on-going groin and hamstring studies - “Triplet” (athlete, sport physician and physiotherapist) caring for an athlete recruited.  Bayesian methods applied to estimate risk of re-injury (risk tolerance) over a 2 month period based on available clinical knowledge and outlined activity level.  Risk assessments:  The risk of injury to same location/type of injury:   - Reasonably similar in 3 cases (athlete 1,6,&9) - Slight divergence of opinion in 3 cases (athlete 4, 5, 10)(physiotherapist and athlete differ) - Significant divergences for 2 cases (athlete 2 & 7)   ***3 athletes (athlete 2, 4 and 5) had subsequent injury with highest probability of injury risk given by physician, physiotherapist and athlete in each case respectively  Risk of subsequent injury:   - Most opinions similar except for 1 case (athlete 7) with distribution of re-injury risk being 40-50% from 5-15%   Risk tolerance modifiers   - Timing and season - Pressure from athlete - External factors   Factors NOT identified as affecting risk tolerance   - Clinician conflict of interest - fear of litigation - fear of blame   Study reported that risk tolerance modifiers influenced 13 of 27 RTP decisions | This study showed that it is feasible to study risk assessment among clinicians and athletes in real world context. The physiotherapist, physician and athlete may disagree on the level of risk. Disagreements between clinicians in RTP decisions highlight the need for a more transparent system to report differences in risk assessment (risk estimation and risk tolerance) to enhance understanding when opinions vary. |
| Yeomans, C.  Comyns, T. M.  Cahalan, R.  Warrington, G. D.  Harrison, A. J.  Hayes, K.  Lyons, M.  Campbell, M. J.  Kenny, I. C. | Cross sectional study – survey design | Medical professionals and rugby coaches of the top 58 amateur rugby clubs in Ireland. | 44 (unable to distinguish the medical professional from sport coaches) | To ascertain current injury surveillance and player education practices in Irish amateur rugby  union. | General musculoskeletal injury and concussion | Irish amateur rugby players | Injury documentation conducted by:   - 75% team medical staff, incl. physiotherapists and doctors - 13% coach - 8% administrative staff incl. club development officers, secretary and welfare officers   Training load monitoring:   - Used by 36% of clubs: 13% RPE, 6% GPS tracking, 6% electronic application (TrainheroicTM) - Conducted by: 38% strength and conditioning coach; 12% rugby coaches; 50% not specified   Physiotherapists   - 96% present at matches - 61% present at training sessions   Doctors   - 75% present at matches - 5% present at training sessions   Coaches AED certification:   - 55% have AED certification - 11% did not have AED certification - 34% unsure of certification   100% of clubs operate a RTP protocol   - 64% all injuries including concussion - 36% concussion protocol only   95% of clubs operate a RTT protocol, 5% unsure   - 61% for all injuries including concussion - 34% for concussion injuries only   Injury assessment within 48 hours of injury   - 93% conducted by physiotherapist   The SAFE rugby training programme:   - delivered to 79% of these clubs (members ranged from 1-40 per club with SAFE training) - 16% unsure if anyone in the club was SAFE trained - 5% no-one had SAFE training at their club   *The SAFE Rugby training program [implemented by Irish Rugby Football Union (IRFU)], provides rugby-specific, pitch side emergency care training and skills to both medical and non-medical individuals involved at all levels in Irish rugby.  Player education on injury prevention conducted by 71% of clubs and recognition and recovery of concussion in 82% of clubs  Player education conducted by   - 42% medical staff - 29% head rugby coach - 3% S&C coach - 3% club development officer - 23% unsure | Of the 91% of rugby clubs use different methods and people to monitor injury. Usually the medical professional is responsible for injury monitoring. A comprehensive centralised system would be implanted to effectively monitor injury trends. Training load was only monitored by 36% of clubs with 38% of these clubs using a S&C coach.  Monitoring injuries, monitoring training loads and improving player education can decrease injury risk. There is a need to implement educate and design injury prevention strategies to reduce injuries. |
| **Shoulder injuries** | | | | | | | | |
| Maher, N.  Willmore, E.  Bateman, M.  Blacknall, J.  Chester, R.  Horsley, I.  Gibson, J.  O' Sullivan J  Jaggi, A. | Online survey | Surgeons (n=28) and Physiotherapists (n=110) working in the United Kingdome | 138 | The aim of this study was to establish current UK rehabilitation practice following primary arthroscopic stabilisation surgery for recurrent traumatic shoulder instability. | Soft tissue stabilisation surgery for traumatic shoulder instability | Not described | Recommendations for return to non-contact sport   - 18.1% Based on length of time since operation   - 4% from 4 weeks   - 4% from 5 weeks   - 24% from 8 weeks   - 4% from 9 weeks   - 8% from 10 weeks   - 56% from 12 weeks - 37% when functional markers for RTP have been met - 10.9% based on RTP criteria   - 40% psychological readiness   - 24% presence of kinesiophobia   - 12% kerlan-jobe ortho clinical score (KJOC)   - Other: force plate testing, Oxford instability score, shoulder instability return to sport after injury (SIRSI) scale, sport-specific pathways - 31.9% don’t use specific criteria but am led by patients level of function/confidence - 2.2% other   Recommendations for return to contact sport   - 22.5% Based on length of time since operation   - 3.8% from 6 weeks   - 46.2% from 12 weeks   - 50% from 16 weeks   - 5.1% from 6 months - 21.0% based on RTP criteria   - 33.3% psychological readiness   - 25.5% presence of kinesiophobia   - 17.6% KJOC score   - 23.5 combination of objective markers and readiness questionnaires, sport-specific RTP testing and pre-set patient specific goals being achieved. - 42.8 % don’t use specific criteria but led by patients level of function/confidence as long as they have passed minimum time threshold - 10.9 % don’t use specific criteria but led by patients level of function/confidence regardless of the length of time since the operation - 2.9% other   Clinical discretion   - 20.6% surgeon - 49.1% physiotherapist with surgeons support - 15.8% physiotherapist at a certain level/grade - 12.7% any physiotherapist has autonomy to deviate from the protocol according to judgement - 0.6% N/A we do not routinely use protocols - 1.2% other   Influencing factors for timelines  15.5% functional/physical milestone  13.3% patient level of confidence  13.1% quality of reported surgical fixation  10.6% presence of hyperlaxity  8.5% age  7.4% presence of kinesiophobia  7.2% overall general health  4% presence of type I or II diabetes  3% smoking  2.8% excessive alcohol intake  Outcome measures:  Oxford instability score, ROM, VAS, patient reported improvement, disability of the arm, shoulder, numerical pain rating scale, Western Ontario Instability, singe assessment numeric, American Shoulder and Elbow, Contant score, force desks, handheld dynamometer, patient-specific functional scale (PSFS) ratings. | The findings of this survey suggest that there is wide variation in rehabilitation practice following arthroscopic stabilisation surgery. Decisions appear to be based on patient function, confidence, kinesiophobia and individual patient factors, yet time-based markers and/or minimum time thresholds dominate most points of progression. Whilst progress is often led by the patient’s level of function/confidence, there is low-level use of specific measurement tools or outcome measures to determine this reliably and objectively. Coupled with an absence of robust evidence around the biomechanical rationale for imposed restrictions, the question arises as to whether protective immobilisation and temporal limitations are necessary or whether they are at best needless and at worst, potentially detrimental.  There was a clear lack of agreement in relation to many of the questions asked during this survey. In the absence of clinical trial data, the AHPCGG will continue to work on developing best-practice rehabilitation guidelines for patients undergoing arthroscopic shoulder stabilisation surgery. The results of this survey will form the basis of a Delphi study involving key stakeholders including surgeons, physiotherapists and patients, which is due to take place across 2022/2023. This will provide, where possible, expert clinical consensus on what optimum rehabilitation should look like, including criteria for progression and use of outcome measures. |
| **Hip injuries** | | | | | | | | |
| Worner, T.  Thorborg, K.  Moksnes, H.  Eek, F. | Cross sectional survey | Orthopaedic surgeons & physiotherapists | 90 | To describe and compare current rehabilitation  strategies and views among surgeons and physiotherapists in Scandinavia treating hip arthroscopy. | Hip Arthroscopy (HA) | Active population, not described | RTP clearance:  Physio 74.2%  Ortho 50%  > 75% recommend either criteria-based or  combined criteria- and time-based rehabilitation progression.  Timeline to reach RTS milestones:  Physio vs ortho  *Recreational sport*  71% physio says 18 weeks vs 86% ortho say 16weeks  *Competitive sport*  66% physio says 25 weeks vs 86% ortho say 21weeks  Criteria used in the *rehabilitation* process:  Physio vs ortho  Objective   - ROM 95.1 vs 92.9% - PBM 86.7% vs 52% - Strength 84.5 vs 74.1 - Subjective - HAGOS 32% vs 75% - iHOT 21.7 vs 59.1% - VAS 57.7% vs 54.5% - NRS 45.7% vs 31.3% - HOS 24.4% vs 50%   Extremely and very Influential factors on RTP decisions:   - 76.3% Performance based measures (84.8% physio, 46.2% surgeon) - 86.5% Pain - 77.6% Psych readiness - 79.3% Strength (88.9% physio, 46.3% surgeon) - 38.6% ROM   Influential factors on RTP decision:   - Patient influence - Physio influence - Surgeons influence | Physiotherapists and surgeons had similar views for rehabilitation. Physiotherapy is considered important following HA by both professions. A either criteria-based or combined criteria- and time-based rehabilitation progression is used. Surgeons expected less time on crutches and quicker RTP than physiotherapists. Surgeons also used evidence-based self-reported outcomes more than physiotherapists. |
| **Hamstring injuries** | | | | | | | | |
| Balcı, Aydın  Ülkar, Bülent | Cross sectional survey study | Sports medicine specialists (n=49)  Physiotherapists (n=26)  49 working in health institutions  12 working in sports clubs  14 working in both health institutions and sports clubs | 75 | Investigated the  utilization of RTP criteria following hamstring  injuries and to suggest the causes of discrepancies  in sports medicine practice. | Hamstring injury | Not described | RTP criteria (5 point Likert scale)  ***Significant differences seen between referred importance and practical application  Referred importance VS practical application:  - Absence of pain  4.75 vs 4.83  - Psychological readiness ***  4.59 vs 4.36  - Sport specific functional evaluation ***  4.57 vs 3.55  - Flexibility ***  4.44 vs 4.61  - Nordic eccentric muscle strength ***  4.25 vs 2.87  - Isokinetic muscle strength ***  4.13 vs 2.37  - Single leg balance ***  3.93 vs 3.44  - Aerobic-anaerobic capacity ***  3.84 vs 2.83  - Imaging MRI-USG  3.00 vs 2.73  - Neuromuscular evaluation using EMG ***  2.81 vs 1.39  Factors affecting RTP decisions:   - Competition schedule- achievement goal 3.65 - Pressure from coach 3.19 - Pressure from athlete to return early 3.08 - Lack of technical equipment 2.99 - Fear of litigation 2.09 | This study found that factors that are considered important are not necessarily practiced in RTP criteria following hamstring injuries. The high rates of hamstring re-injuries may be due to the inconsistency between well-established RTP criteria and the ability to apply the criteria in practice. The non-medical constraints that sports physicians face in daily practice, mainly coming from coaches and athletes can make it difficult to implement. Understanding influencing factors in RTP decisions following hamstring injuries, especially based on sports specific models is needed. Reducing the barriers that lead to failure of following the RTP criteria in hamstring injuries will lower reinjuries, and decrease overall injury rates. |
| Dunlop, G.  Ardern, C.L.  Andersen, T.E.  Lewin, C.  Dupont, G.  Ashworth, B.  O’Driscoll, G.  Rolls, A.  Brown, S.  McCall, A. | Cross sectional study | Science/ sports  medicine RTP team made up of:   - club doctor (61 teams) - physiotherapist (33 teams) - strength & conditioning coach (26 teams) - sports scientist (9 teams) - manual therapist (2 teams)   Medical staff = club doctor & physio  Science staff = S&C coach, sports scientists, sports psychologist  Coaches & management = manager & coach (technical staff)  Player = athlete | 131 | Determine if  premier-league football teams follow a RTP process and criteria and to understand how RTP decisions are made. | Hamstring muscle injury | Professional football players from premiere leagues worldwide | RTP:   - 95% reported following a RTP continuum model for hamstring injuries.   RTPerf   - 21% teams did not report doing a RTPerf phase   Decision-making   - 80% of teams used a SDM approach involving at least 2 people. - 6% isolated decision-making - 14% combination of shared and isolated decision-making   Most important criteria by reported by teams during RTP continuum:   - RTR: 96% Clinical; 96% Functional; 54% psychological - RTT: 98% functional; 90% clinical; 69% psychological - RTP: 92% functional; 73% clinical; 76% psychological; 5% no criteria used - RTPerf: 76% functional; 45% clinical; 44% psychological; 21% no criteria used   Top criteria used during each phase:   - RTR: Absence of pain ; Hamstring strength - RTT: Training load monitoring; Hamstring strength - RTP: Training load monitoring; Functional performance/ assessment - RTPerf: Training load monitoring; Staff subjective appraisal   Frequency range with which all criteria is 90-100% met (No. of teams /131)  RTR 77% (n=101)  RTT 71% (n=94)  RTP 59% (n=77)  Contribution of decision-makers in RTP:  RTP  Medical staff 87-89%  Science staff 55-89%  Player 53-69%  Coaches &management 76-86%  Out of the challenges felt by RTP practitioners:   - 27% Team hierarchy (e.g. pressure from management) most likely to influence practitioner decision-making - 24% Match related challenges such as importance of upcoming fixture/phase of season - 21% Player related challenges e.g. compliance and pressure to progress/return. - 14% team related e.g. existing squad depth / other injuries - 10% rehab program related e.g. time restraints, isolated decision-making - 5% other challenges e.g. language barrier, limited resources | Majority of teams assess using a SDM approach and use functional, clinical and psychological criteria throughout the RTP continuum - in-line with general RTP research recommendations. However, specific criteria, metrics and thresholds used, and the specific involvement, dynamics, and interactions of staff during the decision-making process lack clarity. |
| **Knee injuries** | | | | | | | | |
| Ebert, J. R.  Webster, K.E.  Edwards, P. K.  Joss, B. K.  D’Alessandro, P.  Janes, G.  Annear, P. | Cross sectional Survey-based study | Australian Physiotherapists  Accredited Exercise Physiologists (AEP) | 223 | To investigate the pre-and post-operative rehabilitation and RTP clearance criteria and methods practiced by Australian therapists. | ACLR - knee | RTP for high demand sports e.g. AFL, rugby, soccer, netball, hockey. | RTP Time from surgery:   - 53.8% 9-12months - 22.9% 12-18 months - 22.0% 6-9 months   RTP for high demand sport:   - 98.7% Functional capacity (e.g. jump and/or hop tests) - 96% Lower limb and trunk mechanics during jumping/landing tasks - 87.9% psychological readiness - 87% knee strength - 78.5 time from surgery - 69.5% ROM/laxity - 54.7% limb asymmetry - 53.8% patient age - 45.7% PRO questionnaires - 20.2% Other e.g. sport specific skill, agility/COD, hip strength, proprioception, effusion   Tests for lower limb functional capacity:   - 84.3% A hop test battery (incl. >2 6m timed; single, tripe hop and triple cross over hops for distance) - 62.8% Star excursion and/Y-balance test - 43% single limb vertical hop - 21.5% Other e.g. T-test, figure 8, evaluation running dynamics, sprinting with acc/dec tasks, lateral and/or medial hop tests   Knee strength tests:   - 48.9%estimate knee strength from other measures such as hop capacity - 33% manual muscle testing methods - 26.7% hand held dynamometry - 11.8% isokinetic dynamometer - 10.9% refer to another professional for testing | Difference in the timing of RTP discharge and the tools used to assess patients prior to RTP. Most therapists appeared to use a test battery consisting of time from surgery, functional capacity, strength, lower limb and trunk mechanics during landing tasks and a subjective assessment of psychological readiness. |
| Lambert, C.  Rotzmann, R.  Ellermann, A.  Carvalho, M.  Akoto, R.  Wafaisade, A.  Lambert, M. | Cross sectional survey | Judo medical team members – doctors & physiotherapists  232 world class junior athletes | 50 (21 doctors & 29 Physiotherapists) | To quantify how the return to competition after an ACL rupture in Judo is perceived by athletes as compared to doctors and physiotherapists. | ACL injuries / ACL recon - knee | World Judo championshios and junior world judo championships athletes (semi-pro  All athletes under 21 years old | Perceptions:  Athletes RTC on average 5.8 months after injury or reconstruction whereas the medical team assumed the athletes RTC after average 8.9 months post injury/reconstruction.  65% of judoka achieved pre-injury performance after rehab/post injury.  Treatment methods: 55% of athletes treated conservatively had reduced performance compared to 17% that was treated with surgery  RTC tests   - Only 13% of athletes had to perform functional tests e.g. hop tests - 44% of medical team believed that they used functional tests e.g. hop tests for RTC testing - 0% mental tests were conducted in their RTC process - 26% believed to have used mental tests in RTC   Decreased level of competition after  RTC after an ACL injury:   - athletes are going back earlier to competition than doctors and physiotherapists recommend. - too early RTC post ACL = in higher risk of re-injury - low number of RTC tests used to define the right moment for RTP | Different expectation between what medical team thought the athlete was doing verse what the athlete was actually doing. No standardised RTC process for treatment, use of RTC tests and time out of competition in world class judoka. Most athletes do not complete the RTC process after injury. The use of RTC tests was uncommon, which increases risk of injury on return to judoka after ACL injury. Communication between the athlete and the medical team during the rehabilitation period can help with compliance and education on risk factors. |
| Lyng. K. D.  Rathleff, M. S.  Dean, B. J. F.  Kluzek, S.  Holden, S. | Cross-sectional mixed-method study  Questionnaire and semi-structured interviews | Healthcare professionals (HCP) – physiotherapists, rheumatologists, sports medicine doctors, orthopaedic surgeons | 255 | To investigate how international healthcare professionals diagnose and  manage Osgood-schlatters disease (OSD). | Osgood- schlatter - knee | Adolescent athletes 9-16 years of age | 61% expected athlete with OSD to be back to pre-injury RTP in less than 6 months  RTP:   - 97% load management - 87% pain intensity - 86% psychological factors e.g. social exclusion - 95% Social e.g. lack of support   Perceived importance  Load management   - Physio = important - Ortho = very unimportant - Tissue damage - Physio = very unimportant - Rheumatologists = very important - Pain at rest - Ortho = unimportant   Parents   - Can be both a barrier or a facilitator in rehabilitation - Performative parenting or pushy parents encouraging early RTA can create stress to the which has a negative effect on adolescents health - Early RTA and increased stress can lead to recurring symptoms or worsening progression of the disease. | The majority of HCPs in the current study perceived an optimistic prognosis and believed adolescents with OSD would RTP pain-free within 6 months; however, psychosocial aspects are potential barriers. |
| **Lower leg injuries** | | | | | | | | |
| Green, B.  McClelland, J. A.  Semciw, A. I.  Schache, A. G.  McCall, A.  Pizzari, T. | Qualtitative study – in- depath interviews | Specialist clinicians | 20 | To evaluate a select group of international experts current practices and perspectives regarding the assessment, management and prevention of calf muscle strain injuries | Calf muscle strain injuries | Adult professional athletes   - Football, Australian football, track and field, Olympic sports, rugby, ballet, cricket and collegiate sports. | RTP decisions based on:   - Cumulative recovery from rehabilitation - Psychological readiness - Key stakeholder consensus - An acceptable level of risk taking in account strategy, competition schedule, position   Checklist of criteria:   - Symptom resolution - Psychological readiness - Clinical signs and symptoms (palpation, pain VAS, ROM, strength) - Sports specific : strength-power, repeated tests - Training load reconditioning: volume, intensity, dynamic activity, mechanism of injury - Successful reintegration into training: ability to tolerate load and improve functionally | The clinicians use a structured phased approach based on clinical reasoning to diagnose, estimate prognosis, and manage calf muscle strain injuries. The phases are encouraged to extend beyond RTP time due to the risk of reinjury. The final RTP decision needs to be made with consensus amongst stakeholders and informed by clinical and athlete monitoring data. A generic injury prevention programme may not be possible for calf strains due to the range of calf demands and factors that predispose the athlete to injury. Management needs to be phased and individualised based on load and exercise selection. |
| **Foot and ankle injuries** | | | | | | | | |
| Aguilaniu, A.  Delvaux, F.  Schwartz, C.  Martens, G.  Forthomme, B.  Kaux, J-F.  Croisier, J-L. | Survey | Physicians  Physiotherapist | 212 | To compare the practice between physicians and physiotherapists. To analyse whether one of these groups use a quantitative (vs. qualitative) assessment of ankle strength more frequently. Finally, we investigated the consideration of strength for clinicians with or without Sports Medicine or Physiotherapy Edu- cation, respectively. | Ankle: Lateral ankle sprain | Active | 60% of physios consider ankle strength compared to 38% of physicians before RTP clearance after LAS. 7% and 9% of physicians and physiotherapists respectively use quantitative measures to assess ankle strength.  Sports medicine and sports physiotherapy education resulted in better use of quantitative assessment.  Isokinetic dynamometry, hand-held dynamometry or Myolux was used between 2%-10% whereas manual assessment 92% to 93%.  Eversion strength measured in high proportions followed by dorsiflexion, inversion and plantarflexion strength.  Physicians preferred testing isometric strength  Physiotherapists preferred testing concentric and eccentric strength.  Differences to determine readiness to RTP was mainly considered using bilateral comparisons. | Few physiotherapists and physicians consider ankle muscle strength including the use of quantitative measurements for deciding RTP after LAS. Ankle muscle strength is integrated into a holistic assessment and not a standalone criterion. Sports medicine and sports physiotherapy education enhance the consideration and use of quantitative ankle muscle strength assessment as well as over criteria necessary for RTP after LAS. |
| Johnson-Lynn, S.  Townshend, D. | Cross sectional online survey using open and closed questions | Healthcare professionals who regularly deal with sports injuries  -Orthopaedic surgeons  -Emergency medicine doctors  -Physiotherapists | 102 | Knowledge, confidence and experience of registrars in emergency medicine and orthopaedics versus consultants and physiotherapists of various levels of experience on 5 common injuries to the foot and ankle. | Foot and ankle trauma   - bimalleolar ankle fracture - 5th metatarsal fracture - lateral ankle ligament injury - navicular stress fracture - tendon Achilles rupture. | Not described | Survey asked 3 questions on knowledge   1. Bimalleolar fracture  - Time to RTR = average 9 weeks - Orthopaedic consultants in foot and ankle surgery (77%) and other specialties (94%) more likely to correctly estimate time to RTR after bimalleolar ankle fracture.  1. Bimalleolar fracture  - Time to return to work involving driving - Variability in advice: orthopaedic consultants (6-18 wk.), followed by registrars in orthopaedics and consultant foot and ankle surgeons (6-15 wk.).  1. Achilles tendon rupture  - Time to RTP - squash - Consultants in foot and ankle surgery (55%) and registrars in emergency medicine (55%) were most likely to correctly estimate time to return to squash following Achilles tendon rupture.   Specifics of designing rehabilitation programs:  Consultant foot and ankle surgeons and extended scope practitioners (ESP) scored higher on rehabilitation program design  As a whole practitioners in ankle and foot trauma, average 63% are confidence with regards to rehabilitation advice. The most confident group are the consultant foot and ankle doctors, followed by ESP. The least confident are the registrars in Trauma & Orthopaedics and Registrars Emergency Medicine. | Specialist orthopaedic surgeons and specialist physiotherapists had better rehabilitation knowledge of foot and ankle injuries compared to orthopaedic and emergency medicine registrars and more junior physiotherapists. Senior practitioners are more confident however this did not correlate to the knowledge of specific conditions. Registrars in emergency medicine and orthopaedics will benefit by being educated on sports injury rehabilitation using case-based teaching. |

Supplementary Table 6: AXIS checklist

| AXIS critical appraisal tool | | YES | NO | DO NOT KNOW / COMMENT |
| --- | --- | --- | --- | --- |
| Introduction | | | | |
| 1. | Were the aims/objectives of the study clear? |  |  |  |
| Methods | | | | |
| 2. | Was the study design appropriate for the stated aim(s)? |  |  |  |
| 3. | Was the sample size justified? |  |  |  |
| 4. | Was the target/reference population clearly defined? (Is it clear who the research was about?) |  |  |  |
| 5. | Was the sample frame taken from an appropriate population base so that it closely represented the target/reference population under investigation? |  |  |  |
| 6. | Was the selection process likely to select subjects/participants that were representative of the target/reference population under investigation? |  |  |  |
| 7. | Were measures undertaken to address and categorise non-responders? |  |  |  |
| 8. | Were the risk factor and outcome variables measured appropriate to the aims of the study? |  |  |  |
| 9. | Were the risk factor and outcome variables measured correctly using instruments/measurements that had been trialled, piloted, or published previously? |  |  |  |
| 10. | Is it clear what was used to determined statistical significance and/or precision estimates? (e.g., p values, CIs) |  |  |  |
| 11. | Were the methods (including statistical methods) sufficiently described to enable them to be repeated? |  |  |  |
| Results | | | | |
| 12. | Were the basic data adequately described? |  |  |  |
| 13 | Does the response rate raise concerns about non-response bias? |  |  |  |
| 14. | If appropriate, was information about non-responders described? |  |  |  |
| 15. | Were the results internally consistent? |  |  |  |
| 16. | Were the results for the analyses described in the methods, presented? |  |  |  |
| Discussion | | | | |
| 17. | Were the authors’ discussions and conclusions justified by the results? |  |  |  |
| 18. | Were the limitations of the study discussed? |  |  |  |
| Other | | | | |
| 19. | Were there any funding sources or conflicts of interest that may affect the authors’ interpretation of the results? |  |  |  |
| 20. | Was ethical approval or consent of participants attained? |  |  |  |

## Supplementary Table 7: AXIS results – Cross sectional studies

Answers: Yes | No | Do not know/comment = DNK – do not know, ND – not described, NC – not clear, NS – not stated

| **Author/s** | 1. Were the aims/objectives of the study clear? | 2.Was the study design appropriate for the stated aim(s)? | 3. Was the sample size justified? | 4. Was the target/reference population clearly defined? (Is it clear who the research was about?) | 5. Was the sample frame taken from an appropriate population base so that it closely represented the target/ reference population under investigation? | 6. Was the selection process likely to select subjects/ participants that were representative of the target/ reference population under investigation? | 7. Were measures undertaken to address and categorise non-responders? | 8. Were the risk factor and outcome variables measured appropriate to the aims of the study? | 9. Were the risk factor and outcome variables measured correctly using instruments / measurements that had been trialled, piloted, or published previously? | 10. Is it clear what was used to determined statistical significance and/or precision estimates? (e.g., p values, CIs) | 11. Were the methods (including statistical methods) sufficiently described to enable them to be repeated? | 12. Were the basic data adequately described? | 13. Does the response rate raise concerns about non-response bias? | 14. If appropriate, was information about non-responders described? | 15. Were the results internally consistent? | 16. Were the results for the analyses described in the methods, presented? | 17. Were the authors discussions and conclusions justified by the results? | 18. Were the limitations of the study discussed? | 19. Were there any funding sources or conflicts of interest that may affect the authors interpretation of the results? | 20. Was ethical approval or consent of participants attained? | **Total** |
| --- | --- | --- | --- | --- | --- | --- | --- | --- | --- | --- | --- | --- | --- | --- | --- | --- | --- | --- | --- | --- | --- |
| **Medical doctors** | | | | | | | | | | | | | | | | | | | | | |
| **Various musculoskeletal injury scenarios** | | | | | | | | | | | | | | | | | | | | | |
| Anderson, L. C.  Gerrard, D. F. | Yes | Yes | DNK | Yes | Yes | Yes | Yes | Yes | DNK | Yes | Yes | Yes | DNK | Yes | DNK | Yes | Yes | Yes | No | Yes | 16 |
| Hobusch, G. M., Keusch, F.,  Tsuchiya, H.,  Joyce, M.,  Windhager, R. | Yes | Yes | Yes | Yes | Yes | Yes | Yes | Yes | ND | Yes | Yes | Yes | DNK | No | DNK | Yes | Yes | Yes | No | No | 16 |
| Schrock, J. B., Carver, T. J., Kraeutler, M. J., McCarty, E. C. | Yes | Yes | Yes | Yes | Yes | Yes | Yes | Yes | Yes | Yes | Yes | Yes | No | No | DNK | Yes | Yes | Yes | Yes | Yes | 16 |
| **Spine injuries** | | | | | | | | | | | | | | | | | | | | | |
| Abla, A. A., Maroon, J. C., Lochhead, R., Sonntag, V. K., Maroon, A., Field, M. | Yes | Yes | Yes | Yes | Yes | Yes | ND | Yes | ND | Yes | Yes | Yes | ND | ND | ND | Yes | Yes | ND | No | Yes | 14 |
| Backer, H. C.  Johnson, M. A.  Hanlon, J.  Chan, P.  Turner, P.  Cunningham, J. | yes | yes | yes | yes | yes | yes | ND | yes | yes | yes | yes | yes | No | no | ND | yes | yes | no | yes | no | 15 |
| France, J.C., Karsy, M., Harrop, J.S., Dailey, A.T. | Yes | Yes | No | Yes | No | Yes | No | Yes | ND | Yes | Yes | No | Yes | ND | ND | Yes | Yes | Yes | No | ND | 12 |
| Ho, D.  Du, J. Y.  Erkilinc, M.  Glotzbecker, M. P.  Mistovich, R. J. | Yes | Yes | Yes | Yes | Yes | Yes | ND | Yes | ND | Yes | Yes | Yes | Yes | Yes | ND | Yes | Yes | Yes | No | Yes | 17 |
| Morganti, C.  Sweeney, C.A.  Albanese, S.A.  Burak, C.  Hosea, T.  Connolly, P.J. | Yes | Yes | Yes | Yes | Yes | Yes | ND | Yes | ND | Yes | Yes | No | Yes | No | ND | Yes | Yes | No | No | ND | 13 |
| Ukogu, C.  Bienstock, D.  Ferrer, C.  Zubizarreta, N.  McAnany S.  Chaudhary, S. B.  Iatridis, J. C.  Hecht, A. C. | Yes | Yes | Yes | Yes | Yes | Yes | Yes | Yes | Yes | Yes | Yes | Yes | No | Yes | NS | Yes | Yes | Yes | No | Yes | 19 |
| **Thorax** | | | | | | | | | | | | | | | | | | | | | |
| McAdams, T. R.  Deimel, J. F.  Ferguson, J.  Beamer, B. S.  Beaulieu, C. F. | Yes | ND | Yes | Yes | ND | No | ND | ND | No | ND | Yes | No | ND | ND | ND | ND | Yes | No | ND | Yes | 6 |
| **Shoulder injuries** | | | | | | | | | | | | | | | | | | | | | |
| Golant, A.  Christoforou, D.  Zuckerman, J. D.  Kwon, Y. W. | Yes | Yes | Yes | Yes | Yes | Yes | ND | Yes | No | Yes | Yes | Yes | No | ND | ND | Yes | Yes | Yes | No | Yes | 16 |
| Moore, David M.  Hurley, Eoghan T.  Mullett, Hannan | Yes | Yes | Yes | Yes | Yes | Yes | ND | Yes | ND | Yes | Yes | Yes | No | ND | ND | Yes | Yes | Yes | No | ND | 15 |
| Sharareh, B.  Edwards, T. B.  Shah, A.  Shybut, T. | Yes | Yes | Yes | Yes | Yes | Yes | Yes | Yes | ND | Yes | Yes | Yes | Yes | Yes | ND | Yes | Yes | Yes | No | Yes | 17 |
| **Elbow injuries** | | | | | | | | | | | | | | | | | | | | | |
| Sambare, N. D.  Chalmers, P. N.  Camp, C. L.  Bowman, E. N.  Erickson, B. J.  Sciascia, A.  Freehill, M. T.  Smith, M. V. | yes | yes | yes | yes | yes | yes | yes | yes | no | yes | yes | yes | yes | yes | yes | yes | yes | yes | no | yes | 19 |
| **Hand and wrist injuries** | | | | | | | | | | | | | | | | | | | | | |
| Dy, C. J.  Khmelnitskaya, E.  Hearns, K. A.  Carlson, M. G. | Yes | Yes | No | Yes | Yes | Yes | No | Yes | Yes | Yes | Yes | Yes | DNK | No | NS | Yes | Yes | Yes | ND | Yes | 14 |
| **Hip, groin and pelvic injuries** | | | | | | | | | | | | | | | | | | | | | |
| Domb, B.G.  Stake, C.E.  Finch, N.A.  Cramer, L. | Yes | Yes | No | Yes | ND | Yes | No | Yes | No | No | Yes | Yes | No | N/A | ND | Yes | Yes | Yes | Yes | ND | 11 |
| Vu-Han, T.  Hardt, S.  Ascherl, R.  Gwinner, C.  Perka, C. | Yes | Yes | Yes | Yes | Yes | Yes | DNK | Yes | Yes | Yes | Yes | Yes | No | No | DNK | Yes | Yes | Yes | No | Yes | 17 |
| **Knee injuries** | | | | | | | | | | | | | | | | | | | | | |
| Arliani, G.G.  Pereira, V.L.  Leao, R.G.  Lara, P.S.  Ejnisman, B.  Cohen, M. | Yes | Yes | Yes | Yes | Yes | Yes | N/A | Yes | Yes | Yes | Yes | Yes | No | N/A | NS | Yes | Yes | Yes | No | Yes | 17 |
| Bakowski, P.  Bakowski-Zywicka, K.  Pointek, T. | Yes | Yes | Yes | Yes | Yes | Yes | N/A | Yes | Yes | Yes | Yes | Yes | No | N/A | ND | Yes | Yes | Yes | No | Yes | 17 |
| Betsch, M.,  Darwich, A.,  Chang, J.,  Whelan, D.,  Ogilvie-Harris, D.,  Chahal, J.,  Theodoropoulos, J. | Yes | Yes | ND | Yes | Yes | Yes | ND | Yes | Yes | Yes | Yes | Yes | No | ND | ND | Yes | Yes | Yes | No | Yes | 16 |
| Coskunsu, D.  Bayrakci Tunay, V.  Akgun, I. | Yes | Yes | No | Yes | Yes | No | Yes | No | Yes | Yes | Yes | Yes | No | ND | Yes | Yes | Yes | No | NS | ND | 13 |
| Ebert, J. R.  Webster, K.E.  Edwards, P. K.  Joss, B. K.  D’Alessandro, P.  Janes, G.  Annear, P. | Yes | Yes | Yes | Yes | Yes | Yes | No | Yes | No | No | Yes | Yes | No | No | ND | Yes | Yes | No | No | Yes | 14 |
| Erickson, B. J.  Harris, J. D.  Fillingham, Y. A.  Cvetanovich, G. L.  Bush-Joseph, C.  Cole, B. J.  Bach, B. R.  Verma, N.N. | Yes | Yes | Yes | Yes | Yes | Yes | Yes | Yes | Yes | Yes | Yes | Yes | No | No | NS | Yes | Yes | Yes | No | Yes | 18 |
| Erickson B. J.  Harris, J. D.  Fillingham, Y. A.  Frank, R. M.  Bush-Joseph, C. A.  Bach Jr, B. R.  Cole, B. J.  Verma, N. N. | Yes | Yes | Yes | Yes | Yes | Yes | ND | Yes | No | Yes | Yes | Yes | No | Yes | NS | Yes | Yes | Yes | Yes | Yes | 16 |
| Farber, J.  Harris, J.D.  Kolstad, K.  McCulloch, P.C. | Yes | Yes | Yes | Yes | Yes | Yes | Yes | Yes | Yes | Yes | Yes | Yes | No | N/A | ND | yes | Yes | Yes | No | Yes | 18 |
| Feller, J. A.  Cooper, R.  Webster, K. E. | Yes | Yes | Yes | Yes | Yes | Yes | ND | Yes | No | Yes | Yes | Yes | No | No | NS | Yes | Yes | DNK | ND | ND | 13 |
| Glattke, K.E.,  Tummala, S.V., Goldberg, B.,  Menzer, H.,  Chhabra, A. | Yes | Yes | ND | Yes | Yes | Yes | DNK | Yes | Yes | Yes | Yes | Yes | DNK | ND | ND | Yes | Yes | Yes | No | Yes | 15 |
| Grassi, A.  Vascellari, A.  Combi, A.  Tomaello, L.  Canatas, G.L.  Zaffagnini, S. | Yes | Yes | Yes | Yes | Yes | Yes | Yes | Yes | Yes | Yes | Yes | Yes | No | No | ND | Yes | Yes | Yes | No | Yes | 17 |
| Mahnik, A.  Mahnik, S.  Dimnjakovic, D.  Curic, S.  Smoljanovic, T.  Bojanic, I. | yes | yes | Yes | Yes | Yes | Yes | yes | Yes | DNK | Yes | Yes | Yes | DNK | No | DNK | Yes | Yes | Yes | DNK | DNK | 14 |
| Marshall, N.E.  Keller, R.A  Dines, J.  Bush-Joseph, C.  Limpisvasti, O. | Yes | Yes | Yes | Yes | Yes | Yes | ND | Yes | No | Yes | Yes | Yes | No | No | NS | Yes | Yes | Yes | No | Yes | 16 |
| McRae, S. M.  Chahal, J.  Leiter, J. R.  Marx, R. G.  MacDonald, P. B. | Yes | Yes | Yes | Yes | Yes | Yes | Yes | Yes | Yes | Yes | Yes | Yes | No | Yes | ND | Yes | Yes | Yes | No | Yes | 19 |
| Pandey, V.  Madi, S.  Thonse, C.  Joseph, C.  Rajan, D.  Varughese, J.  Thilak, J.  Jayaprasad, P. S.  Acharya, K.  Ramamurthy, K. G.  Reddy, R.  Amravathi, R.  Rao, S.  Gangavarapu, S.  Srinivas, M.  Jose, S.  Sundararjan, S. R. | Yes | Yes | Yes | Yes | Yes | Yes | Yes | Yes | No | Yes | Yes | Yes | DNK | Yes | Yes | Yes | Yes | Yes | No | No | 17 |
| Petersen, W.  Zantop, T. | Yes | Yes | Yes | Yes | Yes | Yes | No | Yes | No | No | Yes | Yes | No | Yes | NS | Yes | Yes | Yes | No | Yes | 16 |
| Sherman, S. L.  Calcei, J.  Ray, T.  Magnussen, R. A.  Musahl, V.  Kaeding, C. C.  Clatworthy, M.  Bergfeld, J. A.  Arnold, M. P. | Yes | Yes | Yes | Yes | Yes | Yes | Yes | Yes | DNK | Yes | Yes | Yes | No | N/A | DNK | Yes | Yes | Yes | Yes | DNK | 15 |
| Thaler, M.  Khosravi, I.  Putzer, D.  Michael T.  Hirschmann, M. T.  Kort, N.  Tandogan, R. N. Liebensteiner, M. | Yes | Yes | Yes | Yes | Yes | Yes | No | Yes | ND | Yes | Yes | Yes | No | No | NS | Yes | Yes | Yes | No | Yes | 16 |
| Vascellari, A.  Grassi, A.  Combi, A.  Tomaello, L.  Canata, G. L.  Zaffagnini, S.  Sigascot Sports Committee | Yes | Yes | Yes | Yes | Yes | Yes | Yes | Yes | DNK | Yes | Yes | Yes | DNK | No | DNK | Yes | Yes | Yes | No | Yes | 16 |
| **Ankle and foot injuries** | | | | | | | | | | | | | | | | | | | | | |
| Aguilaniu, A.  Croisier, J. L.  Schwartz, C.  Dardenne, N.  D'Hooghe, P.  Martens, G.  Collin, R.  Kaux, J. F. | Yes | Yes | Yes | Yes | Yes | Yes | ND | Yes | Yes | Yes | Yes | Yes | ND | ND | ND | Yes | Yes | Yes | No | Yes | 16 |
| Beck, J.J., Carpenter, C.M., West, N., Sabatino, M.J., Ellis, H.B. | Yes | Yes | Yes | Yes | Yes | Yes | Yes | Yes | ND | Yes | Yes | Yes | DNK | ND | DNK | Yes | Yes | Yes | No | No | 15 |
| Dams, O.C.  Van den Akker-Scheek, I.  Diercks, R.L.  Wendt, K.W.  Zwerver, J.  Reininga, I.H.F | Yes | Yes | Yes | Yes | Yes | Yes | Yes | Yes | Yes | Yes | Yes | Yes | No | Yes | NS | Yes | Yes | NS | No | Yes | 18 |
| Vertullo, C.  Nunley, J. A. | Yes | Yes | Yes | No | No | Yes | No | Yes | No | Yes | Yes | Yes | No | Yes | NS | Yes | Yes | Yes | No | Yes | 15 |
| Yokoe, T.  Tajima, T.  Yamaguchi, N.  Morita, Y.  Chosa, E. | Yes | Yes | Yes | Yes | Yes | Yes | DNK | Yes | No | Yes | Yes | Yes | DNK | Yes | Yes | Yes | Yes | Yes | No | Yes | 17 |
| **Physiotherapists** | | | | | | | | | | | | | | | | | | | | | |
| **Generalised RTP** | | | | | | | | | | | | | | | | | | | | | |
| Silva, Anderson A  Bittencourt, Natália FN  Mendonça, Luciana M  Tirado, Marcella G  Sampaio, Rosana F  Fonseca, Sérgio T | Yes | Yes | Yes | Yes | Yes | Yes | DNK | Yes | DNK | Yes | Yes | Yes | DNK | DNK | DNK | Yes | Yes | Yes | DNK | Yes | 14 |
| **Shoulder injuries** | | | | | | | | | | | | | | | | | | | | | |
| Brindisino, Fabrizio  De Santis, Andrea  Rossettini, Giacomo  Pellicciari, Leonardo  Filipponi, Marco  Rollo, Giuseppe  Gibson, Jo | Yes | Yes | No | Yes | Yes | Yes | No | Yes | Yes | Yes | Yes | Yes | ND | ND | Yes | Yes | Yes | Yes | No | Yes | 16 |
| Brindisino, F.  Lorusso, M.  Usai, M.  Pellicciari, L.  Marruganti, S.  Salomon, M. | yes | yes | yes | yes | yes | yes | yes | yes | yes | yes | yes | yes | no | yes | unsure | yes | yes | yes | no | yes | 19 |
| Gauthier, M. L.  Unverzagt, C. A.  Mendonça, L. M.  Seitz, A. L. | yes | Yes | yes | yes | Yes | yes | yes | yes | yes | yes | yes | yes | no | yes | yes | yes | yes | yes | no | yes | **20** |
| **Hamstring injuries** | | | | | | | | | | | | | | | | | | | | | |
| Nasser, A. M.  Pizzari, T.  Grimaldi, A.  Vicenzino, B.  Rio, E.  Semciw, A. I. | Yes | Yes | Yes | Yes | Yes | Yes | N/A | Yes | Yes | Yes | Yes | Yes | N/A | Yes | DNK | Yes | Yes | Yes | No | Yes | 17 |
| Valente, H. G.  Oliveira, R. R.  Baroni, B. M. | yes | yes | yes | yes | yes | yes | yes | yes | yes | yes | yes | yes | no | yes | yes | yes | yes | yes | no | yes | 20 |
| **Knee injuries** | | | | | | | | | | | | | | | | | | | | | |
| Alshehri, Y. S.  Aljohani, M. M. A.  Alzahrani, H.  Alzhrani, M.  Alkhathami, K. M.  Alshahrani, A.  Khaled, O. A. | yes | yes | yes | yes | yes | yes | yes | yes | yes | no | yes | yes | no | yes | ND | yes | yes | yes | no | yes | 18 |
| Aquino, C.F.  Ocarino, J. M.  Cardosos, V. A.  Resende, R. A.  Souza, T. R.  Rabelo, L. M.  Fonseca, S. T. | Yes | Yes | No | Yes | Yes | Yes | No | Yes | Yes | Yes | Yes | Yes | DNK | Yes | DNK | Yes | Yes | Yes | No | Yes | 16 |
| Fausett, W. A.  Reid, D. A.  Larmer, P. J. | Yes | Yes | ND | Yes | Yes | Yes | ND | Yes | Yes | Yes | Yes | Yes | DNK | ND | ND | Yes | Yes | Yes | No | Yes | 15 |
| Greenberg, E. M.  Greenberg, E.T.  Albaugh, J.  Storey, E.  Ganley, T.J. | Yes | Yes | Yes | Yes | Yes | Yes | Yes | Yes | No | Yes | Yes | Yes | ND | ND | NS | Yes | Yes | Yes | No | Yes | 16 |
| Kaye, J. A.  Spence, D.  Alexanders, J. | Yes | Yes | Yes | Yes | Yes | Yes | Yes | Yes | Yes | Yes | Yes | Yes | No | NA | Yes | Yes | Yes | Yes | No | Yes | 19 |
| Korakakis, V.  Kotsifaki, A.  Korakaki, A.  Karanasios, S.  Whiteley, R. | Yes | Yes | Yes | Yes | Yes | DNK | DNK | Yes | Yes | Yes | Yes | Yes | No | No | Yes | Yes | Yes | Yes | No | Yes | 17 |
| Mendonca, L. D. M.  Bittencourt, N. F. N.  Alves, L. E. M.  Resende, R. A.  Serrão, F. V. | Yes | Yes | Yes | Yes | Yes | Yes | Yes | Yes | Yes | Yes | Yes | Yes | ND | No | NS | Yes | Yes | Yes | No | Yes | 17 |
| Pulver, M.  Hilfiker, R.  Bizzini, M.  Mathieu, N.  Meyer, S.  Allet, L. | yes | yes | yes | yes | yes | yes | yes | yes | yes | yes | yes | yes | ND | no | ND | yes | yes | yes | no | yes | 17 |
| Tondelli, E.  Feroldi, A.  García, F.  Meza, F.  Dingenen, B. | Yes | Yes | Yes | Yes | Yes | Yes | Yes | Yes | Yes | Yes | Yes | Yes | No | yes | ND | Yes | yes | yes | No | Yes | 19 |
| van Melick, N.  Hoogeboom, T. J.  Pronk, Y.  Rutten, B.  van Tienen, T. G.  Nijhuis-van der Sanden, M. W. G.  van Cingel, R. E. H. | Yes | Yes | No | Yes | Yes | Yes | N/A | Yes | Yes | Yes | Yes | Yes | N/A | N/A | N/A | Yes | Yes | Yes | No | Yes | 15 |
| Von Aesch, A. V.  Perry, M.  Sole, G. | Yes | Yes | No | Yes | ND | ND | ND | Yes | Yes | Yes | Yes | Yes | No | No | NS | Yes | Yes | Yes | No | Yes | 14 |
| Witjes, S.  Hoorntje, A.  Koenraadt, K. L. M.  Goossens, P.  KerKHoffs, G. M. MJ.  van Geenen, R. C. I. | Yes | Yes | Yes | Yes | Yes | Yes | Yes | Yes | ND | Yes | Yes | Yes | No | Yes | ND | Yes | Yes | Yes | No | ND | 17 |
| **Rehabilitation Specialists** | | | | | | | | | | | | | | | | | | | | | |
| **Hamstring injuries** | | | | | | | | | | | | | | | | | | | | | |
| Di Trani Lobacz, A.  Glutting, J.  Kaminski, T. W. | Yes | Yes | yes | Yes | Yes | Yes | ND | Yes | Yes | Yes | Yes | Yes | No | Yes | Yes | Yes | Yes | Yes | NDis | Yes | 18 |
| McVeigh, F.  Pack, S. M. | Yes | Yes | Yes | Yes | Yes | Yes | Yes | Yes | Yes | Yes | Yes | Yes | NC | Yes | ND | Yes | Yes | Yes | NC | Yes | 17 |
| **Multidisciplinary Team** | | | | | | | | | | | | | | | | | | | | | |
| **Generalised RTP practices** | | | | | | | | | | | | | | | | | | | | | |
| Barrette, A.  Harman, K. | Yes | Yes | Yes | Yes | Yes | Yes | DNK | Yes | Yes | Yes | Yes | Yes | DNK | No | DNK | Yes | Yes | Yes | No | Yes | 16 |
| Beardmore, A.L.  Handcock, P. J.  Rehrer, N. J. | Yes | Yes | Yes | Yes | Yes | Yes | Yes | Yes | ND | ND | Yes | Yes | No | ND | ND | Yes | Yes | Yes | No | Yes | 16 |
| Boudier-Reveret, M.  Mazer, B.  Feldman, D. E.  Shrier, I. | Yes | Yes | Yes | Yes | Yes | Yes | No | Yes | Yes | Yes | Yes | Yes | No | No | ND | Yes | Yes | Yes | No | Yes | 17 |
| Chen, Y.  Buggy, C.  Kelly, S. | Yes | Yes | Yes | Yes | Yes | Yes | ND | Yes | Yes | Yes | Yes | Yes | DNK | ND | ND | Yes | Yes | No | No | Yes | 15 |
| Geldenhuys, A. G.  Burgess, T.  Roche, S.  Hendricks, S. | Yes | yes | yes | yes | yes | yes | yes | yes | yes | yes | yes | yes | no | yes | yes | yes | yes | yes | no | yes | 20 |
| Hess, C. W.  Meyer, B. B. | Yes | Yes | Yes | Yes | Yes | Yes | NA | Yes | Yes | Yes | Yes | Yes | NA | NA | DNK | Yes | Yes | Yes | No | Yes | 16 |
| Horan, D.  Kelly, S.  Hägglund, M.  Blake, C.  Roe, M.  Delahunt, E. | yes | yes | yes | yes | yes | yes | yes | yes | no | yes | yes | yes | no | yes | yes | yes | yes | yes | no | yes | 19 |
| Mazer, B.  Shrier, I.  Ehrmann Feldman, D.  Swaine, B.  Majnemer, A.  Kennedy, E.  Chilingaryan, G. | yes | yes | yes | yes | yes | yes | yes | yes | yes | yes | yes | yes | No | yes | yes | yes | yes | yes | no | yes | 20 |
| Müller, P. O.  Helbling, M.  Verhagen, E.  Spörri, J.  Bolling, C. | yes | yes | yes | yes | yes | yes | yes | yes | yes | yes | yes | yes | no | yes | unsure | yes | yes | yes | no | yes | 19 |
| Read, D.  Rosenbloom, C. | yes | yes | yes | yes | yes | yes | yes | yes | no | yes | yes | yes | no | yes | unsure | yes | yes | yes | no | yes | 18 |
| Read, P. J.  Jimenez, P.  Oliver, J. L.  Lloyd, R. S. | Yes | Yes | Yes | Yes | Yes | Yes | ND | Yes | Yes | Yes | Yes | Yes | No | No | ND | Yes | Yes | No | No | Yes | 16 |
| Riendeau, C.  Parent-Houle, V.  Lebel-Gabriel, M. E.  Gauvin, P.  Liu le, Y.  Pearson, I.  Hunt, M. R. | Yes | Yes | Yes | Yes | Yes | Yes | No | Yes | Yes | Yes | Yes | Yes | No | N/A | DNK | Yes | Yes | Yes | No | Yes | 17 |
| Shultz, R. Bido, J.  Shrier, I.  Meeuwisse, W. H.  Garza, D.  Matheson, G. O. | Yes | Yes | Yes | Yes | Yes | Yes | Yes | Yes | Yes | Yes | Yes | Yes | No | Yes | ND | Yes | Yes | Yes | No | Yes | 19 |
| Shrier, I.  Safai, P.  Charland, L. | Yes | Yes | Yes | Yes | Yes | Yes | Yes | Yes | Yes | Yes | Yes | Yes | No | Yes | ND | Yes | Yes | Yes | No | Yes | 19 |
| Shrier, I.  Serner, A.  Wangensteen, A.  Steele, R. J.  Weir, A. | Yes | Yes | DNK | Yes | Yes | Yes | Yes | Yes | Yes | Yes | Yes | Yes | DNK | Yes | DNK | Yes | Yes | Yes | No | Yes | 17 |
| Yeomans, C.  Comyns, T. M.  Cahalan, R.  Warrington, G. D.  Harrison, A. J.  Hayes, K.  Lyons, M.  Campbell, M. J.  Kenny, I. C. | Yes | Yes | Yes | Yes | Yes | Yes | Yes | Yes | Yes | Yes | Yes | Yes | No | DNK | DNK | Yes | Yes | Yes | No | Yes | 18 |
| **Shoulder injuries** | | | | | | | | | | | | | | | | | | | | | |
| Maher, N.  Willmore, E.  Bateman, M.  Blacknall, J.  Chester, R.  Horsley, I.  Gibson, J.  O' Sullivan J  Jaggi, A. | yes | yes | yes | yes | yes | yes | Yes | yes | yes | yes | yes | yes | no | yes | ND | yes | yes | yes | no | yes | 19 |
| **Hip injuries** | | | | | | | | | | | | | | | | | | | | | |
| Worner, T.  Thorborg, K.  Moksnes, H.  Eek, F. | Yes | Yes | Yes | Yes | Yes | Yes | Yes | Yes | Yes | Yes | Yes | Yes | DNK | ND | NS | Yes | Yes | Yes | No | Yes | 17 |
| **Hamstring injuries** | | | | | | | | | | | | | | | | | | | | | |
| Balcı, Aydın  Ülkar, Bülent | Yes | Yes | Yes | Yes | Yes | Yes | Yes | Yes | No | Yes | Yes | Yes | No | ND | NS | Yes | Yes | Yes | No | Yes | 17 |
| Dunlop, G.  Ardern, C.L.  Andersen, T.E.  Lewin, C.  Dupont, G.  Ashworth, B.  O’Driscoll, G.  Rolls, A.  Brown, S.  McCall, A. | Yes | Yes | Yes | Yes | Yes | Yes | Yes | Yes | Yes | Yes | Yes | Yes | DNK | ND | Yes | Yes | Yes | Yes | No | Yes | 18 |
| **Knee injuries** | | | | | | | | | | | | | | | | | | | | | |
| Ebert, J. R.  Webster, K.E.  Edwards, P. K.  Joss, B. K.  D’Alessandro, P.  Janes, G.  Annear, P. | Yes | Yes | No | Yes | Yes | Yes | No | Yes | No | No | Yes | Yes | No | No | ND | Yes | Yes | Yes | No | Yes | 14 |
| Lambert, C.  Rotzmann, R.  Ellermann, A.  Carvalho, M.  Akoto, R.  Wafaisade, A.  Lambert, M. | Yes | Yes | Yes | Yes | Yes | Yes | Yes | Yes | Yes | Yes | Yes | Yes | ND | N/A | ND | Yes | Yes | Yes | No | Yes | 18 |
| Lyng. K. D.  Rathleff, M. S.  Dean, B. J. F.  Kluzek, S.  Holden, S. | Yes | Yes | Yes | Yes | Yes | Yes | No | Yes | Yes | Yes | Yes | Yes | DNK | ND | NS | Yes | Yes | Yes | No | Yes | 16 |
| **Lower leg injuries** | | | | | | | | | | | | | | | | | | | | | |
| Green, B.  McClelland, J. A.  Semciw, A. I.  Schache, A. G.  McCall, A.  Pizzari, T. | Yes | Yes | Yes | Yes | Yes | Yes | Yes | Yes | Yes | Yes | Yes | Yes | DNK | DNK | DNK | Yes | Yes | Yes | No | Yes | 17 |
| **Ankle and foot injuries** | | | | | | | | | | | | | | | | | | | | | |
| Aguilaniu, A.  Delvaux, F.  Schwartz, C.  Martens, G.  Forthomme, B.  Kaux, J-F.  Croisier, J-L. | yes | Yes | yes | yes | yes | yes | yes | yes | yes | yes | yes | yes | no | yes | ND | yes | yes | yes | no | yes | 19 |
| Johnson-Lynn, S.  Townshend, D. | Yes | Yes | Yes | Yes | Yes | Yes | Yes | Yes | Yes | Yes | Yes | Yes | No | Yes | DNK | Yes | Yes | Yes | DNK | DNK | 17 |

##
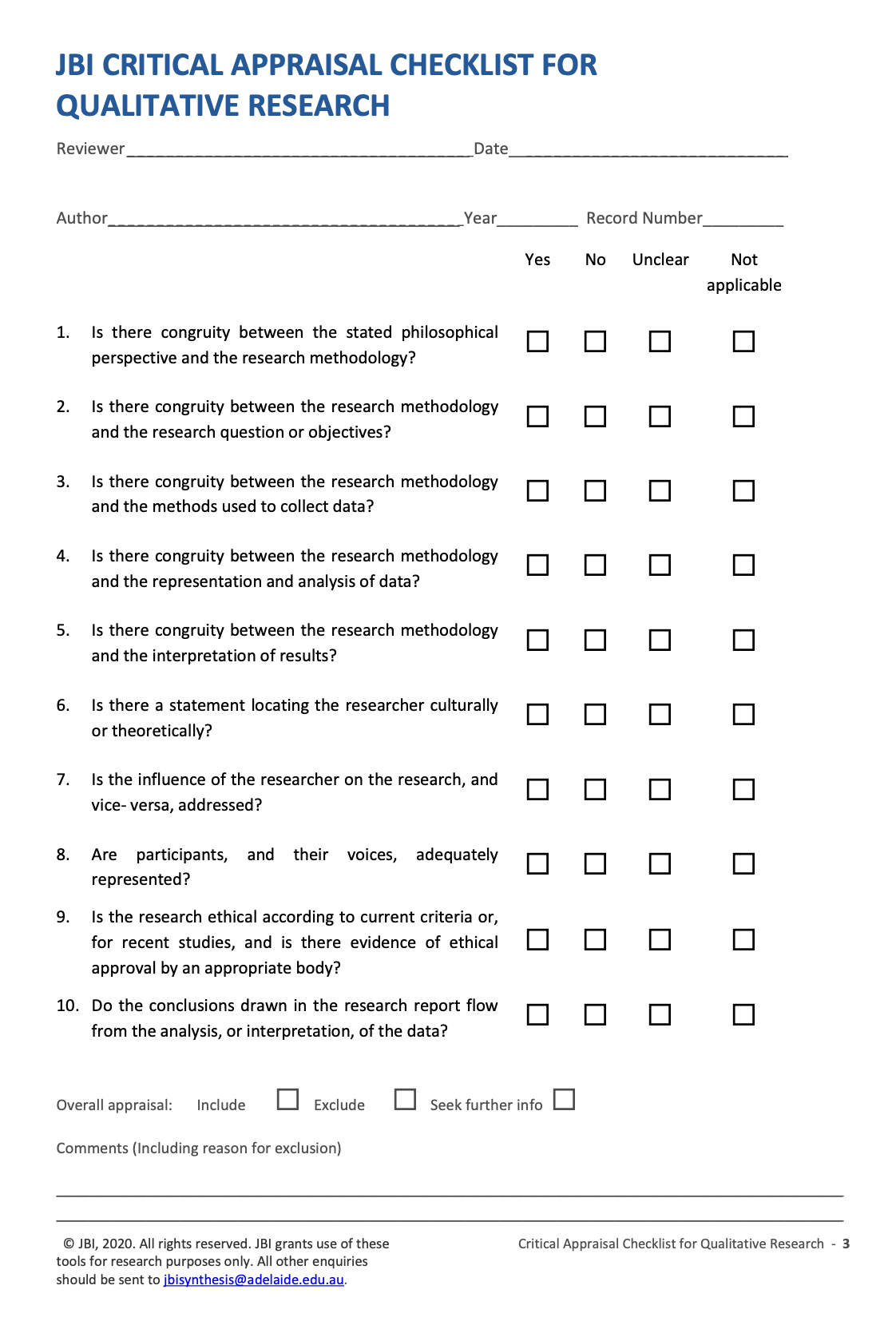
 Supplementary Table 8: JBI for Qualitative Studies

## Supplementary Table 9: JBI results – Qualitative studies

| **Author** | 1.Is there congruity between the stated philosophical perspective and the research methodology? | 2.Is there congruity between the research methodology and the research question or objectives? | 3. Is there congruity between the research methodology and the methods used to collect data? | 4. Is there congruity between the research methodology and the representation and analysis of data? | 5. Is there congruity between the research methodology and the interpretation of results? | 6.Is there a statement locating the researcher culturally or theoretically? | 7.Is the influence of the researcher on the research, and vice-versa, addressed? | 8. Are participants, and their voices, adequately represented? | 9.Is the research ethical according to current criteria or, for recent studies, and is there evidence of ethical approval by an appropriate body? | 10. Do the conclusions drawn in the research report flow from the analysis, or interpretation, of the data? | **Score** |
| --- | --- | --- | --- | --- | --- | --- | --- | --- | --- | --- | --- |
| **Medical doctors** | | | | | | | | | | | |
| Betsch, M.,  Darwich, A.,  Chang, J.,  Whelan, D.,  Ogilvie-Harris, D.,  Chahal, J.,  Theodoropoulos, J. | Yes | Yes | Yes | Yes | Yes | Unclear | Yes | Yes | Yes | Yes | 9/10 |
| **Physiotherapists** | | | | | | | | | | | |
| Nasser, A. M., Pizzari, T., Grimaldi, A., Vicenzino, B., Rio, E., Semciw, A. I. | Yes | Yes | Yes | Yes | Yes | Yes | Yes | Yes | Yes | Yes | 10/10 |
| Kaye, J. A.  Spence, D.  Alexanders, J. | Yes | Yes | Yes | Yes | Yes | Yes | Yes | Yes | Yes | Yes | 10/10 |
| Von Aesch, A. V., Perry, M., Sole, G. | Yes | Yes | Yes | Yes | Yes | Yes | Yes | Yes | Yes | Yes | 10/10 |
| **Rehabilitation specialists** | | | | | | | | | | | |
| McVeigh, F.  Pack, S. M. | Yes | Yes | Yes | Yes | Yes | Yes | Yes | Yes | Yes | Yes | 10/10 |
| **Multidisciplinary team** | | | | | | | | | | | |
| Barrette, A.  Harman, K. | Yes | Yes | Yes | Yes | Yes | Yes | Yes | Yes | Yes | Yes | 10/10 |
| Chen, Y.  Buggy, C.  Kelly, S. | Yes | Yes | Yes | Yes | Yes | Unclear | Yes | Yes | Yes | Yes | 9/10 |
| Hess, C. W.  Meyer, B. B. | Yes | Yes | Yes | Yes | Yes | Yes | Yes | Yes | Yes | Yes | 10/10 |
| Horan, D.  Kelly, S.  Hägglund, M.  Blake, C.  Roe, M.  Delahunt, E. | Yes | Yes | Yes | Yes | Yes | Yes | Yes | Yes | Yes | Yes | 10/10 |
| Read, D.  Rosenbloom, C. | Yes | Yes | Yes | Yes | Yes | Yes | Yes | Yes | Yes | Yes | 10/10 |
| Riendeau, C.  Parent-Houle, V.  Lebel-Gabriel, M. E.  Gauvin, P.  Liu le, Y.  Pearson, I.  Hunt, M. R. | Yes | Yes | Yes | Yes | Yes | Yes | Yes | Yes | Yes | Yes | 10/10 |
| Lyng. K. D., Rathleff, M. S., Dean, B. J. F., Kluzek, S., Holden, S. | Yes | Yes | Yes | Yes | Yes | Unclear | Unclear | Yes | Yes | Yes | 8/10 |
| Green, B.  McClelland, J. A.  Semciw, A. I.  Schache, A. G.  McCall, A.  Pizzari, T. | Yes | Yes | Yes | Yes | Yes | Yes | Yes | Yes | Yes | Yes | 10/10 |
| Müller, P. O.  Helbling, M.  Verhagen, E.  Spörri, J.  Bolling, C. | Yes | Yes | Yes | Yes | Yes | Yes | Yes | Yes | Yes | Yes | 10/10 |

##
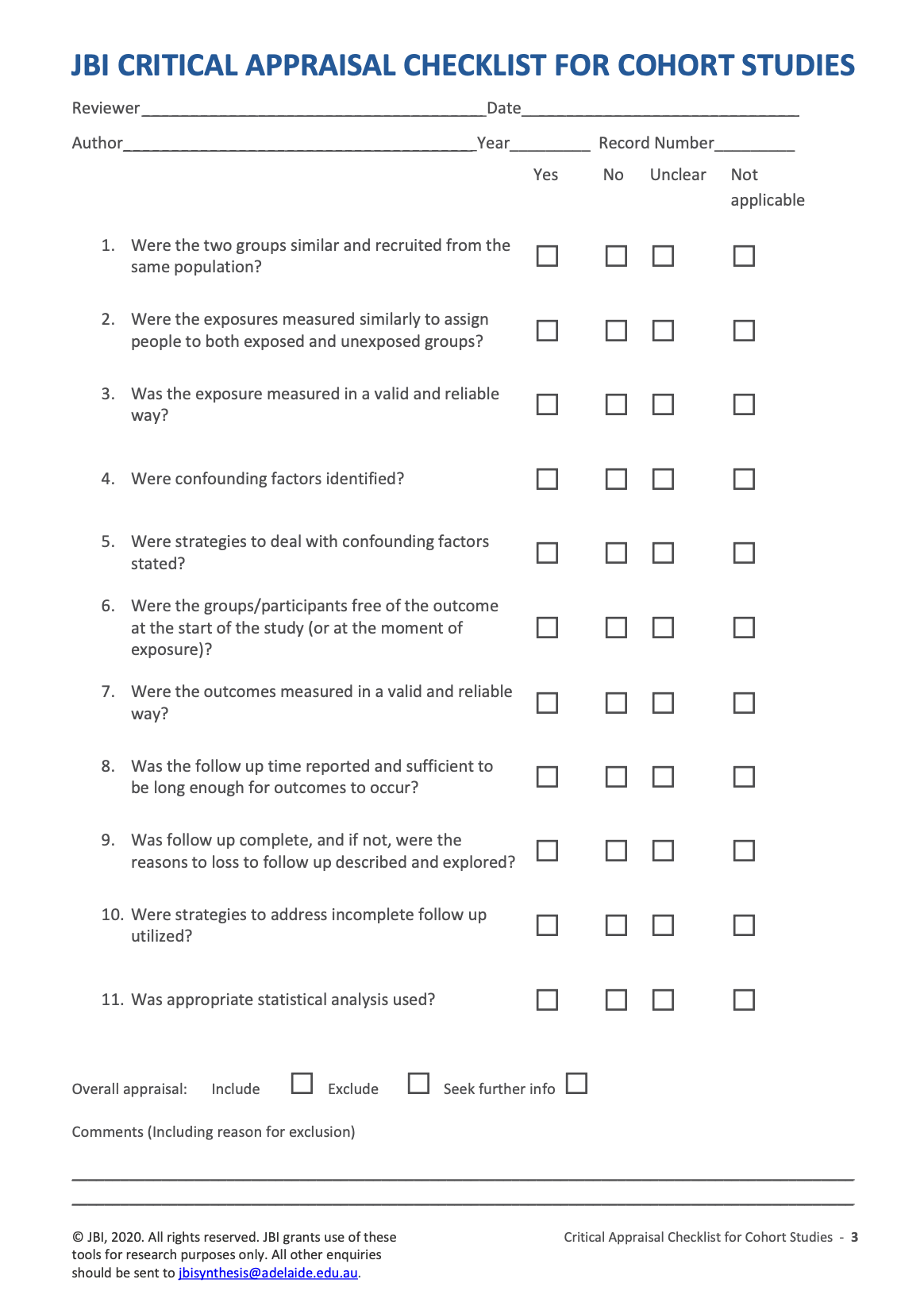
 Supplementary Table 10: JBI Cohort checklist

## Supplementary Table 11: JBI results - Cohort studies

| **Author** | 1.Were the two groups similar and recruited from the same population? | 2.Were the exposures measured similarly to assign people to both exposed and unexposed groups? | 3.Was the exposure measured in a valid and reliable way? | 4.Were confounding factors identified? | 5.Were strategies to deal with confounding factors stated? | 6.Were the groups/participants free of the outcome at the start of the study (or at the moment of exposure)? | 7.Were the outcomes measured in a valid and reliable way? | 8.Was the follow up time reported and sufficient to be long enough for outcomes to occur? | 9. Was the follow up complete, and if not, were the reasons to loss to follow up described and explored? | 10. Were strategies to address incomplete follow up utilised? | 11. Was appropriate statistical analysis used? | **Score** |
| --- | --- | --- | --- | --- | --- | --- | --- | --- | --- | --- | --- | --- |
| **Physiotherapists** | | | | | | | | | | | | |
| van Melick, N., Hoogeboom, T. J., Pronk, Y., Rutten, B., van Tienen, T. G., Nijhuis-van der, Sanden, M. W. G.  van Cingel, R. E. H. | Yes | Yes | Yes | Yes | Yes | Yes | Yes | Yes | Yes | Yes | Yes | 11/11 |
